# Supplementary material for: Laboratory evaluation of twelve portable devices for medicine quality screening
Source: PLoS Negl Trop Dis. 2021 Sep 30;15(9):e0009360. doi: 10.1371/journal.pntd.0009360 (PMC8483346; doi:10.1371/journal.pntd.0009360)
Supplement: S1 Appendix — (PDF) [file pntd.0009360.s001.pdf]

S1 Appendix. Brief Technology Descriptions

Colorimetric assay: Chemical reactions between a chemical of interest ("the analyte") and a reagent can lead to products that yield observable color changes. For example, iodine reacts with starch to create a dark-blue color. Functional groups on molecules, such as APIs or excipients, can react with different chemical reagents to create specific color changes. The presence of an API or excipient can be tested based on the color changes for specific reagents. Multiple reagents can be run in parallel to create patterns of colors that can target a wide array of analytes [1].

Lateral Flow Immunoassay: Antibodies are proteins that can tightly bind to specific molecular structures. In lateral flow immunoassays, a sample solution is loaded into an absorbent pad that is secured to the bottom portion of a plastic cartridge. The pad contains primary antibodies bound to colored or fluorescent species, such as gold nanoparticles. The bound antibodies are known as derivatized antibodies. When a sample droplet is deposited onto this pad, the derivatized antibodies bind to the analyte of interest in the solution (e.g. artesunate), if it is present. The solution containing the analytes bound to the derivatized antibodies travels up a membrane placed at the center of the cartridge. This membrane contains a line of secondary stationary antibodies bound to its surface known as the test line. As the solution travels through this test line, the complex of analyte bound to the derivatized antibody attaches to the stationary antibody, creating an observable color change on the test line for the user to see. The analyte acts as a bridge between the stationary antibody and the derivatized antibody . If no analyte is present, the derivatized antibodies are not captured by the test line, travel to the top of the cartridge past the test line, and no color change occurs at the test line [2].

Liquid Chromatography: Molecules can be separated based on their size and solubility. Four critical components are required in a liquid chromatography experiment: a mobile phase solvent pump, an injector, a stationary phase column, and a detector. The mobile phase solvent is pumped through the injector, column, and detector. As a sample is injected, the mobile phase carries the sample to the column. Inside the column, different analytes are separated based on differences in their chemical properties, such as size and solubility, between the mobile and stationary phases. The mobile phase is continuously pumped through the column. Compounds that are more soluble in the mobile phase than in the stationary phase leave the column the quickest. The compounds that bind more strongly to the stationary phase in the column remain in the system longer. As they are progressively separated, the compounds leave the column and enter the detector. Multiple types of detectors exist, but the most common is an absorbance detector. In absorbance detectors, the separated molecules travel through a beam of light, the analytes absorb the light, and the light that makes it through the sample can be measured. The amount of light absorbed is correlated to the amount of analyte that passed through the detector. The time the analyte remains on column and is detected after injection correlates to the chemical structure of the analyte [3,4].

Mass Spectrometry: Molecules range in mass because they can be composed of different numbers and types of atoms. During a mass spectrometry experiment, charges are added or removed from the molecules in the sample to create ions. Ions have a charge and can be transported or separated by electric fields. Ions are also vaporized into the gas phase before entering the mass spectrometer for separation. The mass of each ion contributes to how they travel through the mass spectrometer. For example, heavier ions travel slower than lighter ions and can be separated in time-of-flight mass spectrometers. Another common instrument known as a quadrupole mass spectrometer can separate compounds because different masses have ions of different stabilities in oscillating electric fields. These electric fields can be tuned to select ions at a specific mass and exclude others. Ions that have a motion path that is not stable are ejected before reaching the detector. After the ions have been separated, they hit a detector and are measured. In a time-of-flight instrument, the travel time of the

ion to reach the detector determines the mass of the ion. In quadrupole instruments, the selected electric field tune determines the mass of the ion observed [3,5].

Microfluidics: Traditional chemistry and biochemistry require multiple reaction vessels, storage vessels, purification protocols, and detection experiments all conducted in separate steps. Microfluidics streamlines experiments with a single chip that can perform the reactions, purify the samples, and/or detect the product. These chips are designed to operate in the microliter-volume or micrometer-channel scale. Microfluidics are the chemistry and biochemistry equivalent to printed circuit boards in electronics . Microfluidic devices minimize the number of consumables required and the bulk of the equipment necessary by putting all the steps in a single chip as well as speed along the process with minimal user input [6].

Mid-Infrared Spectroscopy (MIR): The mid-infrared wavelengths of light range from 2500 nm to 25000 nm (4000 cm^{-1} to 400 cm^{-1}). Different types of chemical bonds within molecules can absorb different ranges of infrared light. The infrared light creates vibrations caused by specific chemical bonds. For example, a bond between water and hydrogen absorbs infrared light at around 3550 – 3230 cm^{-1}. There is also a certain fingerprint region from 1500 – 500 cm^{-1} that can help identify a molecule through pattern matching. In its most basic operation mode, a MIR instrument shines infrared light at the sample. The sample absorbs portions of the infrared light and the light that is either reflected or transmitted through the sample is measured [3,7].

It is common in infrared spectroscopy to use a technique known as Fourier transform (FT) to process data. FT helps simplify and expand the capabilities of an infrared instrument. In traditional spectroscopy, specific wavelengths of light are isolated and shot at the sample. After measuring the intensity at that single wavelength, the instrument moves onto the next adjacent wavelength and the process repeats until the entire wavelength range is scanned. In FTIR spectrometers, a Michelson interferometer sweeps the entire wavelength of light simultaneously. The FT algorithm converts the time it takes to sweep the different intensities of light to the frequency or wavelength domain to see which bands of light were most absorbed by the sample [8].

Near-Infrared Spectroscopy (NIR): The near-infrared wavelengths of light range from 750 nm to 2500 nm (14000 cm^{-1} to 4000 cm^{-1}), closer to visible red light than MIR. NIR fundamentals and operation are very similar to the MIR techniques outlined above. In basic operation, a NIR instrument shines infrared light at the sample. The sample absorbs portions of the infrared light and the light that is either reflected or transmitted through the sample is measured. The absorption bands are wider in NIR than MIR and may require more computational analysis to extract information about the chemical bonds that are detected [3,9].

Raman Spectroscopy: The vibrations in bonds of a molecule can scatter light emitted by a laser. There are three types of scattering. Rayleigh scattering is at the same wavelength as the excitation source or laser and the most abundant scattering of the light. Light can also be scattered at longer or shorter wavelengths compared to the excitation source. Shorter wavelength scattering is known as anti-Stokes scattering; longer wavelength scattering is known as Stokes scattering. Raman spectroscopy only looks at Stokes scattering by different chemical bonds within the molecule. These vibrations, depending on the chemical bond, scatter light at different wavelengths. Stokes scattering ranges from 2500 nm to 100000 nm (4000 cm^{-1} to 10 cm^{-1}) In basic operation, a laser is shot at the sample. The samples scatters light, and the Stokes scattering is measured perpendicular to the path of the laser [3,10].

Thin Layer Chromatography (TLC): Molecules can be separated based on their size and solubility. Three critical components are required in TLC: the stationary phase, mobile phase, and a detection method to “reveal” the plates. The stationary phase for TLC is typically powdered silica gel applied

onto a hard substrate, like glass or aluminum, as a thin layer. A drop of sample is placed about a centimeter from the bottom of the TLC plate on the silica gel. The bottom edge of the TLC plate is submerged into the mobile phase. The mobile phase travels up the TLC plate through capillary action. The sample dissolves into the mobile phase as it passes by and travels with the mobile phase towards the top of the plate. Larger molecules or molecules that bind better to the stationary phase travel slower than smaller molecules or compounds that do not bind to the stationary phase well. Once the edge of the mobile phase reaches the top, the TLC plate is removed from the mobile phase and left to dry. As the mobile phase dries from the plate, the analytes are captured, which prevents them from traveling any more on the plate. Chemical treatments or light sources, such as ultraviolet lamps, can be used to see how far up the TLC plate the molecules migrated. The farther the analyte spot is up on the TLC plate, the smaller the molecule and less it bound to the stationary phase. The size and color intensity of the spots also correlates to the concentration of the analyte [3,11].

References

1. Philp M, Fu S. A review of chemical 'spot' tests: A presumptive illicit drug identification technique. Drug Test Anal. John Wiley and Sons Ltd; 2018;10: 95–108. doi:10.1002/dta.2300
2. Koczula KM, Gallotta A. Lateral flow assays. Essays Biochem. Portland Press Ltd; 2016;60: 111–120. doi:10.1042/EBC20150012
3. Skoog DA, Holler FJ, Crouch SR. Principles of Instrumental Analysis [Internet]. 6th ed. Thomson Brooks/Cole; 2007. Available: https://books.google.com/books?id=GrOsQgAACAAJ
4. Dorsey JG, Cooper WT, Siles BA, Foley JP, Barth HG. Liquid Chromatography: Theory and Methodology [Internet]. 1996. Available: https://pubs.acs.org/sharingguidelines
5. Glish GL, Vachet RW. The basics of mass spectrometry in the twenty-first century. Nature Reviews Drug Discovery. Nature Publishing Group; 2003. pp. 140–150. doi:10.1038/nrd1011
6. Bhatt G, Kumar S, Sundriyal P, Bhushan P, Basu A, Singh J, et al. Microfluidics overview. Microfluidics for Biologists: Fundamentals and Applications. Springer International Publishing; 2016. pp. 33–83. doi:10.1007/978-3-319-40036-5_2
7. Haas J, Mizaikoff B. Advances in Mid-Infrared Spectroscopy for Chemical Analysis. Annu Rev Anal Chem. Annual Reviews; 2016;9: 45–68. doi:10.1146/annurev-anchem-071015-041507
8. How an FTIR Spectrometer Operates - Chemistry LibreTexts [Internet]. [cited 3 Apr 2020]. Available: https://chem.libretexts.org/Bookshelves/Physical_and_Theoretical_Chemistry_Textbook_Maps/Supplemental_Modules_(Physical_and_Theoretical_Chemistry)/Spectroscopy/Vibrational_Spectroscopy/Infrared_Spectroscopy/How_an_FTIR_Spectrometer_Operates
9. Pasquini C. Near infrared spectroscopy: A mature analytical technique with new perspectives – A review. Analytica Chimica Acta. Elsevier B.V.; 2018. pp. 8–36. doi:10.1016/j.aca.2018.04.004
10. Bumbrah GS, Sharma RM. Raman spectroscopy – Basic principle, instrumentation and selected applications for the characterization of drugs of abuse. Egyptian Journal of Forensic Sciences. Egyptian Forensic Medicine Authority; 2016. pp. 209–215. doi:10.1016/j.ejfs.2015.06.001
11. Bele AA, Khale A. AN OVERVIEW ON THIN LAYER CHROMATOGRAPHY. Int J Pharm Sci Res. 2010;2: 256–267. doi:http://dx.doi.org/10.13040/IJPSR.0975-8232.2(2).256-67
