# Supplementary material for: Laboratory evaluation of twelve portable devices for medicine quality screening
Source: PLoS Negl Trop Dis. 2021 Sep 30;15(9):e0009360. doi: 10.1371/journal.pntd.0009360 (PMC8483346; doi:10.1371/journal.pntd.0009360)
Supplement: S2 Appendix — (PDF) [file pntd.0009360.s002.pdf]

S2 Appendix. Notable device configurations and constraints

Each device was used in its factory configuration with three important exceptions. First, a cover was built for the sampling interface of the MicroPHAZIR RX spectrometer to avoid ambient light from entering the detector when the tablets tested were smaller than the sampling interface. Second, the QDa was equipped with a Waters Reagent Manager solvent pump and a Rheodyne Model 7125 six port injector valve for flow injection analysis. Third, the Neospectra 2.5 detector unit was provided with a light source (Avantes AVALIGHT-HAL-MINI) and fiber optic sampling probe (Thor Labs FG550LEC-YCABLE-SP) provided by the manufacturer

Notable constraints to the devices of this study were as follows. First, for medicine testing with the C-Vue liquid chromatograph, only the mercury lamp detector was used because the API tested was not detectable with the zinc lamp during protocol development. Second, for testing with the Minilab kit, only thin layer chromatography (TLC) experiments were conducted, and disintegration and weighing tests were not performed as they were viewed as being outside of the scope of the project. Third, for PAD testing, no imaging software was used for analysis of the cards, as the software was still in the development stage. Fourth, there were a limited number of the single-use PADs and RDTs. The few PADs and RDTs available limited the number of replicates possible during experiments .
