# Supplementary material for: Laboratory evaluation of twelve portable devices for medicine quality screening
Source: PLoS Negl Trop Dis. 2021 Sep 30;15(9):e0009360. doi: 10.1371/journal.pntd.0009360 (PMC8483346; doi:10.1371/journal.pntd.0009360)
Supplement: S3 Appendix — (PDF) [file pntd.0009360.s003.pdf]

S3 Appendix. Photographs of the devices.

Fig S3 A. Images of the Agilent 4500a single reflection Fourier transform infrared (FTIR) spectrometer. 2
Fig S3 B. Images of the C-Vue liquid chromatograph. 3
Fig S3 C. Images of the MicroPHAZIR RX near infrared (NIR) spectrometer. 4
Fig S3 D. Images of the Minilab thin layer chromatography (TLC) kit. 5
Fig S3 E. Image of the Neospectra 2.5 NIR spectrometer with sampling probe and light source. 6
Fig S3 F. Images of the NIR-S-G1 beta version (NIRscan) NIR spectrometer with smartphone used to control the device. 7
Fig S3 G. Images of the single use Paper Analytical Devices (PADs). 8
Fig S3 H. Image of the PharmaChk microfluidic device. 9
Fig S3 I. Images of the Progeny Raman spectrometer. 10
Fig S3 J. Image of the single use Rapid Diagnostic Tests (RDTs). 11
Fig S3 K. Images of the Truscan RM Raman spectrometer. 12
Fig S3 L. Images of the QDa mass spectrometer. 13

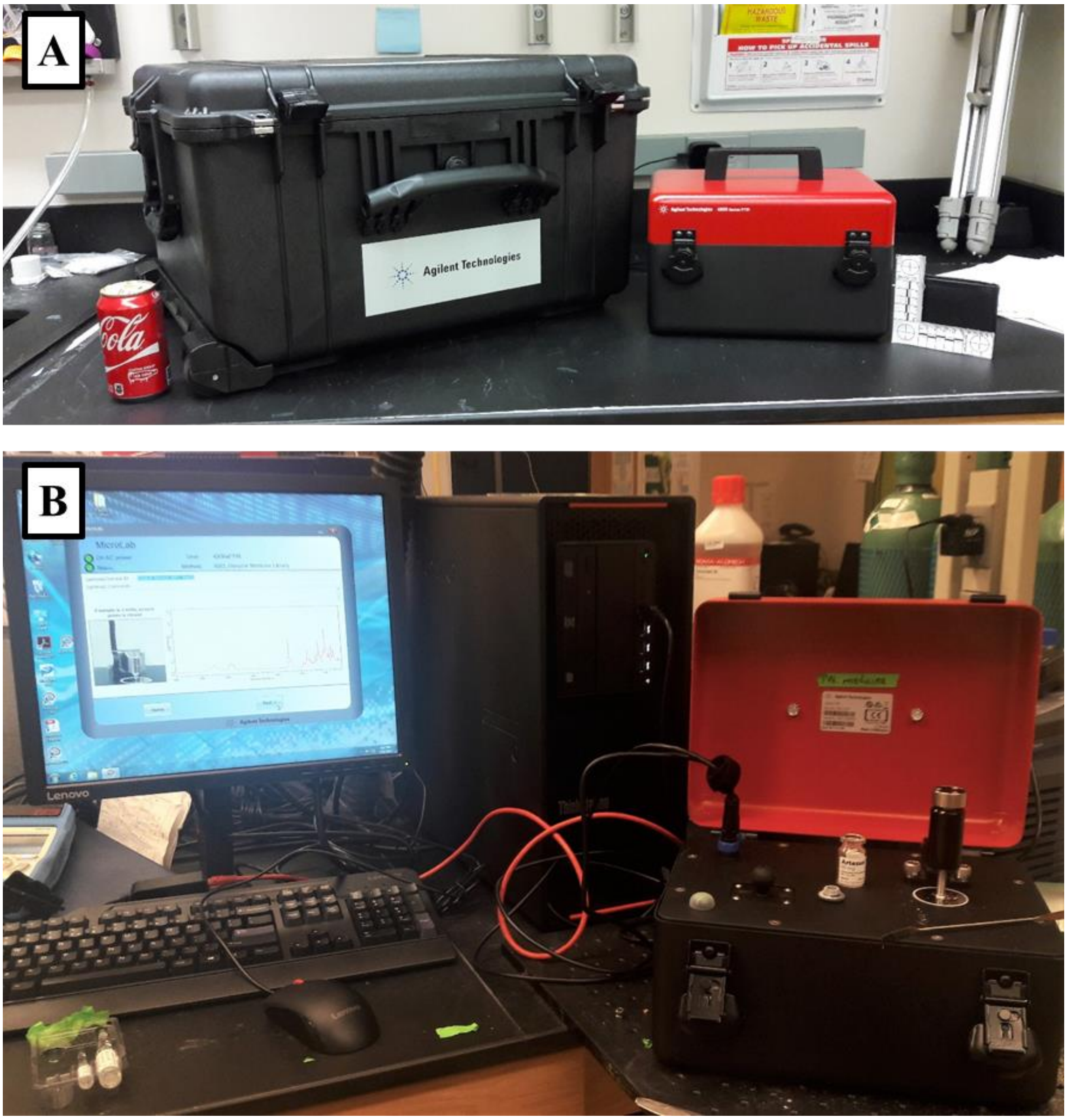

Fig S3 A. Images of the Agilent 4500a single reflection Fourier transform infrared (FTIR) spectrometer.
(A) From left to right, a can of soda as a size reference, the carrying case for the 4500a instrument, and the Agilent 4500a instrument. **(B)** The Agilent 4500a FTIR set up with the master computer testing Artesunate®.

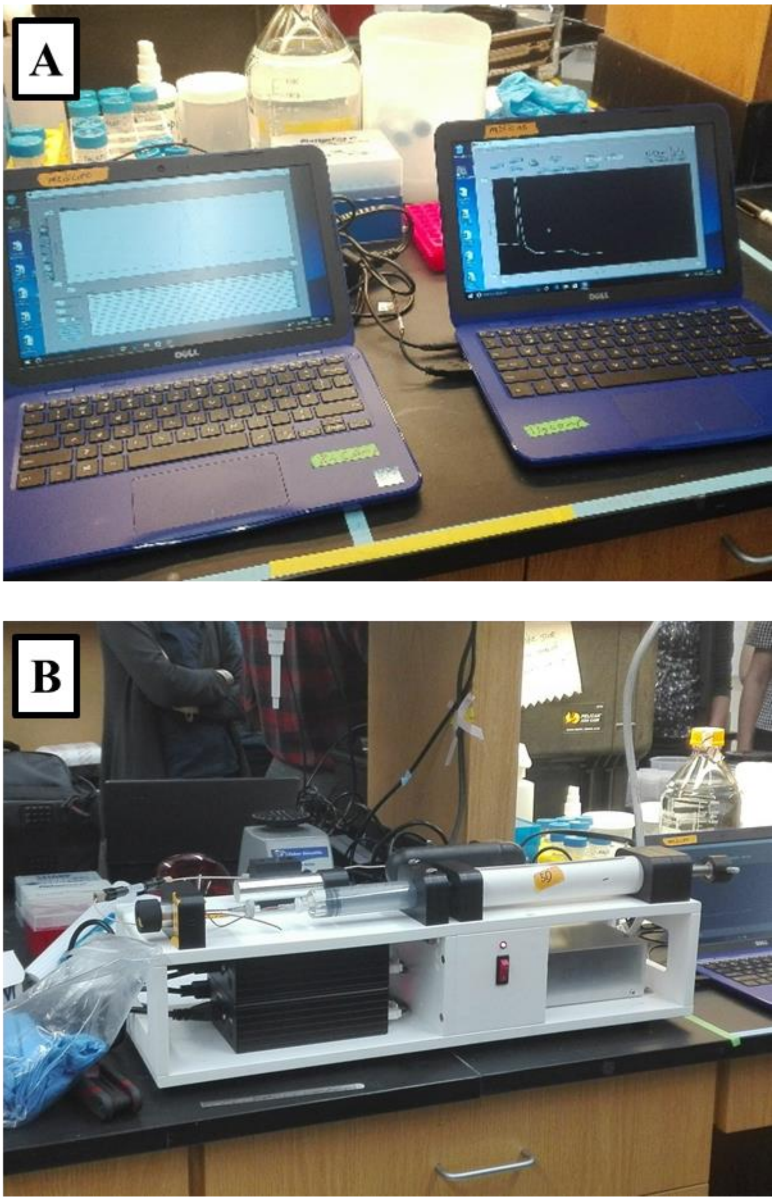

Fig S3 B. Images of the C-Vue liquid chromatograph.
(A) The computers used to record the chromatograms for the C-Vue device. Each computer records the signal from a separate detector on the C-Vue. **(B)** An image of the C-Vue chromatograph set-up.

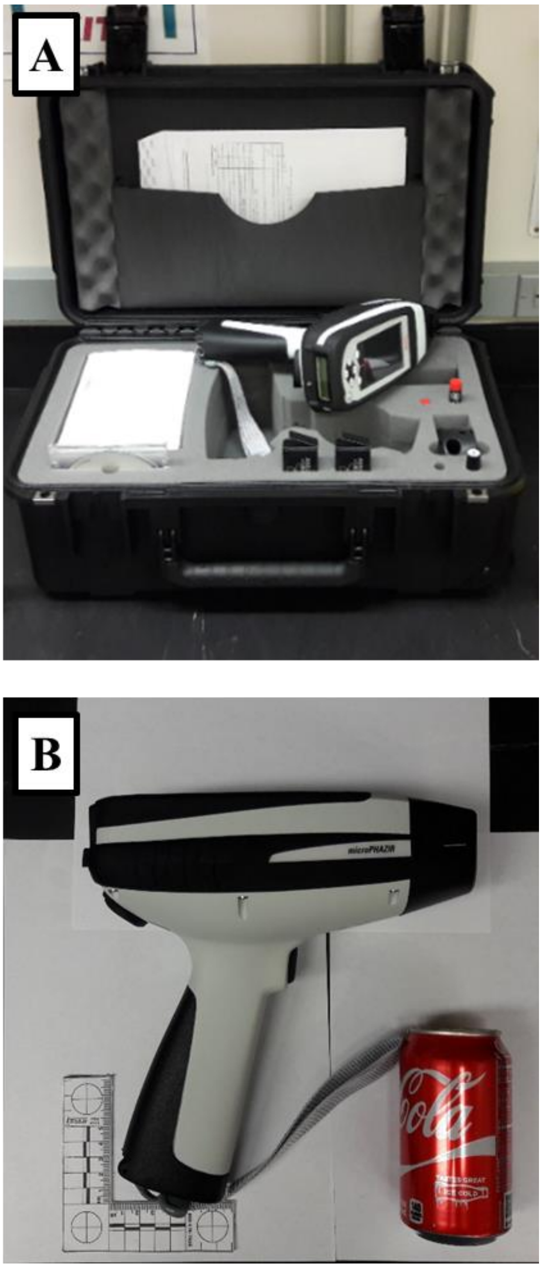

Fig S3 C. Images of the MicroPHAZIR RX near infrared (NIR) spectrometer.
(A) The MicroPHAZIR RX instrument laid in the center of its carrycase. **(B)** The MicroPHAZIR RX laid on this side next to a soda can for a size reference and a ruler in centimeters.

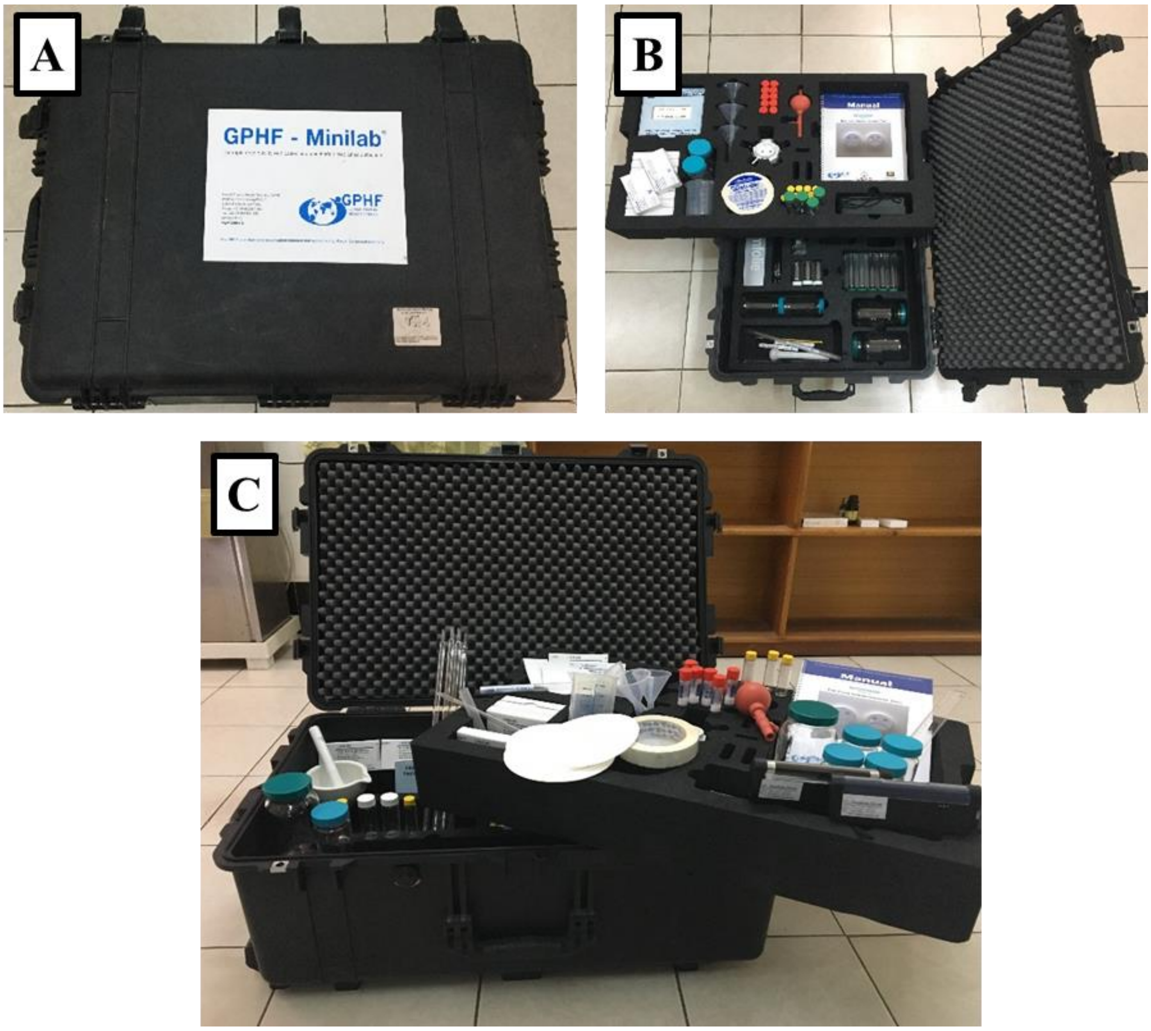

Fig S3 D. Images of the Minilab thin layer chromatography (TLC) kit.
(A) Image of the top cover of the Minilab kit carrying case. **(B)** Image of the top two layers of the Minilab TLC kit. **(C)** Image of the top two layers of the Minilab TLC kit expanded.

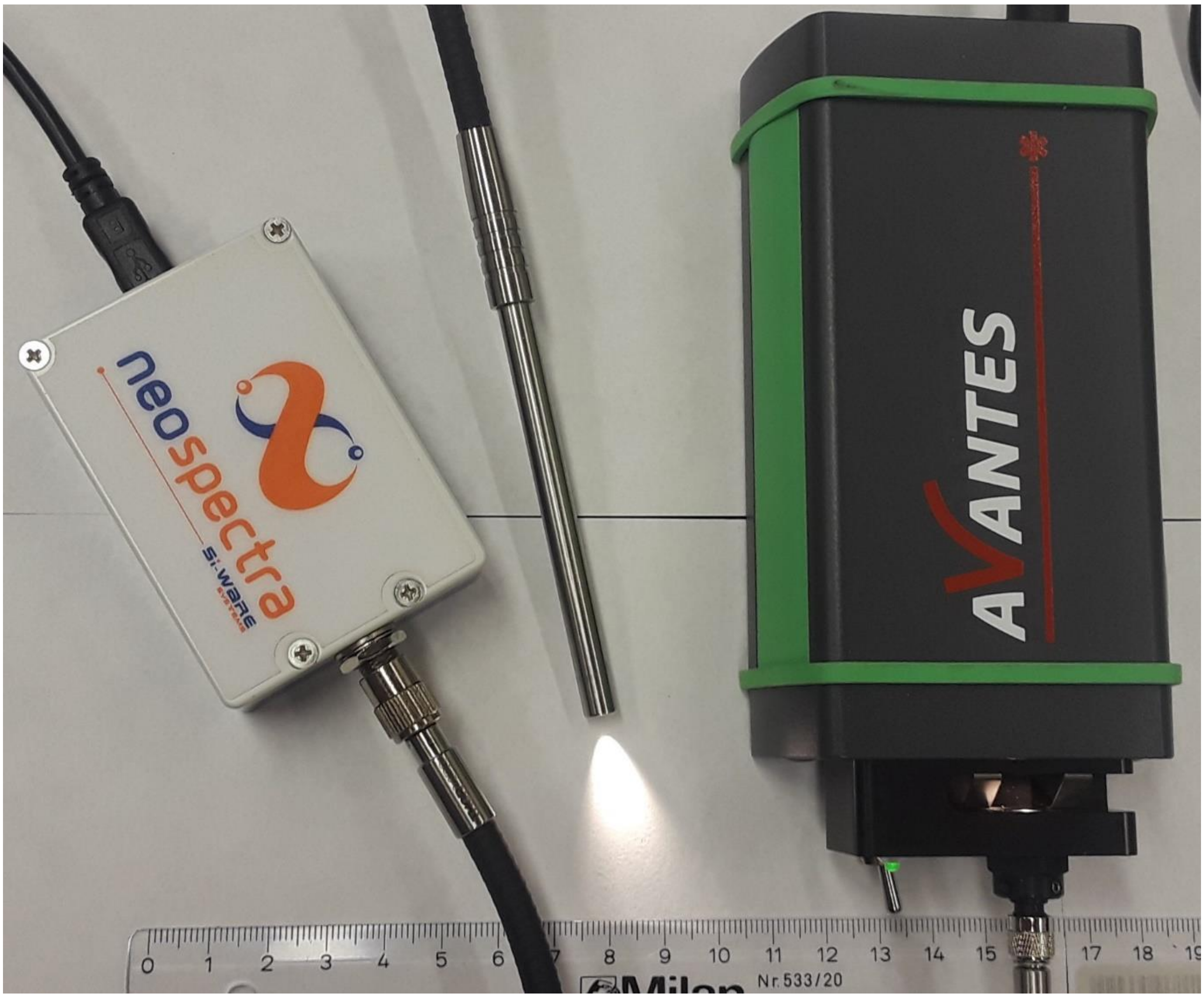

Fig S3 E. Image of the Neospectra 2.5 NIR spectrometer with sampling probe and light source. Ruler scale is in centimeters. Left unit is the detector, center rod is the sampling probe, and right unit is the light source.

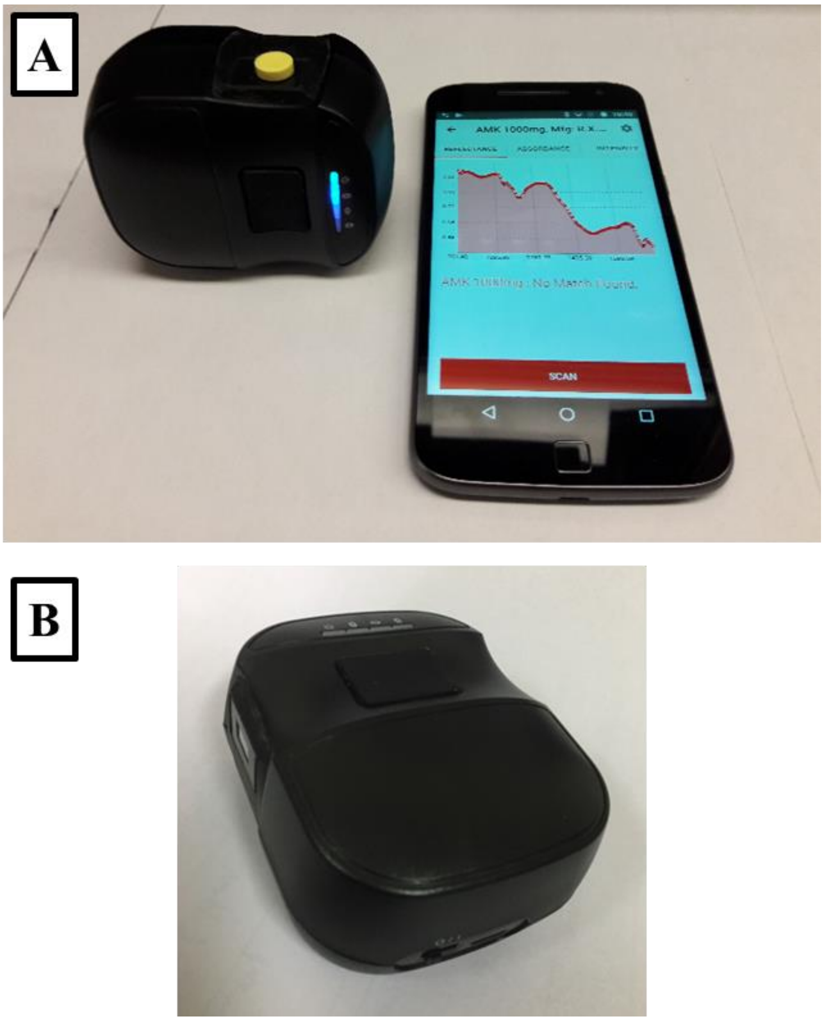

Fig S3 F. Images of the NIR-S-G1 beta version (NIRscan) NIR spectrometer with smartphone used to control the device.
(A) The NIRscan spectrometer on the left testing a falsified Coartem® tablet with the result displayed on the smartphone on the right. **(B)** A side image of the NIRscan spectrometer.

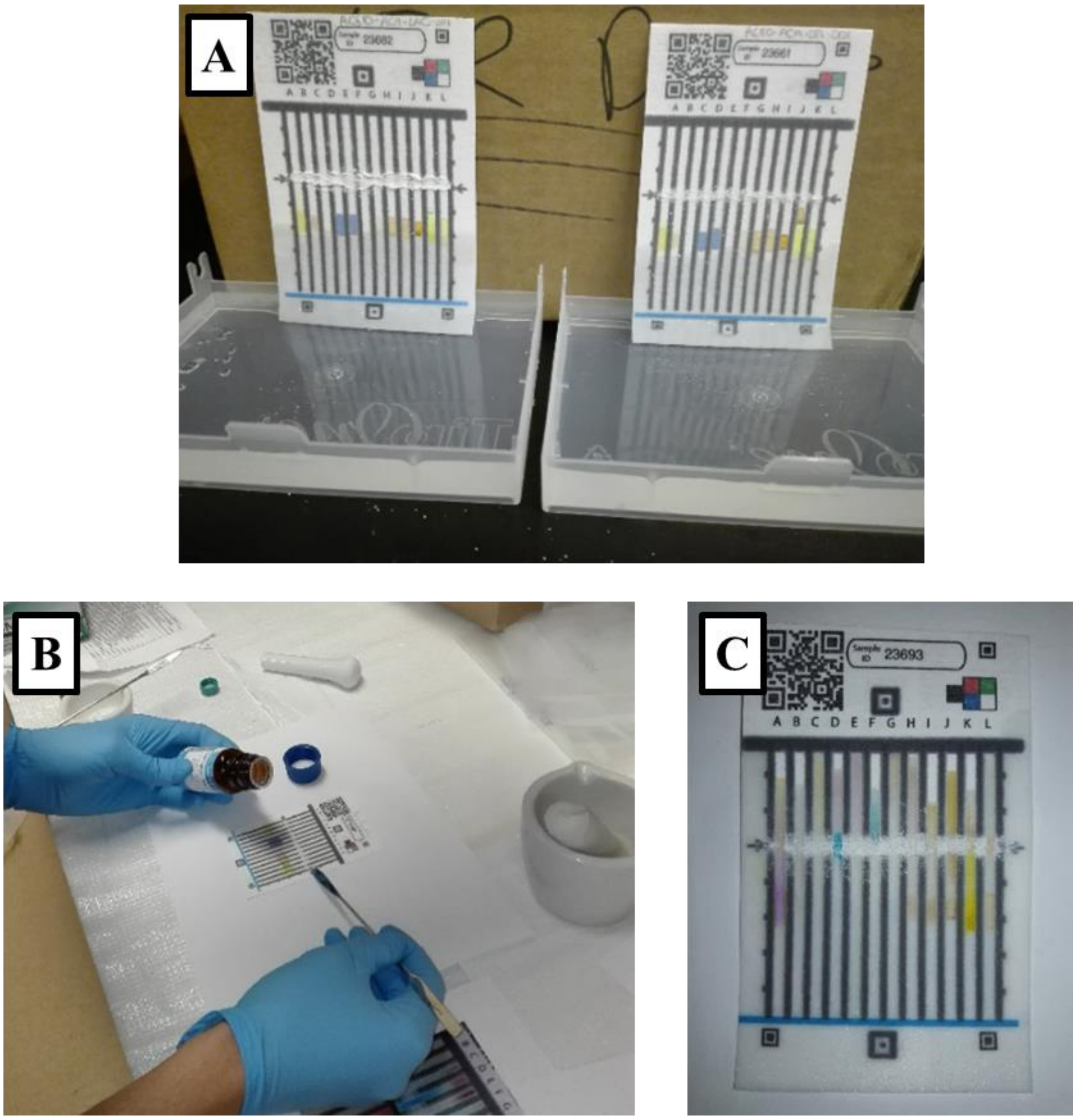

Fig S3 G. Images of the single use Paper Analytical Devices (PADs).
(A) Image of side by side PADs with the water rising by capillary action through the card. **(B)** Image of the PAD being prepared for testing. **(C)** Image of a completed PAD experiment, ready for analysis.

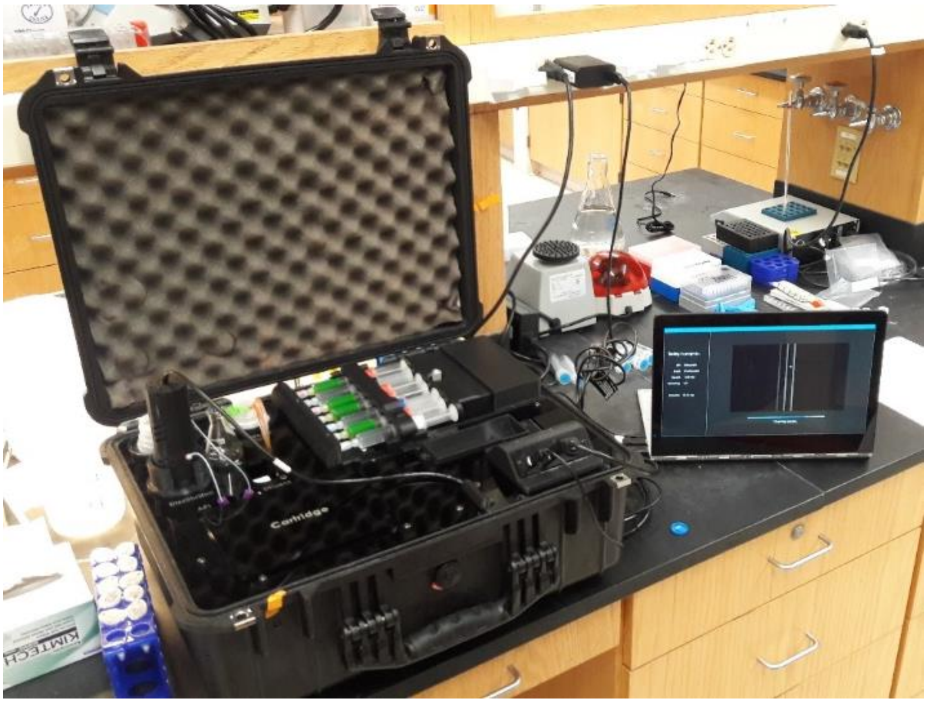

Fig S3 H. Image of the PharmaChk microfluidic device.
Unit of the left is the hard shell case with the experimental setup that includes the microfluidic chip, syringe pumps, detector, and other hardware. Computer tablet on the right is the user interface for the PharmaChk that controls the device, guides the setup, and displays the results.

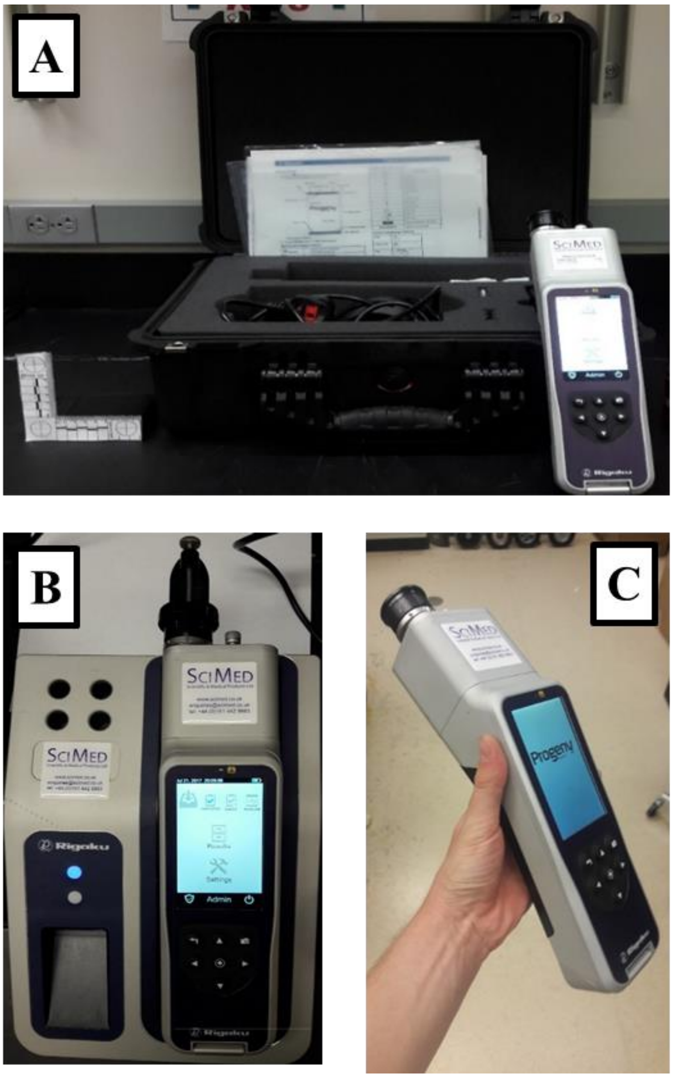

Fig S3 I. Images of the Progeny Raman spectrometer.
(A) Image of the Progeny Raman spectrometer leaning against the carrying case for the instrument. Ruler scale is in centimeters. **(B)** The Progeny Raman spectrometer docked to the charging station and the tablet holder attached to the sampling interface of the instrument. **(C)** An image of the side of the Progeny Raman spectrometer being held in the operator’s hand.

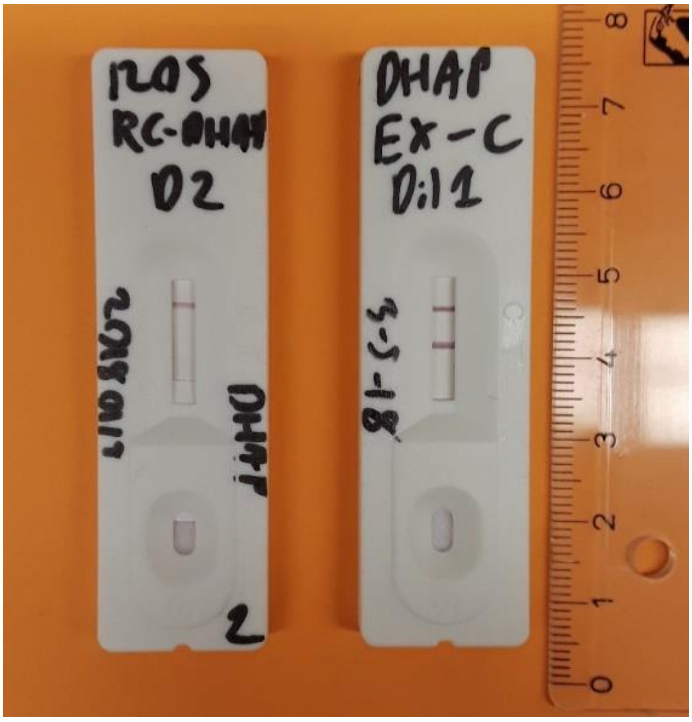

Fig S3 J. Image of the single use Rapid Diagnostic Tests (RDTs).
Ruler scale is in centimeters. RDT on the left is a good quality DHAP with a true negative result. RDT on the right is a cellulose only tablet with a true positive result.

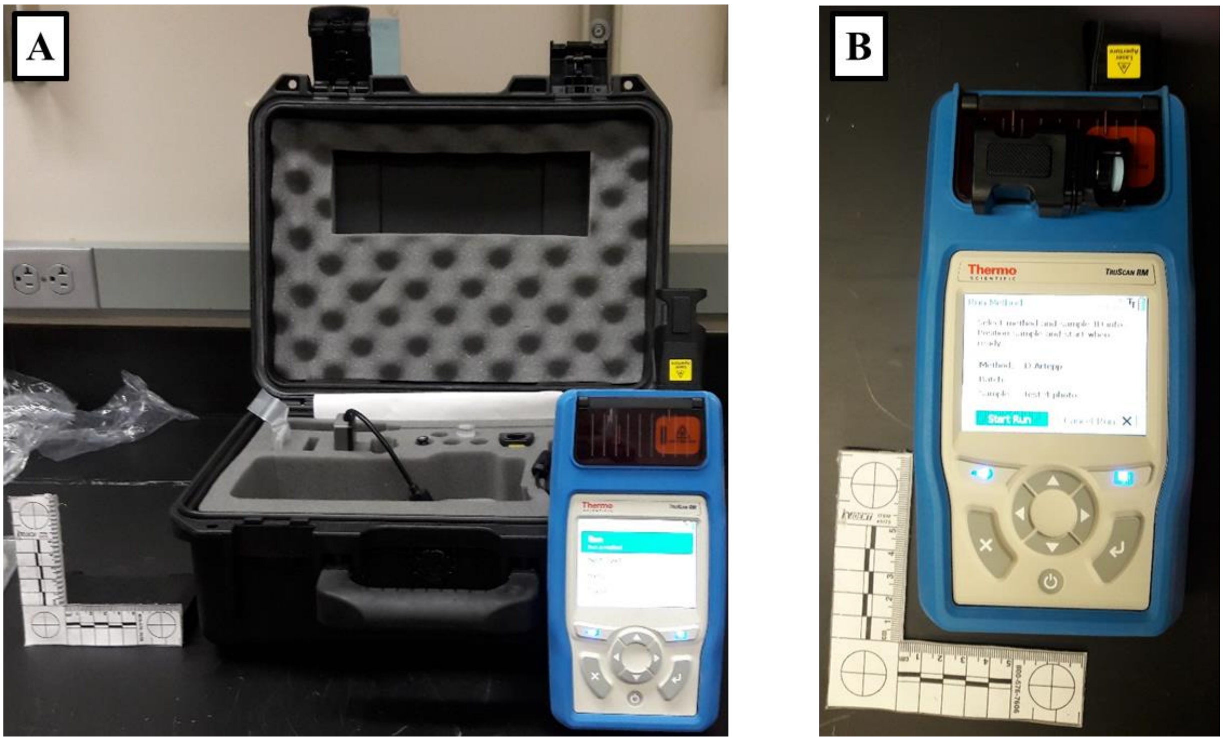

Fig S3 K. Images of the Truscan RM Raman spectrometer.
Ruler scale is in centimeters. **(A)** Image of the Truscan RM Raman spectrometer leaning against the carrying case for the instrument. **(B)** Image of the front face of the Truscan RM Raman spectrometer with the tablet holder placed on top of the instrument.

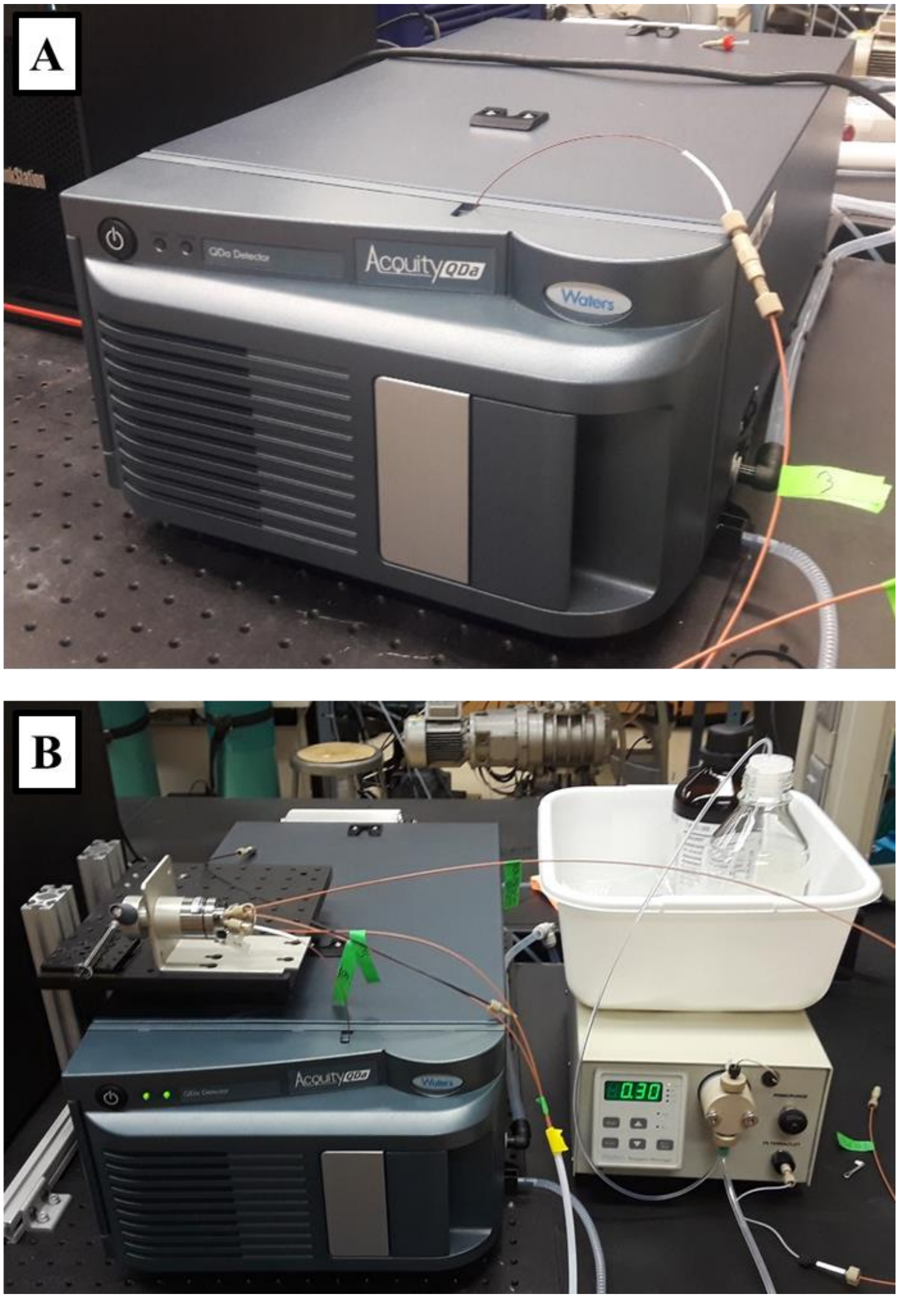

Fig S3 L. Images of the QDa mass spectrometer.
(A) Image of the standalone QDa mass spectrometer. **(B)** Image of the QDa mass spectrometer set-up used for this study. The unit on the bottom right is solvent pump with the solvent bottles on top in secondary containment. On top of the QDa mass spectrometer is the six-port injector for flow injection analysis.
