# Supplementary material for: Laboratory evaluation of twelve portable devices for medicine quality screening
Source: PLoS Negl Trop Dis. 2021 Sep 30;15(9):e0009360. doi: 10.1371/journal.pntd.0009360 (PMC8483346; doi:10.1371/journal.pntd.0009360)
Supplement: S4 Appendix — (PDF) [file pntd.0009360.s004.pdf]

S4 Appendix. Device descriptions.

Table S4 A. Overview of the 4500a single reflection FTIR spectrometer. 2
Table S4 B. C-Vue liquid chromatograph overview. 3
Table S4 C. MicroPHAZIR RX NIR spectrometer overview. 4
Table S4 D. Minilab thin layer chromatography kit overview. 5
Table S4 E. Neospectra 2.5 NIR spectrometer overview 6
Table S4 F. NIR-S-G1 (NIRscan) NIR spectrometer overview 7
Table S4 G. Paper Analytical Devices (PADs) single use device overview. 8
Table S4 H. PharmaChk microfluidic device overview 9
Table S4 I. Progeny Raman spectrometer overview. 10
Table S4 J. Rapid Diagnostic Tests (RDTs) single use device overview. 11
Table S4 K. Truscan RM Raman spectrometer overview. 12
Table S4 L. QDa mass spectrometer overview. 13

Table S4 A. Overview of the 4500a single reflection FTIR spectrometer.

**Manufacturer/Developer**	Agilent<br>https://www.agilent.com/en/products/ftir/ftir-compact-portable-systems/4500-series-portable-ftir
**Technology overview**	The 4500a is a portable bench-top mid-infrared Fourier transform spectrometer. All the optics, including the Michelson interferometer and sampling window, are contained within the instrument. An external command module, such as a Windows-based smartphone or a computer, controls the instrument. In our study, we used a desktop computer in the laboratory evaluation and a laptop computer for the field evaluation. The device only can be used with powdered samples that are placed and pressed onto a diamond attenuated total reflectance sample window. The instrument compares the experimental spectrum recorded with spectra from the stored library pre-selected by the user. The software outputs all the possibilities of the sample's identity along with its "hit quality". Ensuring consistent sample pressure of the attenuated reflectance accessory is important for collection of spectral signals.<br>**The device cannot operate in the field without a computer. A Windows based smartphone could be used (but was not tested in this study).**<br>**Samples are destroyed in the analysis.**
**APIs tested**	All seven APIs/combination of APIs
**Specifications**	*Dimensions:* 220 mm x 290mm x 190mm (instrument only)<br>*Weight:* 6.8kg (instrument only)<br>*Power source:* Li-ion battery 11.1V 7.8Ah (internal battery)<br>*Spectral range:* 4000 cm^{-1} to 650 cm^{-1} (2500 nm to 15384 nm)<br>*Internal file storage size:* Master computer/phone dependent<br>*Library/data file size:* Entire library for this study 53 kB; Data file about 30 kB each
**Cost***	• One Agilent 4500 unit: ~US$ 31,067<br>• Laptop computer: ~US$ 500<br>Recurring costs:<br>• Required consumable material: ~US$ 0.09 per run
**Reference library**	Prior to library spectra collection, a scanning method must be created for the instrument. Scanning methods control the number of scans and calculations executed by the instrument. Library spectra are tied to the scanning methods that were used. Library spectra cannot be transferred from one scanning method to another if the parameters of the method are different.
**Calibration considerations**	Performance and stability tests should be performed by the user (minimum annually, but more often is recommended). Minimum annual laser frequency calibration test should be performed using a polystyrene test sample provided by the manufacturer.
**Testing abilities**	Screening falsified medicine is potentially possible for all medicines, provided that formulation-specific reference libraries are available. The current algorithms utilized for the device have not been developed for the detection of substandard medicines. Algorithms should be developed on an API-specific basis to enhance detection. Formulation-specific device.
**Method consideration for the present study**	The 4500a FTIR is set up to return the six highest matches of the sample spectrum to the reference library entry. The following procedure was agreed with laboratory evaluators to interpret the results: if the tested medicine appeared in the six highest matches with a "hit quality" score > 0.9, the sample would "pass". If the tested medicine appeared in the six highest matches with a hit quality of < 0.9, it should be flagged as suspicious and the test repeated as per protocol used for other spectrometers.

* The costs reported here do not include VAT

Table S4 B. C-Vue liquid chromatograph overview.

**Manufacturer/Developer**	C-Vue Chromatography<br>http://www.cvuechromatography.com/
**Technology overview**	The C-Vue is a portable liquid chromatograph that can separate and detect APIs based on their chemical structure. The basic components include a pump, a six-port injector, a column, two detectors, and a computer for data recording. From the injector, the solvent flow goes towards to the column and then onto two detectors connected in series. One absorbance detector uses a zinc lamp (214 nm) and the other detector uses a mercury lamp (254 nm). To record data from both detectors at the same time, two computers are required. Samples are loaded into the injector via a syringe through a syringe filter. To initialize injection and record the LC run, the user must turn the valve on the injector and simultaneously hit the "Start" button in the C-Vue software to record data . Once data has been collected, the results can be immediately analyzed through the C-Vue software to obtain peak retention time, height, and area information manually. This data can be processed directly on the C-Vue software or exported to other data analysis software.<br>**The device cannot operate in the field without computer(s).**<br>**Samples are destroyed (dissolved) in the analysis.**
**APIs tested**	ACA, OFLO, SMTM*
**Specifications**	*Dimensions:* 20.3 cm x 20.3 cm x 61 cm<br>*Weight:* 21.8 kg (with battery and Pelican case)<br>*Power source:* Mains as tested or optional 14 amp/hr battery for remote operation<br>*Light sources*: 214nm (Zn) and 254nm (Hg)<br>*Internal file storage size:* Master computer dependent<br>*Library/data file size:* Library N/A; About 2 kB per minute of experiment
**Cost**†	***Upfront cost***<br>• C-Vue with 214-nm detector: ~US$ 4,950<br>• Chromatographic column (Millipore Chromolith RP18e 25 x 4.6 mm): ~US$ 370<br>• Additional 254-nm detector: ~US$ 1,295<br>• Computer : ~US$ 500<br>• Tool and accessory kit for sample preparation : ~US$ 175<br>***Recurring costs***<br>• Battery replacement, if applicable (expected 5-years life): US$ 60<br>• Maintenance cost (expected for 5-years life): US$ 150-1250<br>• Average required consumable cost: Total per sample = Calibration preparation (done once prior to the analysis of one or many of the same API samples) ~ US$ 2.41 + sample preparation and analysis ~ US$ 2.05 (includes one sample injection ~ US$ 0.98 and sample preparation which creates enough sample solution for multiple injections~ US$ 1.07). E.g. for 10 samples of the same API total cost ~ US$ 22.91
**Calibration considerations**	Calibration curves must be generated daily for every batch of runs.
**Method adaptation for the present study**	First, the mobile phase is prepared and loaded into the C-Vue liquid chromatograph. In this study, only water and methanol were used as solvents, with disodium phosphate as a buffer where applicable. Four-point calibration curves were prepared for each API. For co-formulated medicines containing a combination of APIs, both APIs were prepared in the same calibration solution, so calibration of both APIs could be done simultaneously. Of the seven APIs included in this study, only OFLO, SMTM, and ACA could be measured by the C-Vue liquid chromatograph with a response recorded on the mercury detector only. The zinc detector had no measurable response to the APIs at the concentration used in this study.
**Reference library**	None needed
**Testing abilities**	The instrument can detect changes of >=1-2% API concentration without any software or hardware changes or enhancements. The sensitivity to changes in API can be determined by statistically determining the injection repeatability mean (area under curve for API) and multiply the standard deviation by 3 to determine a discernible change. The instrument is not specific to a formulation.

* ART, AZITH, and DHAP could not be quantified because there was no signal response for these APIs up to 2,000 ppm level. Lumefantrine and piperaquine were detected and could be quantified, however, because these API are part of combinations of active ingredients, and their combined API artemether and dihydroartemisinin could not be detected with the C-Vue's current set-up, these medicines were not evaluated.

† The costs reported here do not include VAT

Table S4 C. MicroPHAZIR RX NIR spectrometer overview.

Manufacturer/Developer	Thermo Fisher Scientific<br>https://www.thermofisher.com/order/catalog/product/MICROPHAZIRRX
Technology overview	The MicroPHAZIR RX is a handheld near-infrared spectrometer. The device is controlled using an LCD screen and buttons on the top of the instrument. After the logging into the device, the user selects the reference library with which they would like to compare the sample, inputs the information about the sample, and scans. The device gives a pass/fail result. As an alternative to manually entering sample details, a barcode reader is built into the device to optimize metadata input. Although reference library spectra are collected by the device, creating and editing reference libraries entries can only be done on an external computer. For compiling reference libraries and exporting data from the device, a USB device connects the MicroPHAZIR RX with the external computer. On the computer, one software package communicates and transfers data to the device, while another software package generates the spectral libraries for the device.<br>**The device can operate in the field without a computer.**<br>**Samples are not destroyed during analysis.**
APIs tested	All seven APIs/combination of APIs
Specifications	*Dimensions:* 25 cm (H) x 23 cm (W) x 10 cm (D)<br>*Weight:* 1,250 grams<br>*Spectral range: 1600 nm to 2400 nm*<br>*Power source:* Li-ion battery<br>*Internal file storage size:* Not disclosed<br>*Library/data file size:* Up to 10,000 library entries; about 6,000 data scans
Cost*	*Capital cost*<br>• MicroPHAZIR RX basic unit: ~US$ 47,500†<br>*Recurring costs*<br>• Cost per run (consumables needed): ~US$ 0.04<br>• Battery replacement (expected 2-years life): ~US$ 253<br>• Replacement of light bulb (2-years life): ~US$ 150<br>• Approximate annual maintenance cost: ~US$ 75
Reference library	The user is guided to collect five spectra of the same sample, a process called collecting signatures. This allows for the introduction of some variability into the reference library collection, such as batch variation or sample position, to yield an average spectrum to compare against. Once the spectra are collected, they must be uploaded to a computer for processing (two software packages must be downloaded on the computer). The user selects the mathematical functions desired; the software then outputs a single library file that contains all the selected spectra to be uploaded to the MicroPHAZIR RX. Reference library and test spectra file types are specific to this instrument.
Calibration considerations	A "self-test" must be performed at least daily. A "calibration reference test" should be run to correct for any slight alignment changes (e.g. after the plastic nose cover is removed to change the light bulb, any time the instrument is exposed to large thermal excursions or mechanical vibrations or airplane transportation, or any time the instrument is not used for long periods of time. As part of Good Manufacturing Practices (GMP) requirement, an annual certification test must be performed. This requires the user to scan five standards provided by the manufacturer. After the test, the data files must be sent to the manufacturer's Customer Support unit for analysis and reporting back to the user. Formulation-specific device.
Method adaptation for the present study	Tablets that were significantly smaller than the diameter of the sampling window were placed under a sample cover constructed from the calibration sample holder (after discussion with the manufacturer's technical staff). This consisted of a plastic block mounted to the front nose of the device that reduced the ambient light entering the detector. This calibration sample holder contained an 18 mm diameter hole across which the calibration sample was placed, facing the sample window. This space was covered with electrical tape to make a darkened cavity where the sample table was located. The default built-in mathematical function was used for data processing. A vial holder mount was available but sold separately and was not purchased.
Testing abilities	Screening falsified medicine is potentially possible for all medicines, provided that formulation-specific reference libraries are available. The current algorithms available in the device have not been developed for substandard medicines detection. Algorithms should be developed on an API basis to enhance detection. Ability to test through transparent blisters and glass vials with reference library created using packaged samples.

* The costs reported here do not include VAT
† Ordering several devices from the manufacturer is subject to potential reduced purchase cost, according to the manufacturer

Table S4 D. Minilab thin layer chromatography kit overview.

**Manufacturer/Developer**	Global Pharma Health Fund E.V.<br>https://www.gphf.org/en/minilab/index.htm
**Technology overview**	The Minilab kit comes in a case with all the equipment necessary to conduct experiments to test the quality of 90 different APIs. After an extraction and a series of dilutions specific to each API, the diluted sample is spotted onto a thin layer chromatography (TLC) plate next to 2 reference standard solutions. The TLC plate base is submerged into a few millimeters of the mobile-phase liquid. After the TLC plate has been developed, the plate is subjected to API-specific detection methods, including ultraviolet light detection and chemical staining (iodine, sulfuric acid, and ninhydrin). Pass/fail results are based upon the size and relative elution position of the sample spots compared to reference standards. Another component to the Minilab is disintegration testing (not evaluated in the current study), in which a sample is placed into a standardized solution and the time taken to disintegrate/dissolve is measured. Deviations in the time of tablet disintegration can reveal a potential poor-quality medicine.<br>**The device can operate in the field without a computer.**<br>**Samples are destroyed in the analysis.**
**APIs tested**	All seven APIs/combination of APIs
**Specifications**	*Dimensions:* 52 cm (H) x 83 cm (W) x 29 cm (D)<br>*Weight:* 25 kg<br>*Power source:* 4 AA Batteries for each UV light source
**Cost***	***Capital cost***<br>• Minilab TLC Test Kit unit: ~US$ 2,510<br>TLC Test Kit unit includes manual caliper, laboratory glass, thermometer, spatula and pestle, scissors, blade/scalpel, aluminum foil, funnel, straight pipette, hot plate, test-tube rack, UV-hand lamp and battery, TLC dipping chamber, etc.<br>• Reference standard: ~US$ 270 (for a set of 12 antimalarials)<br>***Recurring costs***<br>• Required solvents and consumable material: ~US$ 6.96 per run
**Reference library considerations**	Preparing the reference standard solutions requires a stock of genuine medicines for every API. Good storage practices and routine stock checks are necessary to ensure the quality of these reference samples. The protocol states to use the entire medicine for preparation. This produces enough reference solution for hundreds of experiments, but the reference solution cannot be used after 2 days as the APIs are more prone to degradation when dissolved. In the laboratory phase of the study, ultraperformance LC (UPLC)-confirmed genuine medicines were used for reference sample preparation.
**Calibration considerations**	None
**Considerations for the present study**	The TLC portion of the Global Pharma Health Fund Minilab was evaluated. There were issues with shipping chemicals to the Georgia Tech laboratory in the USA so the kit was not supplied with reference samples and reagents. Reference samples were derived from medicines in the investigators' stockpile that were confirmed by UPLC to be genuine and the chemicals were sourced from distributors. The Minilab is not a formulation-specific device.
**Testing abilities**	The device verifies label claims on drug identity and content, and detects counterfeit medicines containing the wrong, much too high, much too low or zero levels of active ingredients.<br>Because TLC experiments of the samples tested are run together with 80% and 100% API reference standard solutions, the Minilab TLC methods allow a range of 80 to 100% API, as lower and higher acceptable limits.

* The costs reported here do not include VAT

Table S4 E. Neospectra 2.5 NIR spectrometer overview

**Manufacturer/ Developer**	Si-Ware Systems<br>https://www.neospectra.com/neospectra-module/
**Technology overview**	The Neospectra 2.5 is a near-infrared modular instrument that can be set up to the user's specifications using either components supplied by the manufacturer or components sourced from third parties. The component that Si-ware manufactures contains a Michelson interferometer (an optical module needed to deconvolute the infrared signal) and a detector. The other necessary components provided by Si-Ware for this study were the following: a light source with a high-intensity dongle, a white reference tile, a Thor Lab fiber-optic probe holder, and a Thor Labs fiber-optic cable and sampling probe. With these components, it is possible to test tablets inside their blister packets as well as taken out of their packaging. All the components provided connect to each other with a simple twist lock connection. The Neospectra 2.5 connects to a computer via a USB cable. The computer acts as the software user interface and command module for the detector.<br>**The device cannot operate in the field without a computer.**<br>**Samples are not destroyed during analysis.**
**APIs tested**	All seven APIs/combination of APIs
**Specifications**	*Dimensions:* Neospectra 2.5 unit: 7.9 cm (H) x 5 cm (W) x 2.5 cm (D) ; Light Source : 15cm (H) x 7.8cm (W) x 3.7cm (D) ; Fiber Optic Cable and Probe 0.6 cm (Ø) x 1 m (L)<br>*Weight:* Neospectra 2.5 = 125 g; Light Source = 900 g; Fiber Optic Cable = 100 g; White Reflective Tile = 27.3 g; Probe Holder = 117 g<br>*Spectral range :* 1350 – 2500 nm<br>*Power source:* USB connection for the Neospectra 2.5 unit only. Light source and computer powered from mains electricity<br>*Internal File Storage Size:* Master computer dependent<br>*Library/Data File Size:* Library N/A; Data file size about 13 kB
**Cost***	*Capital cost (sourcing parts individually)*<br>• Neospectra 2.5 Unit: ~US$ 3,000†‡<br>• Light source (Avantes AVALIGHT-HAL-MINI): ~US$ 1,030<br>• White reference Tile (Avantes): ~US$ 310<br>• Fiber-optic cable and probe (Thor Labs FG550LEC-YCABLE-SP)US$ 1261<br>• Probe holder (Thor Labs RPH): ~US$ 67.83<br>• Computer laptop: ~US$ 500<br>*Recurring costs*<br>• No significant cost per run
**Reference library considerations**	As sold, the software for the Neospectra 2.5 does not contain library function capabilities. However, SI-ware offers a software kit to help interface the module with third-party or user-generated software/code. Thus, one could create custom library software specifically designed for medicine quality analysis.
**Calibration considerations**	Prior to analysis, a background scan of the white reference tile must be taken. A wavenumber correction function is also available if there is deviation in the wavenumber and can be done internally automatically or with an external reference sample.
**Method adaptation for the present study**	The sampling probe was set up with a clamp so that the sampling window was parallel to the table. Tablets could then be placed and kept on the probe window without the user having to hold the probe and minimize probe movement. The lack of a library comparison software function meant that the experimental spectra were visually compared to reference spectra by overlaying the experimental and reference spectra in the same computer window. To minimize bias, the first investigator conducted the experiments and a second investigator, who was unaware of the sample identity, evaluated the data. This device is formulation-specific.
**Testing abilities**	Screening falsified medicines is potentially possible for all medicines. With additional analytical software, the instrument should be able to detect significant changes in the concentrations of the active ingredient. Algorithms should be developed for each API to enhance detection.<br>The instrument can test through transparent blisters and glass vials.

* The costs reported here do not include VAT
† Ordering several devices from the manufacture is subject to potential reduced purchase cost
‡ A new model, the Neospectra 2.5 Micro (a lower cost module) has been made available during the current work, but that it has not been tested in this study

Table S4 F. NIR-S-G1 (NIRscan) NIR spectrometer overview

**Manufacturer/ Developer**	InnoSpectra<br>http://www.inno-spectra.com/en/product
**Technology overview**	The NIR-S-G1 consists of two separate devices; a near-infrared sampling unit and a smartphone that runs an Android® based operating system. The near-infrared sampling unit contains all the hardware necessary for sampling the target (light source, sampling window, optics, and detector) and cooperates with the smartphone. The smartphone acts as the unit's user graphical interface, command module for the sampling unit, and data storage for the device. The smartphone application used was "PillScanNIR" that was developed at Global Good at the time of testing. Communication between the sampling unit and smartphone is achieved using Bluetooth® wireless technology.<br>**The device can operate in the field without a computer.**<br>**Samples are not destroyed during analysis.**
**APIs tested**	All seven APIs/combination of APIs
**Specifications**	*Dimensions:* NIR instrument 8 cm (H) x 6 cm (W) x 4 cm (D)<br>Android Phone for data collection 15 cm (H) x 7.5 cm (W) x 0.5 cm (D)<br>*Weight:* 135 grams (NIR unit)<br>*Power source:* Both the NIR unit and smartphone are powered by internal li-ion batteries and can be recharged using the same micro-USB cable<br>*Spectral range*: 900 nm to 1700 nm<br>*Internal File Storage Size:* Master smart phone dependent<br>*Library/Data File Size:* Entire library size for study 73kB; Data file size about 11 kB
**Cost***	***Upfront cost***<br>• One NIR unit: ~US$ 1,199†<br>• Smartphone ~US$ 200<br>***Recurring costs***<br>• NIR unit battery replacement (expected 5-years life): ~US$ 30<br>• Required consumable material: ~US$ 0.04 per run
**Calibration considerations**	The user does not need to or cannot calibrate the device at the time of testing.
**Reference library considerations**	Reference library entries could only be created by the developer of the application (based in the USA) for this project at the time of testing. Genuine samples of the medicine had to be sent to the developer, who, after processing and creating the reference library entry, sent the updated reference library file (from an email or cloud-based server) to the user, who must place it in the correct folder on the smartphone for use. We understand that the developers are implementing a system for users to create reference libraries, but we did not have access to this system. This device is formulation-specific.
**Testing abilities**	Screening falsified medicines is potentially possible for all medicines, provided that formulation-specific reference libraries are available. The current algorithms available in the device have not been developed for substandard medicines detection. Algorithms should be developed on an API-specific basis to enhance detection. The device is able to test through transparent blisters and glass vials with a reference library created using packaged samples.

* The costs reported here do not include VAT
† Ordering several devices from the manufacture is subject to potential reduced purchase cost

Table S4 G. Paper Analytical Devices (PADs) single use device overview.

**Manufacturer/ Developer**	University of Notre-Dame<br>https://padproject.nd.edu/
**Technology overview**	The Paper Analytical Devices (PADs) are a colorimetric test that require water and a spatula-like tool. There are 12 embedded columns on a card that are known as "lanes". Each lane contains a specific colorimetric test that interacts with a particular functional group on a molecule of the tested product. The medicine powder to be tested is applied to a PAD by depositing and compressing a line in the middle of the card with a spatula-like tool, across all the lanes. The base end of the card is then placed into water (ordinary water can be used according to the developer, but deionized water is preferred to limit the chance of interferences), which travels up the card by capillary action to dissolve the reagents. As the dissolved reagents pass through the deposit line, they interact with the API/excipients and the resulting chemical reaction is captured by the appearance or non-appearance of a color in each lane. The final color code that is generated is used to determine if a certain API is present in the sample by comparing the color code to a reference color code.<br>**The device can operate in the field without a computer.**<br>**Samples are destroyed in the analysis.**
**APIs tested**	Amoxicillin, Azithromycin, Piperaquine, Ofloxacin, Sulfamethoxazole
**Specifications**	*Dimensions:* 11 cm (H) x 7 cm (W) x 0.1 cm (D)<br>*Weight:* 1.5 grams<br>*Power source:* None – single use device
**Cost***	• ~ US$ 3 per PAD (per test)<br>• Required popsicle stick, aluminum foil and water: ~ US$ 0.06
**Calibration considerations**	N/A
**Reference library considerations**	A reference photo (API-specific color code) is required. Reading the PAD can currently be done by eye by comparing the experimental card to the reference photo provided by the developer with instructions on how to read the code provided by the developer.<br>There are ongoing efforts by the developers and their partners to develop and test a smartphone application so that the results of the test can be computationally analyzed.
**Considerations for the present study**	The PADs used in this study were experimental cards. They were adapted by the developer (three lanes of chemicals were added to the originally developed PADs), to allow testing of the 5 APIs included in the present study.[†] However, there were no chemical reagents in the lanes that would enable the screening of clavulanic acid and dihydroartemisinin. In addition, the absence of trimethoprim in an SMTM test would not be detected. Trimethoprim has no unique lane of its own and shares the same lanes as sulfamethoxazole, except for one lane that uniquely targets sulfamethoxazole.<br>The PADs were read by comparing with printed reference photographs provided by the developer (printed copies used as reference in the laboratory; onscreen images displayed on smartphone used in the field).<br>The PADs were shipped in sealed foil storage bags with no special requirements and exposed to temperatures from 10 – 40°C during transportation. They were received approximately 2 months before being used and were stored in their original, sealed bags at approximately 4°C prior to testing.
**Testing abilities**	The PADs used were designed to detect the presence of the API (and of some potentially wrong API) but cannot be used to quantitate the amount of API, i.e. they have no ability to detect substandard medicines (both containing low and high API). The device is not formulation-specific.

* The costs reported here do not include VAT

[†] Lane A: DMAC detects anilines and indoles, and Lane I : Napthoquinone sulfonate + acid detects anilines were used to detect sulfamethoxazole; Lane B: Iodoplatinate detects tertiary amines (confirms lanes D and E) was used to detect sulfamethoxazole and trimethoprim.

Table S4 H. PharmaChk microfluidic device overview

**Manufacturer/Developer**	Boston University<br>-No website available-
**Technology overview**	The PharmaChk is a portable microfluidic device designed to quantify the amount of API in a sample. It is based on luminescence chemistry. The system comes in two major components: the experimental apparatus and an external computer. The experimental apparatus is supplied in a hard case and includes syringe pumps, a sampling chamber (or dissolution vessel) with a sonicator, a cartridge containing the microfluidic channels, and the detector. Detection of the API is based on a chemical reaction that causes luminesce when present. Currently, the device is limited to detecting ART. A single detector measures the luminescent light coming from each channel of the device where the references at 100%, 50% and 10% of the correct API concentration are run simultaneously.<br>Three types of solutions must be prepared before analysis: the probe solution, the reference standard solutions, and the tested sample solution. The probe for ART consists of a solution containing hematin, fluorescein, and luminal. The sample solution is prepared by a single extraction of the API. The external computer, which acts as the command module for the PharmaChk, is connected via a USB cable.<br>**The device cannot operate in the field without a computer.**<br>**Samples are destroyed in the analysis.**
**APIs tested**	Artesunate
**Specifications**	*Dimensions:* 50 cm (H) x 42 cm (W) x 21 cm (D)<br>*Weight:* 13.2 kg<br>*Wavelength Detection: 425 nm, 525nm*<br>*Power source:* Mains Electricity<br>*Internal File Storage Size:* Master computer dependent<br>*Library/Data File Size:* Library N/A; Data file size about 17 kB
**Cost**	Unknown (device under development)
**Calibration considerations**	Detectors occasionally need focus adjustment to clearly see all the microfluidic channels for quantitation. An automatic calibration curve is constructed using the 100%, 50% and 10% of the correct API reference standards. No user input required.
**Reference library considerations**	Reference samples at 100%, 50% and 10% of the correct API concentration are needed for the calibration of the device. These can be prepared either using the raw API or medicines containing the right amount of the API of interest.
**Method adaptation for the present study**	The prototype of the PharmaChk was tested by the chemist investigator, who was trained by the developer of the PharmaChk at the developer's laboratory. This work could not be conducted at Georgia Tech because the PharmaChk is still ongoing development and testing and could not be removed from the developer's laboratory. The testing of the samples included in this study was conducted without the intervention of the developer.
**Testing abilities**	Aptamers or other specific reactions to detect each API need to be developed. When the current project started, the device was only able to test artesunate samples among the APIs selected for this study.<br>The developers states that the device can determine %API.<br>PharmaChk is not a formulation-specific device.

Table S4 I. Progeny Raman spectrometer overview.

**Manufacturer/Developer**	Rigaku<br>https://www.rigaku.com/en/progeny
**Technology overview**	The Progeny is a portable Raman instrument that uses a 1064-nm laser as the excitation source to minimize potential sample fluorescence signals. The entire device can be set up and operated using the touchscreen graphical user interface built into the instrument. This includes generating reference libraries and analyzing data. A computer is not required. The Progeny comes with a base that doubles as a charging platform and device holder for easier sampling. The instrument can be powered from the mains or by interchangeable Li-ion battery packs. Data can be exported via USB cable or through Wi-Fi in PDF format to an external computer.<br>**The device can operate in the field without a computer.**<br>**Samples are not destroyed during analysis.**
**APIs tested**	All seven APIs/combination of APIs
**Specifications**	*Dimensions:* 8 cm (H) x 30 cm (W) x 7 cm (D)<br>*Weight:* 1.6 kg<br>*Excitation wavelength: 1064 nm*<br>*Spectral range: 200 to 2500 cm⁻**<br>*Power source:* Li-ion battery<br>*Internal File Storage Size:* 64 GB and expandable by the manufacturer<br>*Library/Data File Size:* Library linked to data file; Data file size about 100 kB each (pdf, xml, txt)
**Cost***	*Capital cost*[†]<br>• One Progeny unit: ~US$ 61,317<br>*Recurring costs*<br>• Cost per run (consumables needed): ~US$ 0.04<br>• Battery replacement (expected 2-years life): ~US$ 290[†]
**Calibration considerations**	Daily calibrations are recommended to ensure device consistency. A calibrant (benzonitrile) is provided by the manufacturer at purchase. After a successful calibration lasting ~30 seconds, the sample can be loaded and is ready for analysis.
**Reference library considerations**	Reference library spectra creation is simple. The user records the spectrum of a good-quality sample is using “Scan Mode”, presses the “create reference library” button, creates a name for the reference library spectra, and adds the spectrum to the appropriate library folder.
**Method adaptation for the present study**	The artesunate powder samples proved to be difficult to analyses because there was little API powder to work with. Due to the power of the laser and bulkiness of the device, the API had to be removed from the glass vial and placed into a small polyethylene bag to accumulate enough powder in as small of area as possible to generate a good, reproducible signal. In the absence of a recommended protocol as to which function to use by the developer to test the quality of a medicine, the “Analyze” function (search for a match in the whole library) was first run and the “Application” function was then run twice (refer to experimental protocol for details on interpretation).
**Testing abilities**	Screening falsified medicines is potentially possible for all medicines, provided that formulation-specific reference libraries are available.<br>The current algorithms available in the device have not been developed for substandard medicines detection. Algorithms should be developed on an API-specific basis to enhance detection.<br>The device can test through transparent blisters and glass vials with reference libraries created using packaged samples.<br>The device is formulation-specific.

* The costs reported here do not include VAT
[†] Cost may vary based on location; Ordering several devices to the manufacturer is subject to potential reduced purchase cost

Table S4 J. Rapid Diagnostic Tests (RDTs) single use device overview.

**Manufacturer/Developer**	Pennsylvania State University<br>-No Website Available-
**Technology overview**	The RDTs are single-use, disposable, and API-specific immunoassay tests. Antibodies interact with the API and result in a red test line when there is insufficient or zero API. The user performs an alcohol extraction of the medicine sample and dilutes the extract with water into low and high concentration samples. For the first run, the user adds 3 drops of the low concentration sample into the well of the RDT cartridge and waits 5 minutes for the RDT to develop. The control line must appear for every experiment or the test is deemed invalid. In the presence of a control line, the **absence** of the red test line deems the test sample to be a good-quality medicine. If the test line appears, the sample must be retested on a new RDT using the higher concentration sample solution. If the red test line is absent in testing with the high concentration solution, the medicine is deemed substandard since the lower concentration sample test failed. If the red test line appears in testing with the high concentration solution, the sample is deemed falsified since the API may not be present. RDTs must be stored in the fridge. Developers state that the shelf-life with correct storage is one year.<br>**The device can operate in the field without a computer.**<br>**Samples are destroyed in the analysis.**
**APIs tested**	Dihydroartemisinin, Artesunate
**Specifications**	*Dimensions:* 7 cm (H) x 2 cm (W) x 0.5 cm (D)<br>*Weight:* 4.1 g<br>*Power source:* None needed
**Cost***	~US$ 2-3 per RDT† Consumables (Alcohol, water)
**Calibration considerations**	None
**Reference library considerations**	None
**Method adaptation for the present study**	Artemether testing results were not included during the study. The positive control experiments conducted using pure stock artemether as well as the UPLC-confirmed genuine Coartem samples were both classified as being poor quality following the RDT protocols.
**Testing abilities**	The device can identify substandard medicines, as stated by the developer, without mention of the upper threshold of %API that can be detected.<br>This is not a formulation-specific device.

* The costs reported here do not include VAT
† Cost estimated by the manufacturer. The device is not marketed yet and is subject to variation. Purchasing several RDTs is subject to potential reduced purchase cost.

Table S4 K. Truscan RM Raman spectrometer overview.

**Manufacturer/ Developer**	Thermo Fisher Scientific<br>https://www.thermofisher.com/order/catalog/product/TRUSCANRM
**Technology overview**	The Truscan RM is a handheld Raman instrument that utilizes a 785-nm laser as the excitation source. The instrument is operated by buttons located below the LCD screen source. Three sampling apparatuses come with the device: a tablet holder, a vial holder, and a sampling cone. The tablet holder holds the sample tablet in an enclosed container with a strong spring to press the tablet flush against the sampling window. Only tablets with thickness < 7mm and able to withstand the force of the spring can be used with the tablet holder. Oral forms that are too thick, in powder form, or in a blister pack are tested with the nose cone, which acts as a spacer for ensuring the correct distance between the sample and the device aperture. To start analysis, samples are either placed in the tablet holder or held flush against the nose cone and are then scanned. The instrument gives a pass/fail result. The data can be exported in a PDF format with a computer to generate reports. To access all the features, including the generation of reference libraries, the TruScan RM must be connected to a Windows computer via a special dongle and ethernet cable (USB cable connection is not possible). The IP addresses on the computer and TruScan RM must be set up and appropriate firewall permissions given to the Truscan RM to communicate with the computer. An additional print-to-PDF software (novaPDF) must be installed on the computer and set to the default printer for the computer, and the Truscan RM sync software package must be downloaded to the computer.<br>**The device can operate in the field without a computer.**<br>**Samples are not destroyed during the analysis.**
**APIs tested**	All seven APIs/combination of APIs
**Specifications**	*Dimensions*: 21 cm (H) x 11 cm (W) x 4 cm (D)<br>*Weight :* 900 grams<br>*Excitation wavelength :* 785 nm<br>*Spectral range :* 250 to 2875 cm-1<br>*Power source:* Li-ion battery<br>*Internal File Storage Size:* Not disclosed<br>*Library/Data File Size:* Up to 10,000 library entries; about 6,000 data scans can be stored in total
**Cost***	*Capital cost*†<br>• Truscan RM unit, Truetools Chemometric Software Package (with Solo by Eigenvector), and Tablet holder: ~US$ 62,500<br>*Recurring costs*<br>• Cost per run (consumables needed): ~US$ 0.04<br>• Battery replacement (expected 2-years life): ~US$ 112†
**Calibration considerations**	A performance check is conducted at least annually (recommended daily) using a polystyrene standard and the vial holder supplied by the manufacturer.
**Reference library considerations**	For reference library creation, the user selects a specific function known as "collecting signatures" (a "signature" is the spectrum of the genuine medicine). Collecting signatures uses the same process as collecting experimental spectra. These signatures are then uploaded from the Truscan RM to the computer. On the computer, the signatures are added to a reference file containing all the information about the sample. All reference files are then uploaded to the Truscan RM to generate a reference library. The user may upload many signatures to the same reference file to introduce variability potentially caused by repositioning or batch effects. All the reference libraries are managed on the computer and then downloaded to the Truscan RM. On the Truscan RM, the appropriate library must be selected and then the instrument is ready to sample.
**Method adaptation for the present study**	The artesunate powder samples proved to be difficult to analyses because there was little API powder to work with. There was a lack of sample to obtain a good signal. The API had to be removed from the glass vial and placed into a small polyethylene bag to accumulate enough powder in as small of area as possible to generate a good, reproducible signal. Although the user may upload many signatures to the same reference library to introduce potential variability caused by repositioning or by batch effects, only one was uploaded per sample to be equivalent to the other Raman instrument (the Progeny) which only can upload one spectrum per library entry.
**Testing abilities**	Screening falsified medicines is potentially possible for all medicines, provided that formulation-specific reference libraries are available. The current algorithms available in the device have not been developed for substandard medicines detection. Algorithms should be developed on an API-specific basis to enhance detection.<br>The device can test through transparent blisters and glass vials with reference library created using packaged samples. This is a formulation-specific device.

* The costs reported here do not include VAT

† Cost may vary based on location; Ordering several devices to the manufacturer is subject to potential reduced purchase cost

Table S4 L. QDa mass spectrometer overview.

**Manufacturer/Developer**	Waters Corporation<br>http://www.waters.com/waters/en_US/ACQUITY-QDa-Mass-Detector-for-Chromatographic-Analysis/nav.htm?cid=134761404&locale=..
**Technology overview**	The QDa is a benchtop mass spectrometer that can detect APIs based on their molecular weights. The basic components, as tested in this study, include a pump, a six-port injector, the mass spectrometer, and a computer for detector control and data recording. Prior to operation of the instrument, the APIs must be extracted and diluted in a solvent that will not interfere with converting the APIs into ions. The diluted API sample is fed through the injector which introduces a precise volume of the sample into a solvent flow that goes toward the electrospray ionization (ESI) source. In the ESI source, the solvents are evaporated and the molecules ionized. These ions then travel into the QDa through the quadrupole region. The quadrupole acts as a mass filter where all unwanted ions are filtered and only ions of interest hit the detector and produce a signal. The two modes of operation for the QDa quadrupole are scanning mode and single-ion recording mode (SIR). In scanning mode, the instrument scans a mass range set by the user. This mass range can be a narrow window of only a couple mass units wide to the instrument's full mass range (30 to 1250 m/z). This scanning function occurs serval times a second to record all the potential ions that enter the QDa. In SIR mode, the user sets the exact ions that will be transmitted by the quadrupole region of the QDa. The instrument then scans through the selected masses to acquire a more sensitive and consistent signal for only the ions of interest (in this study's case, the APIs). At the end of the of experiment, the signal intensities for the API ions of interest in the questioned sample are compared to a calibration curve to quantitate the amount of API in the sample.<br>**The device cannot operate in the field without a computer.**<br>**Samples are destroyed in the analysis.**
**APIs tested**	All seven APIs/combination of APIs are being evaluated
**Specifications**	*Dimensions: 35.3 cm x 20.0 cm x 75.0 cm (including diaphragm pump, but not a six-port injector, solvent pump, and computer)*<br>*Weight:* 29.4 kg (including diaphragm pump, but not a six-port injector, solvent pump, and computer)<br>*Power source:* Mains 110-240 V AC 50/60Hz<br>*Mass range*: 30 to 1250 m/z<br>*Internal File Storage Size:* Master computer dependent<br>*Library/Data File Size:* Library N/A; About
**Cost**	***Capital cost***<br>• Acquity QDa System includes: QDa mass detector, MassLynx Software, Master computer, syringe pump, solution/sample startup kit, 2 ESI probe assemblies, installation certification ~US $76,169.00<br>• Rheodyne Model 7125 Six Port Injector ~US $1,269.00<br>• Waters Reagent Manager ~US $800.00 (used)
**Calibration considerations**	Calibration curves must be generated hourly for every batch of runs. Daily mass and resolution calibrations are recommended, which are conducted by the instrument using an internal calibrant. No sample preparation is required for the mass and resolution calibrations.
**Method adaptation for the present study**	After extraction and dilution of the APIs, the mass spectrometry experiments conducted in this study utilized direct injection methods. The diluted samples were injected into a solvent stream the lead to the ionization source without out any form of chromatographic separation. The detector was kept in SIR mode to boost sensitivity and stability of the API signal.
**Reference Library**	In this study, a reference library was not used to compare the calibration spectra to the questioned sample spectra. A library could be developed for full scan mode analyses to compare a good-quality reference spectrum to the questioned sample spectrum. Each spectrum was compared like a fingerprint because of the potential specific formulations from each manufacturer.
**Testing Abilities**	The instrument can detect changes of >=1-2% relative in the concentration of the API without any software or hardware changes or enhancements. The sensitivity to changes in API can be determined by statistically determining the injection repeatability mean (area under curve for API) and multiply the SD by 3 to determine a discernible change. This instrument is not formulation-specific.
