# Supplementary material for: Laboratory evaluation of twelve portable devices for medicine quality screening
Source: PLoS Negl Trop Dis. 2021 Sep 30;15(9):e0009360. doi: 10.1371/journal.pntd.0009360 (PMC8483346; doi:10.1371/journal.pntd.0009360)
Supplement: S5 Appendix — (PDF) [file pntd.0009360.s005.pdf]

S5 Appendix. Device protocols.

Text S5 A. The 4500a spectrometer operating protocol 2
Fig S5.1. 4500a screenshots of the MicroLab software. 3
Text S5 B. The C-Vue liquid chromatograph operating protocol 8
Fig S5.2. Photos and screenshots of the C-Vue 10
Table S5.1. Experimental parameters for the C-Vue laboratory evaluation 11
Text S5 C. The MicroPHAZIR RX spectrometer operating protocol 12
Fig S5.3. Images of the main screen of the MicroPHAZIR RX 13
Text S5 D. The Minilab TLC kit operating protocol 18
Text S5 E. The Neospectra 2.5 spectrometer operating protocol 19
Fig S5.4. Images and screenshots of the Neospectra 2.5 22
Text S5 F. The NIR-S-G1 spectrometer operating protocol 23
Fig S5.5. Screenshots and images of the NIR-S-G1 24
Text S5 G. PADs operating protocol 25
Fig S5.6. PADs reference library photos. 26
Text S5 H. The PharmaChk microfluidic system operating protocol 27
Text S5 I. The Progeny spectrometer operating protocol 28
Fig S5.7. Screenshots of the Progeny 29
Text S5 J. Rapid Diagnostic Tests operating protocol 33
Table S5.2. Sample Preparations for RDTs 33
Text S5 K. The Truscan RM spectrometer operating protocol 34
Fig S5.8. Screenshots and images of the accessories for the Truscan RM. 34
Text S5 L. The QDa mass spectrometer operating protocol 39
Table S5.3. Table of instrument parameters for the QDa. 42
Table S5.4. Sample preparation methods for the QDa. 43
Fig S5.9. Screenshots and images illustrating QDa operation 44

Text S5 A. The 4500a spectrometer operating protocol

Overview of operating procedures:

The Agilent 4500a Series FTIR consisted of two dependent instruments: the Agilent 4500a FTIR spectrometer and a laptop computer with an installation of the MicroLab software. The software requires a “professional” edition of a Windows operating system, such as Windows XP or newer.

The 4500a contains all the hardware for sampling (e.g. infrared light source, the diamond attenuated total reflectance (ATR) crystal, sample press, and detector), and operates with a laptop computer. The Sony VAIO SVT131A11M, Window 8.1 Pro laptop computer acted as the unit’s user graphical interface as well as the command module for the sampling unit, generating spectra and storing data. A cable connection was used for communication between the sampling unit and the laptop computer.

Basic operation:

Once both units are fully-powered, the user must activate the software “MicroLab PC” on the laptop **(Fig S5.1A)**. From the main screen menu, the user must choose “Methods” **(Fig S5.1B)**. After the appropriate method has been selected, the user can then hit “Start”. The user must then cleans the spectrometer’s sampling window **(Fig S5.1C)**. The system then collects a background spectrum before analysis of each sample. This provides a baseline profile of the system conditions with no sample loaded on the ATR crystal **(Fig S5.1D)**. The user then places the sample onto the sampling window and closes the sample holder, following on-screen instructions **(Fig S5.1E)**. The user then starts sample analysis via the operating window on the laptop screen. The experimental spectrum is displayed together with all the library matches **(Fig S5.1F)**.

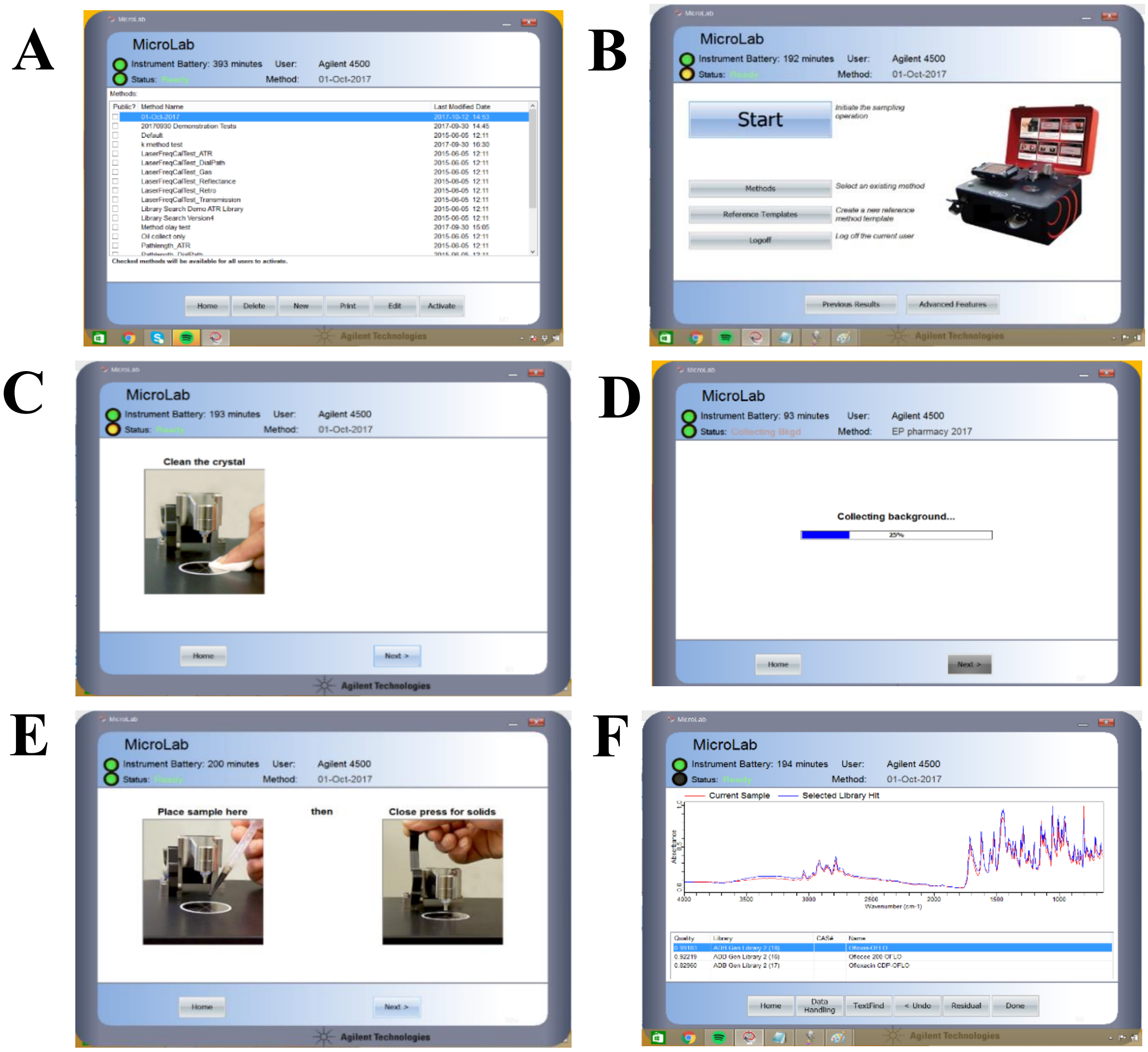

Fig S5.1. 4500a screenshots of the MicroLab software.
(A) Screenshot showing the menu for selecting the spectral reference library. **(B)** Screenshot showing the start menu where the user can begin experiments by hitting “Start”. **(C)** In the first step of the experiment, the software directs the user to clean the sampling interface of the 4500a. **(D)** A screen shot after cleaning, the progress bar indicates the progress of a background scan. **(E)** After the background scan, the software instructs the user to load the sample. **(F)** A screenshot of the results after scanning that include the spectra and the best reference library matches.

Agilent 4500a FTIR reference library features overview:
The 4500a spectrometer allows users to create custom reference libraries by collecting good-quality spectra from medicine samples of known origin and good quality. The collected spectra need to be added to the appropriate library folder within the software (new "libraries" – folders of spectra – can be created by the user). Therefore, the library must kept in the desired library folder where the instrument will search for matches.

Detailed Agilent 4500a FTIR method generation:
The instrument method not only defines how the instrument will conduct its experiments, but also the library that is used for searching for possible matches. Library spectra must be collected with the same method used for collecting spectra from questioned medicine samples in order to ensure that all spectra are comparable.

1. From the main software menu, the user selects "Methods" **(Fig S5.1A).**
2. To generate a new method, select "New" at the bottom of the screen.
    a. To edit a previous method, the user highlights the method to be edited and selects "Edit" at the bottom.
3. The experimental parameters can be altered by the user; however, many of the default parameters work well for pharmaceutical analysis. Ensure the following parameters are set for optimal analysis:
    a. **optional**In the "Type" tab, analysis time can be reduced by selecting "Background Valid time limit" and set the value to 30 minutes so the user does not have to perform a background scan following every sample.
    b. In the "Instrument" tab, for the sampling technology, the user selects from the drop-down menu "ATR".
    c. The "Libraries" tab is where the method is linked to the library chosen (after generating the reference spectra).
        i. The user must remember that new library spectra must be recorded if a new experimental method was developed.
4. After the parameters have been set, the user selects "Save As" to save and name the method. The user should select an easily recognizable file name.

For Agilent 4500a FTIR library generation, the user has to:
1. To generate the library from the main menu by selecting "Advanced Features".
2. Select "Library Management".
3. To generate a new library, select "New Library".
    a. To edit previous libraries, highlight the library name in the scroll down menu.
4. When selecting "New Library", a window should pop up. The library has to be given a name in the "Library Name" line with the box checked for "ATR Spectra". Select "Create" when ready.
    a. To access this menu on old libraries, highlight the library and select "Properties".
5. Ensure the library for editing is highlighted in the scroll down menu and select "Add to Library" to add spectra in the library. To remove spectra, highlight the spectra in the spectra scroll down menu and then select "Delete From Lib".

For Agilent 4500a FTIR library spectra generation, the user has to:
1. In order to select the method with which to make the library spectra, select "Methods" from the main menu.
2. Highlight the method to use, then select "Activate".
    a. When back in the main menu, ensure the correct method is selected by looking at the top of the window next to "Method:".
3. Select "Start" from the main menu **(Fig S5.1B).**
4. Clean the ATR sampling crystal thoroughly with a wipe and solvent before clicking "Next", the instrument will then do a crystal check and then a background check. **(Fig S5.1C,D)**

5. Load the sample on the sample window and apply pressure with the sample press **the press should be finger tight, do not overtighten**, then click "Next" **(Fig S5.1E).**
    a. **Overtightening can damage the instrument
6. Type in the name of the sample in the "(optional) Sample ID" line
    a. Although the line states "(optional)", it is good practice and highly recommended to give any sample recorded a unique name.
    b. Include at least this information in the following order with an underscore "_" in between each:
        i. YearMonthDate (Ex. 20170927).
        ii. Brand Name.
        iii. Active Ingredient(s).
        iv. Batch Number.
        v. ***Optional*** User Initials to determine who made the library.
7. Feel free to type in any relevant information in the "(optional) Comments:" section.
8. In the real-time spectra window below the comment section, look for peaks.
    a. If the spectrum is completely flat, carefully tighten the press until the signal stops increasing ***finger tight only***.
    b. See troubleshooting guide if you still see no signal.
9. Select "Next" when ready.
10. After the spectrum for the sample is measured/recorded, **(Fig S5.1F).**
    a. Select "Details" to look at the acquired spectrum to ensure quality.
    b. Select "Data Handling", then "Add to Library…" to add the spectrum to a library that was generated previously.
        i. Change the "Match Text:" to a desired name when conducting questioned sample analysis.
            1. Include at least this information in the following order with a "_" in between each:
                a. YearMonthDate (Ex. 20170927).
                b. Brand Name.
                c. Active Ingredient(s).
                d. Batch Number.
                e. ***Optional*** User Initials to determine who made the spectra.
        ii. Select the library to add the spectra to by either selecting from the "Select from Method Libraries" or by clicking the "…" button after the "Library Name:" line.
        iii. Select "Add to Library" when done.
    c. Select "Done" to continue with experiments.
    d. Select "Home" to return to the main menu.
11. Add previously collected spectra by accessing the "Previous Results" from the main menu, selecting the spectra you would like to use, and then following the same steps as above.

<u>Agilent 4500a FTIR library files location on the Sony PC:</u>
Library location is C:\Users\Public\Documents\Agilent\Microlab\Libraries.

- Simply copy and paste when the user needs to upload or download libraries.

<u>For Agilent 4500a FTIR calibration, the user need to:</u>
All calibration experiments are performed from the "Systems Check Menu" located in the "Advanced Features" menu in the main menu.

- Weekly/Daily Calibration.
    1. Ensure the "Performance (Signal-to-noise)" is selected, and only this test,
        - Also set the "Number of Tests:" to 5 so speed up analysis.
    2. Select "Next", Follow the on-screen instructions.
        - The user does not need any sample for this calibration procedure.

    3. After the test is complete, select the “Laser Frequency Calibration Check”, and only this test.
        - Set the “Number of Runs:” to 5 and ensure that the “Check value only” box is selected.
    4. Select “Next”, follow the on-screen instructions.
        - The user will need the polystyrene card that comes in the box with the instrument to perform this test.
    5. After these tests, the user is ready for performing experiments.
- Monthly (or at least several times a year):
    1. Ensure all the possible tests are selected.
        - If the user does not have access to toluene, do not perform the “Pathlength Calibration Check”.
        - Ensure all the number of tests, minutes, and runs are set to their default values:
            - 15 tests
            - 30 minutes
            - 15 runs
            - 5 runs
    2. Select “Next”, follow the on-screen instructions.
        - In rare occasions, we have observed situations where the instrument would not proceed to the next calibration stage after the first test. If this happens, the user simply has to re-select the calibration tests that were not performed and continue.

For troubleshooting protocol, the user needs to:

1. Ensure that the crystal was clean during the background scan.
    a. Although there is a crystal clean check, do not assume it is perfect and clean with tissues and solvent (isopropanol is ideal).
2. Ensure there is enough powdered sample and pressure on the ATR diamond crystal.
    a. Release the sample press and transfer more powder onto the sample window if necessary.
    b. Ensure the sample press is finger-tight, do not be afraid to add a little pressure, but monitor the real-time signal (this is achieved in the software window where you also type in the sample name and comments before recording the spectra to save).
3. Ensure the battery is more than 1/3 charged (just in case it is a power issue), or that the instrument is connected to the power outlet.
4. When in doubt, restart both the device and the computer.
    a. After restart, ensure that before you open the “MicroLab PC” software, the FTIR spectrometer is turned on and securely plugged into the computer or else the software will not start.
5. Conduct the weekly/daily calibration procedure to ensure the errors observed are not due to an instrument problem.
    a. If the weekly/daily calibration passes, most likely errors or problems are due to a sampling issue.
6. If an issue with the clean check is suspected, the user can reset the check as follows:
    a. From the main menu, select “Advanced Features” and then select “Diagnostics.
    b. To reset what the instrument thinks is a clean ATR window, select “Reset Clean Validity”.
        i. ***Ensure the sampling crystal and press are as clean as possible***
7. If the weekly/daily calibration FAILS, the user performs a monthly calibration.
8. If the monthly calibration FAILS, the user restarts the device and computer.
    a. The user may want to unplug the instrument from the mains and wait for the battery to discharge to simulate removing the battery.
9. Perform another daily calibration, then monthly calibration.

10. If it fails again, contact technical support.
    a. From the main menu, select “Advanced Features” and then select “Diagnostics”.
    b. The information found in this window may be relevant to understanding the cause of the problem.

<u>Downloading the MicroLab PC software to a computer:</u>

- The software download is no more difficult than any other program; however, the software is typically can only be registered to one or a handful of computers. Only these computers will have full software functionality.

Text S5 B. The C-Vue liquid chromatograph operating protocol

Sample preparation:

1. Tablets were pulverized by wrapping the sample in weighing paper and crushing it with a pestle. Samples that were already in powder form (e.g. ART) were not processed any further.
2. A sample of the medicinal powder (between 10-25mg) was placed into a clean vial and extracted using 5 mL of an extraction solution.
    a. Sulfamethoxazole/Trimethoprim – methanol.
    b. Amoxicillin/Clavulanic Acid – methanol.
    c. Ofloxacin – 4.9 mL of water: 0.1 mL of glacial acetic acid.
3. Based on the amounts weighed and the volume of extraction solvent, the %API concentration was calculated based on the reported values issued by the manufacturer.
4. These extracts were then diluted to the working concentration required for the C-Vue using the mobile phase utilized in each API-specific LC protocol.
    a. SMTM- 100 ppm SM and 20 ppm TM.
        i. In 30:70 methanol: water with 0.1M disodium phosphate.
    b. ACA - 100 ppm for amoxicillin resulting with either
        1. For a formulation of ACA that contains 875 mg of A, prepare 14 ppm of CA
        2. For a formulation of ACA that contains 500 mg of A, prepare 25 ppm CA
        ii. in 5:95 methanol: water with 0.1M disodium phosphate.
    c. OFLO – 100 ppm OFLO.
        i. In 30:70 methanol: water with 0.1 M disodium phosphate.
5. Samples were stored in a 4°C refrigerator when not in use.

C-Vue calibration curve preparation:

1. Extracts and dilutions were treated identically, following steps 1 through 4 in the previous section, with the following exceptions:
    a. API calibration solutions were derived from a pure API stock solution using standards from TCI Chemical or Sigma Aldrich.
    b. A range of calibration solutions were prepared in which one or both APIs were present, as follows:
        i. SMTM - 30/6 ppm, 60/12 ppm, 90/18 ppm, 120/25 ppm.
        ii. ACA – 30/4 ppm, 60/15 ppm, 90/25 ppm, 120/35 ppm.
        iii. OFLO- 30 ppm, 60 ppm, 90 ppm, 120 ppm.

For preparing the C-Vue chromatograph for analysis, the user has to:

1. When starting with a dry pump, or when switching mobile phases, ensure the column is disconnected from the six-port injection valve.
2. Fill the 60-mL mobile phase syringe with the desired mobile phase solution **(Fig S5.2A)**
    a. To prevent gas bubbles from entering the LC system, connect the syringe filled with mobile phase liquid to an empty 60 mL syringe.
    b. Secure the mobile phase syringe’s plunger to prevent movement.
    c. Pull on the plunger of the empty syringe to generate a vacuum within both syringes until the empty syringe’s plunger reaches its maximum distance before removing the plunger from the syringe.
    d. Tap the mobile phase syringe until the bubbles in the solution subside.
    e. Release the vacuum and remove the mobile phase syringe and push out any remaining air in the syringe.
3. Load the mobile phase syringe into the C-Vue.
4. Purge the injector by adding some pressure to the pump spring.
5. Capture the mobile phase coming out of the injector in a container, until no air bubbles are observed.
6. Connect the injector and the column together.

7. Connect the column to the detector(s) and connect the lines from the detector(s) to a waste container.
8. Flow mobile phase through the LC system at a pump spring compression of 30 mm for about 30 min.
9. At the same time, turn the detectors on to allow them to warm-up.
   a. The detectors are warmed up when a steady signal is observed in the C-Vue software.
10. During experiments, ensure the pump spring is set to 50 mm prior to injection and recording.

<u>For C-Vue sample/calibration injection protocol, the user needs to:</u>

1. Ensure the six-port injector is in the load position **(Fig S5.2B).**
2. Using a 1-mL syringe, flow 1 mL of a blank mobile phase to clean the injector.
3. After cleaning, fill a 1-mL syringe with at least 0.8 mL of sample solution.
4. Flow 0.5 mL of sample solution through the syringe to clean the injector and subsequently the 0.3 mL to load the sample.
   a. For subsequent injections of the sample, the 0.5 mL injection does not need to be repeated.
5. To inject the sample into the system, the injector should be rapidly switched to the "Inject" position at the same time as hitting "Run" (this will begin recording data, see steps 5 and 6 in section "C-Vue Software Set-up and Recording" to stop recording) in the C-Vue software and remain there for the duration of the run. **(Fig S5.2C).**
6. To load the next sample, switch the injector back to the "Load" position and repeat steps 2 through 5 for different samples, or 4 through 5 if repeating the same sample.

<u>For C-Vue software set-up and recording, the user needs to:</u>

1. Turn on the computer.
2. Start the C-Vue software.
3. Ensure signal appears in the C-Vue software window.
4. Follow steps 4 through 6 in the "C-Vue Sample/Calibration Injection Protocol".
5. After recording the necessary data, click "STOP" to stop recording.
6. The File Explorer window should pop up, save the file to a designated folder with a filename.

<u>For C-Vue software data analysis, the user has to:</u>

1. In the "Analysis" tab of the software, press the open button.
2. Select "Raw Data .csv".
3. Open the file that was saved.
4. Integrate the peaks **(Fig S5.2D).**
   a. Press the "Manual Integrate button".
   b. From left side to the right side of the chromatogram, right click the beginning and end of the peak.
      i. For multiple peaks, continue marking the beginning and end of each peak
   c. Once all the peaks have been marked, left click.
5. Collect the Area information to compile calibration curves through linear regression analysis and determine the concentration of the sample tested.

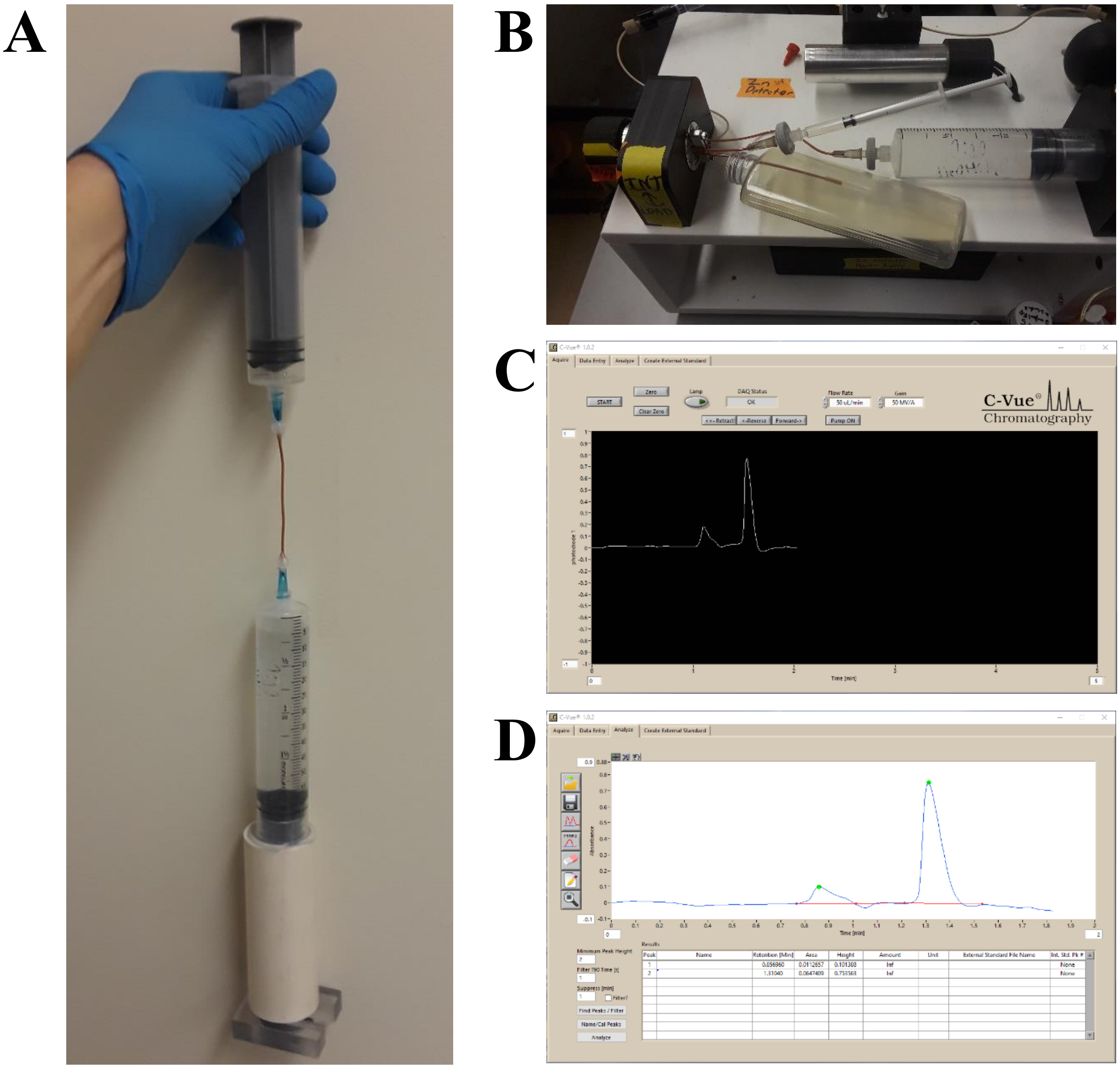

Fig S5.2. Photos and screenshots of the C-Vue
(A) Image of the degassing set-up for the mobile phase solvent. This removes dissolved gases in the solvent, preventing gas bubbles from entering the system that can cause false peaks. Briefly, the mobile phase solvent is loaded in the bottom syringe. Afterwards, the top syringe is attached and the plunger is pulled on the top syringe to generate a vacuum. Under vacuum, the bottom solvent filled syringe is tapped a few times to get rid of the dissolved gases. **(B)** Image of the six-port injector set-up with the sample injection syringe (1-mL syringe), the mobile phase syringe (50-mL syringe), and injector overflow catch bottle. Behind the injector is the column and the silver tube is the zinc lamp for one of the detectors. **(C)** Screenshot of a chromatogram being collected. **(D)** Screenshot of the chromatogram being processed to determine the area under the curve of the peak.

Table S5.1. Experimental parameters for the C-Vue laboratory evaluation

Active Ingredient	***Ofloxacin***	***Sulfamethoxazole/ Trimethoprim***	***Amoxicillin/Clavulanic Acid***
Mobile Phase*	30:70 Methanol:Water	30:70 Methanol:Water	5:95 Methanol:Water
Pump Spring Load at start (mm)	50	50	50
Retention Time (min)	4.8	4.6/3.5	4.4/3.1
API Extraction Solvent	98:2 Water: Glacial Acetic Acid	Methanol	Methanol
Dilution Solvent*	30:70 Methanol:Water	30:70 Methanol:Water	5:95 Methanol:Water
Final Sample API Target Concentration (ppm)	100	100/20	1.) 100/14.3 for 875/125 mg ACA formulation<br>2.) 100/25.2 for 500 mg/125 mg ACA formulations
Target Calibration Curve Standard Concentrations (ppm)	30<br>60<br>90<br>120	30/6<br>60/12<br>90/18<br>120/24	30/5<br>60/15<br>90/25<br>120/35

* Solvent contains 0.1 M disodium phosphate as a buffer

Text S5 C. The MicroPHAZIR RX spectrometer operating protocol

<u>Overview of the operating procedures:</u>
After turning on the device **(Fig S5.3A)**, the user logs in his/her credentials. The tungsten lamp (infrared source for the device) must then warm up and go through a self-test (run-time ~10 minutes, performed daily) to ensure optimal performance. A more rigorous calibration is required annually with reference samples specifically purchased from Thermo-Fisher Scientific (see below). After a successful self-test, the user goes to the "Run" function, and from the Methods tab, should select the comparator library spectrum for the sample they wish to test. The user is then prompted to input the filename and batch number of the sample.

Three sampling techniques were employed:

a. For tablets tested out of blister packaging, the MicroPHAZIR RX is placed on a table surface so that the sampling window is parallel to the table. The tablets are then rested on the sampling window **(Fig S5.3B)**. If tablets were smaller than the sampling window, a cover was placed on top made out the MicroPHAZIR RX's sample holder **(Fig S5.3C)**.
b. For tablets tested inside the blister packaging, the MicroPHAZIR RX would be held in one of the user's hands and the other hand would hold the blister pack sample flush against the sampling window **(Fig S5.3D)**.
c. For the ART samples (API powder in vial), the artesunate vial would first be tapped several times against a hard surface to shake the powder to the bottom of the vial. The vial was then placed on its base on top of the sampling window.

Once the sample was appropriately positioned, the user either pulls the device trigger or presses "go" on the device screen, starting the sample scan. After the scan is complete, a "Pass" or "Fail" results is displayed (see Quick guide below).

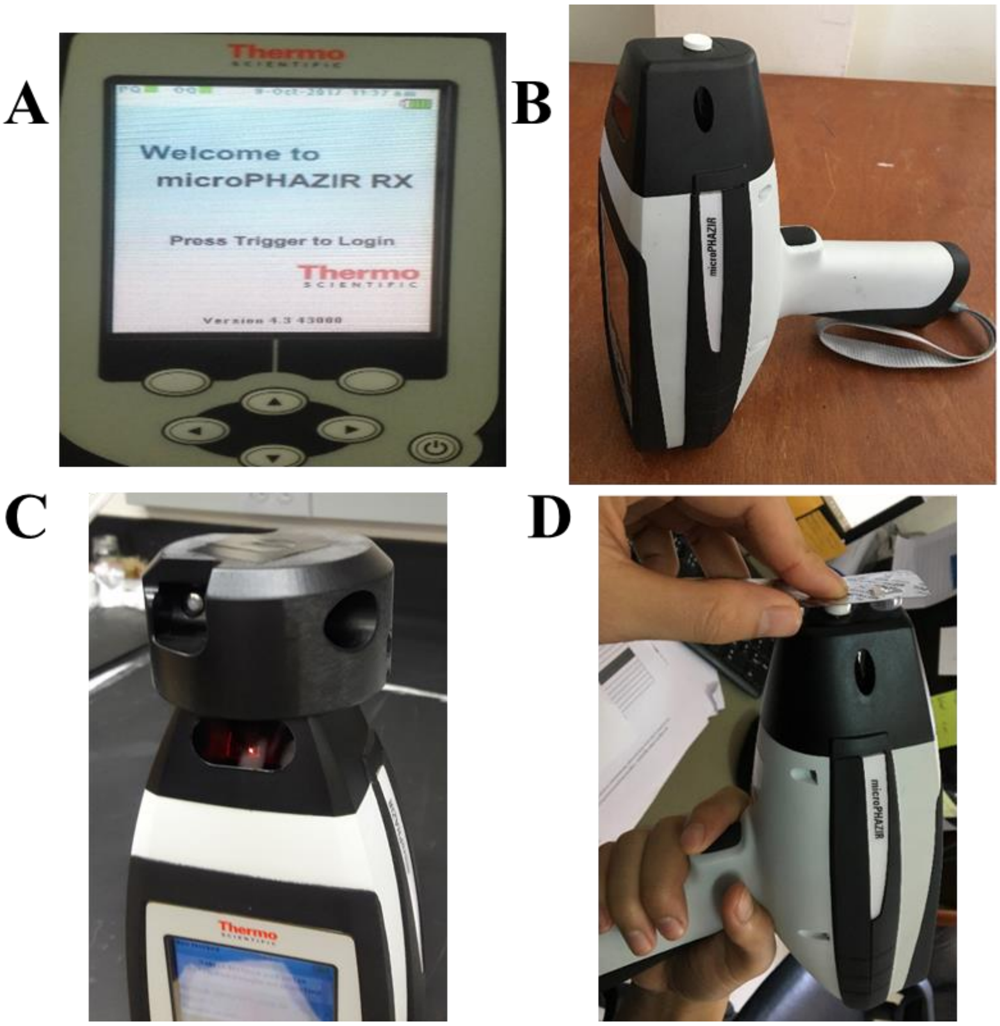

Fig S5.3. Images of the main screen of the MicroPHAZIR RX
(A) Image of the main screen of the MicroPHAZIR RX after it is turned on. **(B)** Image of a tablet that is larger than the sampling interface being scanned by the MicropPHAZIR RX. **(C)** If the tablet is smaller than the sampling interface, a cover constructed out of the standard holder was placed on top of the MicroPHAZIR RX to prevent ambient light from entering the detector. The modification to the standard holder was some electrical tape placed on the top hole. **(D)** Image of a tablet being scanned through transparent blister packaging.

MicroPHAZIR RX reference library features overview:
When generating reference library spectra, the MicroPHAZIR RX guides the user to collect five spectra of the same sample. This step allows the user to introduce some variability into reference collection, like batch variation or sample position to have an average spectrum for comparison. The collected spectra are labelled “signatures”. After spectra collection, the data must be uploaded to a computer for processing. On the computer, all the signatures desired in a library must be uploaded to the same workspace. The user selects the mathematical functions desired, and the software then outputs a single library file that contains all the selected spectra to be uploaded to the MicroPHAZIR RX. Libraries that are generated by the software cannot be edited afterward, thus new libraries must be generated for minor or major edits. Communication between the MicroPHAZIR RX and computer requires downloading two software packages to the computer. When the user is ready to connect the instrument to the computer (via USB cable), the user must activate the sync function on the MicroPHAZIR RX. There are two separate sync functions for uploading experimental data and uploading/downloading reference signatures and libraries.

For MicroPHAZIR RX methods (generating signatures), the user needs to:

1. From the main menu, select “Tools”.
2. Select “Acquire Signature”.
3. Highlight and select “Method:”.
4. Scroll up and then to the left to highlight the “New” tab.
5. Press select to add a new signature entry to the device.
6. Type in the method file name.
   a. Include at least this information in the following order with a dash “-” in between each:
      i. YearMonthDate (Ex. 20170927).
      ii. Brand Name.
      iii. Active Ingredient(s).
      iv. Batch Number.
      v. ***Optional*** User Initials to determine who made the library.
7. Press “Done”.
8. Highlight and select “Batch:”.
9. Scroll up and then to the left to highlight the “New” tab.
   a. If the user is taking another signature of the same batch, scroll through the options to find the correct batch number, skip the next steps until the user gets to the sample number.
10. Press select to add a new signature entry to the device.
11. Type in the batch number of the sample.
12. Press “Done”.
13. The “Sample:” line should autofill,
    a. If not, highlight and repeat the same steps as creating a new batch or selecting an old one.
14. Once that the samples information is confirmed to be correct, highlight and select “Continue to Start Run”.
15. Follow the on screen instructions:
    a. Place the sample on the sampling window (or vice versa).
       i. If the tablets are smaller than the sampling window, place cover over the sample and sampling interface to prevent ambient light from entering the detector .
    b. Pull the device trigger or press “Select” to scan.
       i. Look at the resulting spectra.
          1. If the spectrum looks good (not completely flat, but with broad peaks and valleys), continue to the next scan.
          2. If the spectrum looks poor, press “Esc” to repeat the experiment.

c. Continue until the user has 5 spectra that overlap each other well.

16. If the resulting spectra overlaps well, highlight and select “Save Current Signature and Continue”.
    a. If poor overlap, select “Cancel without Saving” and repeat the experiment.
17. Repeat the process to add more signatures to the library.

<u>For MicroPHAZIR RX uploading the signatures to the computer, the user must:</u>
In this study, the software to process signatures to make libraries was uploaded to a desktop computer.

1. From the main menu, select “Sync”.
2. Connect a mini-USB to the MicroPHAZIR RX, and the USB end to the computer.
    i. **Uploads and downloads from the device and computer take some time, be patient**.
3. If file syncing was successful, a message from the computer should pop-up.
    a. To check if data and signatures were uploaded successfully.
        i. Go to the computers file explorer.
        ii. Access the “Local Disk (C:)”.
        iii. Access the “Thermo” folder.
        iv. Access the “data” folder.
        v. Access the “Archive” folder.
        vi. Look for the folder with the day the device was synced and access it.
        vii. Ensure the data you collected is in that folder.
4. Access the “Method Generator” desktop app.
    a. To ensure full software capabilities, click on the “Model” tab and ensure the “Advanced Modeling” is enabled.
5. To access the data, click on the “File” tab, click “Open Data File” and use the file explorer to find the signatures.
    a. Access the “Local Disk (C:)”.
    b. Access the “Thermo” folder.
    c. Access the “data” folder.
    d. Access the “Archive” folder.
    e. Look for the folder with the day the device was synced and access it.
    f. Access the “Data” folder on the time stamped folder.
    g. Select the signature file that was created the day they were acquired.

Note the user can process multiple different spectra at the same time. Ensure that the software can recognize the samples between different sample acquisitions.

6. In the list adjacent to the “1. Read Data File” button highlighted in green, ensure that the signatures the user would like to make a method with are highlighted in blue.
7. Once selection is highlighted, click the “2. Pre-Process” button highlighted in green.
    a. The spectral window should change if below the pre-process: step 1 was “S. Golay”, step 2 was “Normalize Range”, and the rest were skips (these are the default processing standards).
8. Once selection is highlighted, click the “3. Spectral-Match-KNN” button highlighted in green.
    a. The window below this button should show results indicating how well different spectra can distinguish themselves from one another.
    b. If samples cannot correctly identify themselves,
        i. Either isolate out the bad spectra in near the “1. Read Data File” button highlighted in green.
        ii. **ADVANCED** Attempt to adjust the pre-processing parameters.
        iii. This step can be overridden by continuing onto the next step **be aware** that the library searches may not give the correct result because it may identify the mismatched spectra.
9. Once satisfied with the results from “3. Spectral-Match-KNN” button highlighted in green, press the “4. Creating SM-KNN Application” button highlighted in green.
10. A window should pop up.

    a. Ensure that the experiment is set to “Verification (pass/fail).
    b. All the other parameters are default and work well.
11. Click ”OK”.
12. Save and name the method file in the following folder:
    a. Local Disk (C:)/Thermo/Data/Method Generator/Applications.
        i. Include at least this information in the following order with a dash “-” in between each:
            1. YearMonthDate (Ex. 20170927).
            2. Brand Name.
            3. Active Ingredient(s).
            4. Batch Number .
            5. ***Optional*** User Initials to determine who made the library.
13. Remove the data USB cable from the MicroPHAZIR RX if you have not done so already.

<u>For MicroPHAZIR RX uploading the methods, the user needs to:</u>

1. From the MicroPHAZIR RX main menu, select “Tools”.
2. Select “Configure MicroPHAZIR”.
3. Select “Configure with Libraries”.
4. Connect the USB to the MicroPHAZIR RX and the computer.
5. Access the “MicroPHAZIR Admin” desktop app on the computer.
    a. **Uploads, downloads, and opening the software for the device and computer take some time, be patient**.
    b. Don’t be afraid to click on the desktop app a few times.
6. The methods the user created should be in the right-hand column under the “Local PC Libraries and Methods”.
    a. This is in the Methods “tab” just in case the software opens to the wrong tab.
7. Highlight the method you would like to add to the MicroPHAZIR RX and press the “Copy to Active” button.
8. Once uploaded, close the software and remove the USB cable and the device should be ready for analysis.

<u>For MicroPHAZIR RX calibration, the user must:</u>

- Daily/Weekly Calibration (Self Test).
    1. From the main menu, select “Self Test”.
    2. Select “Start Scan”.
        - If a lamp warm-up warning appears, wait the allotted time for proper warm up.
    3. The device should automatically start its own test.
    4. The result of the performance qualification should pop up.
        - If pass, select “Return to Main Screen” and continue with experimentation.
        - If fails, see troubleshooting protocol.

- Monthly Calibration (Calibrate Reference)
    5. From the main menu, select “Tools”.
    6. Select “Configure MicroPHAZIR”.
    7. Select “Calibrate Reference”.
    8. Place the sample holder on top of the sample interface (Part 810-0139).
        - It’s the black piece of plastic was used as a tablet cover, remove the electrical tape.
    9. Place the “MicroPHAZIR Reference Standard” (barcode 990200017088) in the sample holder.
        - Have the blank white side of the of the reference standard flush with the sample interface.

- The user should see the "WR" side of the reference standard stick out of the sample holder.

10. Press "Select" to start the calibration.
11. The results of the experiment should pop up.
    - If successful, press "OK" and continue with experiments.
    - If failure, follow troubleshooting protocol.

<u>For the MicroPHAZIR RX troubleshooting protocol, the user must:</u>

1. Ensure the sample is properly secured or being held in front of the sampling interface or vial holder.
   a. Minimize and potential ambient light from entering the device.
   b. Hold the sample in one position and do not move.
2. Ensure the battery is more than 1/3 charged (just in case it is a power issue).
3. When in doubt, restart the device.
   a. Remove the battery after shutdown to ensure the device completely turns off.
4. Conduct a Self-Test to ensure it's not an instrument problem.
   a. If the self-test passes, most likely it is a sampling issue.
5. If the self-test FAILS, perform a Calibrate Reference.
6. If the calibrate reference FAILS, restart the device and remove the battery.
   a. Allow the instrument to sit without the battery for 5 minutes to ensure no charge remains in the instrument.
7. Perform another calibrate reference.
8. If it fails again, contact technical support.
   a. May need to do an "Operational Qualification" test (In the "Tools" menu), but requires the reference standards recommended by Thermo that were not included.
   b. In the meantime:
      i. In the "Configure MicroPHAZIR" menu (link found in the "Tools" menu", select "Get Diagnostics".
   a. Connect the Connect the USB to the MicroPHAZIR RX and the computer.
   b. Access the "MicroPHAZIR Admin" desktop app on the computer.
      i. **Uploads, downloads, and opening the software for the device and computer take some time, be patient**.
      ii. Do not be afraid to click on the desktop app a few times.
      iii. The diagnostic data could be critical to determine the source of the error.

<u>MicroPHAZIR RX downloading the software to a computer:</u>

- The software downloads are no more difficult than any other program (lots of clicking "Next" and Accept"); however, the user needs to ensure that both the "Method Generator" and "MicroPHAZIR Admin" software packages are downloaded for full capabilities to be available prior to experimentation.

Text S5 D. The Minilab TLC kit operating protocol

API specific step by step protocols for the Minilab are provided by the Global Pharma Health Fund when a kit is purchased. The protocols are also available online at the Global Pharma Health Fund website. The website link to the protocols is listed below.

https://www.gphf.org/en/minilab/manuals.htm

Text S5 E. The Neospectra 2.5 spectrometer operating protocol

<u>For Neospectra 2.5 assembly/disassembly of the device, the user needs to:</u>

1. For the light source, remove the AVALIGHT-HALM-MINI from its box along with the power supply and the dongle labeled with the word “HIGH” **(Fig S5.4A).**
    a. Attach the power supply to the mains electricity and then the power supply to the light source.
    b. Attach the dongle to the back of the light source and secure it with the screws embedded in the dongle.
    c. Remove the cap on the light output of the source, but do not turn on the light source at this step.
2. For the Neospectra 2.5 detector unit, remove the detector and USB cable from the box. **(Fig S5.4B)**
    a. Attach the USB cable from the computer to the detector unit.
    b. Remove cap from the light input for the detector.
3. For the fiber optic cable and probe, remove from it from the box. ***NOTE: Although the fiber optic cable is flexible, the user can bend the cable too far and break the internal optical fiber and make the cable unusable. Be careful and allow the cable to freely move and bend, never force the cable into a position. ***
    a. Remove the cap from the end of the cable that has a flower pattern of five dots printed on it (this will be the smallest screw connection of the two on the cable) Screw this end into the light source.
    b. Remove the cap from the end of the cable that has the “THORLABS” logo on it (this will be the largest screw connection of the two on the cable). Screw this end into the Neospectra 2.5 detector unit.
    c. Depending on how the user will conduct experiments on medicine tablets and powders, the user can set up the probe in two different ways:
        i. The user can use the probe without any further modification from the above-mentioned set-up. The probe can freely move around and be used like a pen to the surface of the samples is question.
        ii. The user can attach the end of the probe to a probe holder in order to not hold by hand either the sample or probe while conducting experiments (the way the instrument was tested in this study). **(Fig S5.4C)**
            1. Insert the probe in the probe holder until the end of the probe is flush with the largest flat side of the probe holder.
            2. Tighten the set screw to secure the probe in place.
            3. Attach the probe holder to a clamp. Allow sampling window of the probe and flat face of the probe holder be parallel to the floor and face them towards the ceiling. This allows the user to place sample on top of the probe to sample without having to hold it by hand.
4. To disassemble the device, follow the protocol in reverse order.

<u>For the Neospectra 2.5 software download (to install on computer), the user must:</u>

1. Included with the Neospectra 2.5 detector is a flash drive that contains the software that controls the detector.
2. Access the flash drive and open the “SpectroMOST6.1_Setup” file. Follow the onscreen instructions to install.
3. Towards the end of the software installation, the software will inform the user to connect the detector to the computer to finish the configuration.

<u>For Neospectra 2.5 device operational protocol, the user needs to:</u>

1. Turn on the light source and allow the source to warm up for 10 to 15 minutes so the lamp is outputting a consistent amount of light.
2. While the lamp in the light source is warming up, connect the Neospectra to the computer if not done so already.

i. Open the "SpectroMOST" software on the computer.
ii. At the bottom of the window, the "Status" should eventually become green with "NeoSpectra module is Ready!" to ensure the detector is communicating with the master computer.
iii. The detector settings can also be set-up including the "Scan Time", "Resolution", and "Optical Gain Settings". For this study:
    1. Scan Time = 10 s
    2. Resolution = 8 nm @ 1550 nm
    3. Optical Gain Setting = Reflection

3. Once the lamp is warm, the instrument can undergo further set-up.
    i. Remove the cover from the white reference tile and place the white side of the sample flush against the sampling window of the sampling probe.
    ii. In the software, then click "Background" to acquire a background scan to tune the device.
4. The device should be ready to begin scanning samples.
    i. Clean the sampling window with a delicate task wipe and if necessary, some isopropanol if the probe is especially dirty.
    ii. Place the sample in question flush against the sampling window.
        1. For powdered samples, ensure that there is as thick of accumulation of the powder over the sampling window to get the best results.
    iii. When the sample is ready, hit the "Scan" button in the software window **(Fig S5.4D)**.
    iv. If the quality of the spectra looks acceptable, press the "Save" button in the software window to save it in the user's desired folder. ***NOTES:
        1. The spectrum does not autosave after scanning unless the "Auto-save" is checked and enabled.
        2. Ever subsequent scan will overlay with the previous scan(s). When saving, each spectrum will be saved with a single filename set by user and then automatically numbered in order from the first to last scan.
            a. To clear the spectrum window, click "Clear" button.

<u>Neospectra 2.5 data analysis in this study:</u>

1. Samples that were used for reference spectra were tested in the above-mentioned protocol and saved in a specified reference folder. The filenames also stated that the samples were reference spectra.
2. The questioned samples are scanned as stated in the above-mentioned protocol and saved in a separate sample scan folder.
3. The reference spectra were then overlaid on top of the questioned sample spectra.
    a. With the questioned sample spectra in the window, click the "Load" button in the software window.
    b. Open the reference file spectra for the sample in question.
    c. The reference spectra will be loaded into the window as subsequent scans.
    d. Additional sample spectra can be added to window following the same process as with the reference spectra.
4. The data then can be directly analyzed and compared in that window, or a screenshot of the window can be used to export the spectral analysis for a blinded analyst to compare.
5. If the user would like to export the raw spectral data for third party data processing software, the spectra files saved by the SpectroMOST software can be opened in basic text file format (e.g. Notepad) or spreadsheet software (e.g. Excel).

<u>For Neospectra 2.5 calibration, the user must:</u>

1. At a minimum, conduct a new background test with the white reference tile daily. The more often the better to minimize any chance of detector drift that may happen.
2. Monthly, run a "Wavelength & Wavenumber Correction" test, which is recommended. See step 8 in "Neospectra 2.5 Troubleshooting" for how to conduct the correction.

For Neospectra 2.5 troubleshooting, the user needs to:

1. Ensure the detector is connected to the computer
    i. See if the status of the detector is ready at the bottom of the software window.
    ii. If not, unplug the USB cable and plug the USB back into the computer. Check the status.
    iii. Restart the software and later the computer if subsequent attempts could not connect the detector to the computer.
    iv. Contact customer service if the detector to the computer connection issue persists.
2. Ensure all the connections to the fiber optic cable from the light source and detector are tight.
3. Ensure the light source is on and the high intensity dongle is fully plugged into the light source.
4. Re-clean the sampling window and probe.
5. If using the probe holder, ensure that the probe is flush with the flat surface of the probe holder.
    i. If not, a gap may have been created that allowed ambient light to enter the detector or the reflected light from the sample could not reflect as well.
6. Ensure all detector parameters are set appropriately.
7. Conduct another background scan with the white reflectance tile and then rescan the sample.
8. If the problem of poor quality spectra persists, select the “Wavelength & Wavenumber Correction” at the bottom of the software window.
    i. Attempt a “Self-Correction” first using the white reference tile and then click proceed. Scan the while reference tile as you would when doing a background scan.
    ii. When the instrument is operating properly, the user can also develop additional correction tests with different samples to ensure reliability. The user needs to ensure the following when selecting a reference material for a new wavelength/wavenumber correction test:
        1. Easily accessible and plentiful.
        2. Does not degrade (or very minimally).
        3. Available in high purity easily.
        4. Generates spectral features.
9. Potential issue with the fiber optic cable:
    i. If the light from the light source to the probe seems darker than usual or light is absent when the source is on at the end of the probe, there might be a break in the fiber optic cable between the light source and probe.
    ii. Unplug the fiber optic cable from the detector and then shine a light onto the sampling window of the probe. If no light appears at the end that connects to the detector, the fiber optic cable is broken from the probe to the detector.
    iii. Replace if necessarily, but contact an expert to ensure, especially if the cable had been bent too far.
10. Contact customer service if a problem persists.

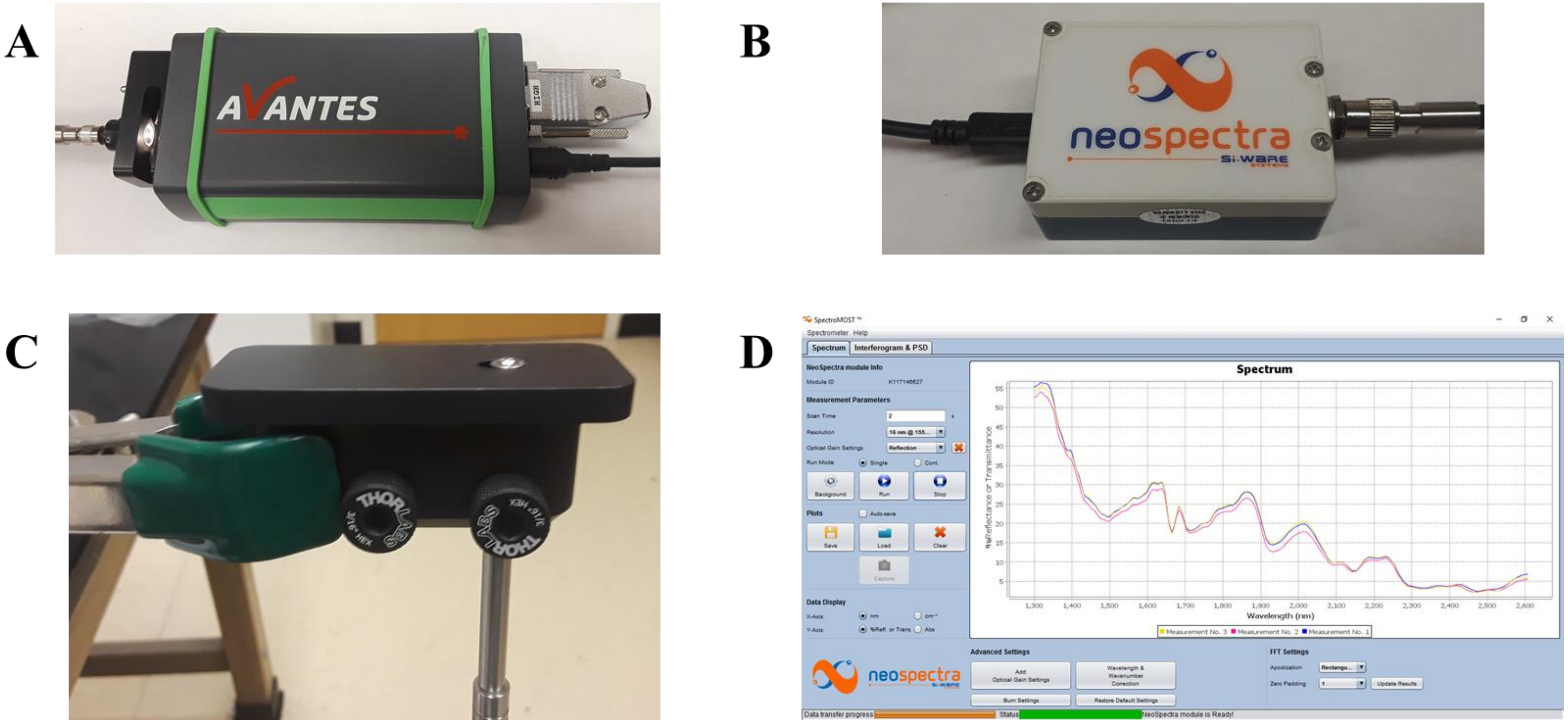

Fig S5.4. Images and screenshots of the Neospectra 2.5
(A) Image of the light source for the Neospectra 2.5 set-up. **(B)** Image of the Neospectra 2.5, the detector for the set-up. **(C)** Image of the arrangement for the sampling probe set-up. The black probe holder is held by a clamp. The end of the silver fiber optic probe is pointing up and parallel to the floor. Tablets are then placed on top and end of the probe to be sampled. **(D)** Screenshot of the data being collected with the Neospectra software SpectroMOST

.

Text S5 F. The NIR-S-G1 spectrometer operating protocol

NIR-S-G1 operating procedures:

The NIR-S-G1 (beta version "NIRscan" evaluated) used in this work consisted of two separate devices; a near-infrared sampling unit and a smartphone that runs an Android® based operating system. The near infrared sampling unit contains all the hardware necessary for sampling the target (light source, sampling window, optics, and detector) and operates cooperatively with the smartphone. The smartphone, a Motorola G4 Plus-Android version 7.0, acts as the unit's user graphical interface, command module for the sampling unit, and data storage for the device. Communication between the sampling unit and smartphone is achieved using Bluetooth® wireless technology. Both the sampling unit and smartphone are powered by internal lithium ion batteries and can be recharged using the same micro-USB cable. To operate the NIR-S-G1, once both devices are fully powered, the user must activate the "PillScanNIR" application on the smartphone **(Fig S5.5A)**. From the main menu of the app, the user selects the type of analysis desired (i.e. through blister pack or not) **(Fig S5.5B)**. The library spectrum with which the sampling unit will compare its experimental results is then selected by the user from a list of available spectra of medicines sorted by brand names **(Fig S5.5C)**. After library spectrum selection, the phone uploads the sampling and calibration protocol to the sampling unit**(Fig S5.5D)**. Tablets are rested on top of the sampling window (blistered samples are placed and held flush against the sampling window) **(Fig S5.5F)**. Using either the phone or a button on the sampling unit **(Fig S5.5G)**, a scan is initiated on the sample of interest. The spectral data is then uploaded from the sampling unit to the smartphone and computationally analyzed. After a few seconds**,** the smartphone displays whether the experimentally collected spectra successfully matched the selected library spectra **(Fig S5.5H)**..

NIR-S-G1 reference library operating details:

The reference library spectra were generated at the Global Good/Intellectual Ventures laboratory, the developer of the Android application PillscanNIR, because the device was not ready for reference library generation by a user at the time of the study. Reference genuine samples were shipped to the Global Good laboratory where the libraries were generated and uploaded to the smartphone application.

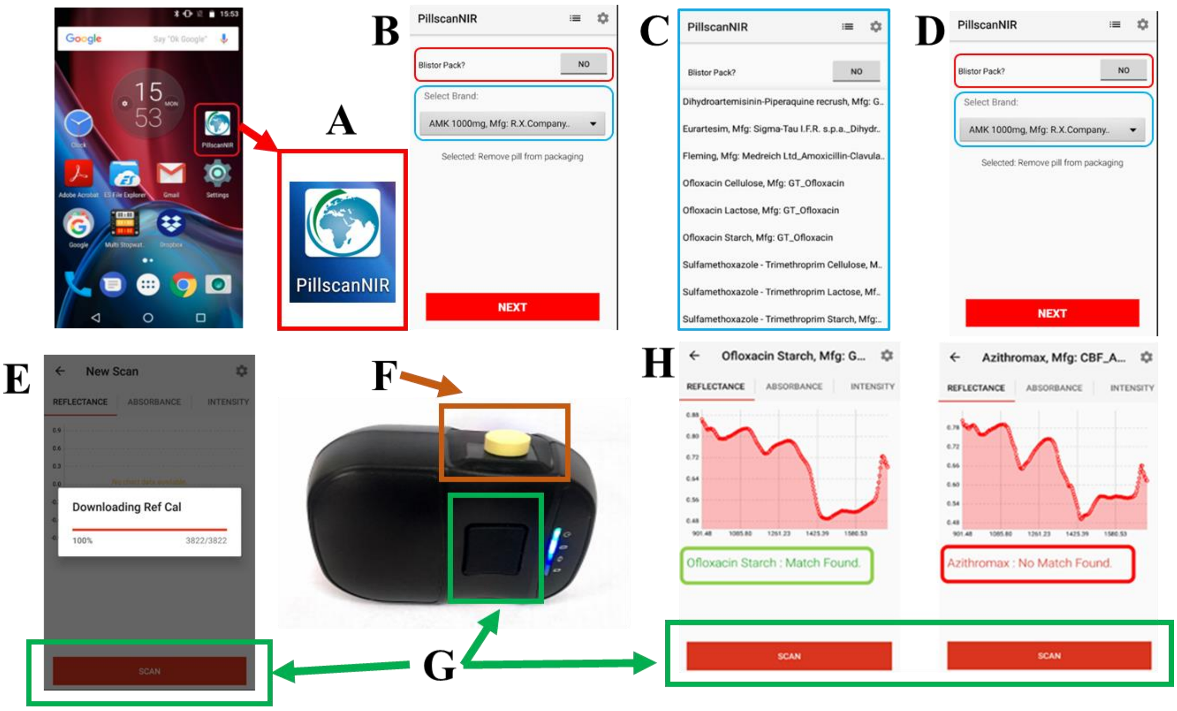

Fig S5.5. Screenshots and images of the NIR-S-G1

Images and screen captures adapted from PillScanNIR Operating Instructions for Android from Dr. Ben Wilson at Global Good/Intellectual Ventures. **(A)** Screen shot of the home screen with the magnified image of the PilscanNIR app used to operate the NIR-S-G1. **(B)** Screenshot of the main screen of the PilscanNIR app. **(C)** Screenshot of the list of medicines with reference spectra to select and compare to. **(D)** Screenshot after library reference selection. **(E)** Screenshot of the progress bar after hitting "Next". The software is uploading the parameters to the NIR-S-G1 and app. **(F)** Image of the tablet placed on top of the sampling interface of the NIR-S-G1. **(G)** Locations to start a scan on the NIR-S-G1. A rubber button on the NIR-S-G1 and the "Scan" button before or after a medicine is tested will start the scan. **(H)** Screenshot of a matching result on the left, mismatch on the right.

Text S5 G. PADs operating protocol

Protocols for the PADs were provided at the time of purchase by Dr. Marya Lieberman from the University of Notre Dame. Below is a summary of the protocol and instructions received.

For the PADs sample preparation and testing protocol, the user needs to:

1. Crush the questioned medicine into a fine powder.
2. Take several milligrams of the powdered medicine and apply it to the middle of the card. Arrows printed on the card designate the location where the powdered medicine should be applied. The application line runs perpendicular to the lanes of the card that contain reagents labeled “A” through “L”.
3. Using a popsicle stick, small spatula, or similar tool, press the powdered medicine to embed it into the PAD. Ensure a uniform amount of the medicine throughout the application line for each lane on the PAD.
4. Fill a container or dish with water until it reaches a height of about 5 mm.
5. Stick the bottom of the PAD in the water and prop the top of the card so it stands up in the water container. The top of the card contains a QR code and the PAD identification number. Let the PAD stand in the water for 3 minutes.
6. Remove the PAD from the water and allow the card to develop and react with the reagents on the card for an additional 3 minutes.
7. Write information about the sample on the card, take a photograph of the card, and compare the colors of the PAD to reference photographs of the API of interest.

Fig S5.6. PADs reference library photos.

Water (negative control)	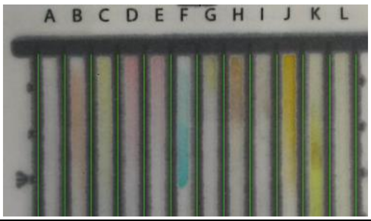	To be compared to API cards
Amoxicillin – clavulanic acid	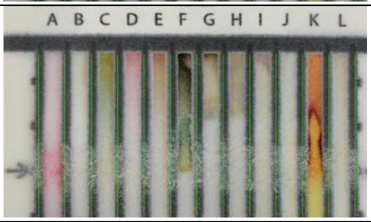	C: Green (may range from yellow-green to brown-green)<br>F: Dark green (lane often gets “stuck”<br>K: Cherry red
Azithromycin	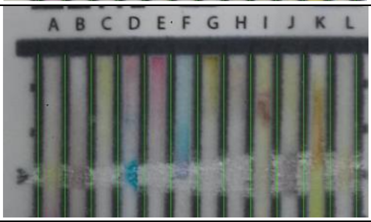	D: Turquoise/blue color at swipe line<br>F: Purple color at swipe line
Dihydroartemisinin - Piperaquine	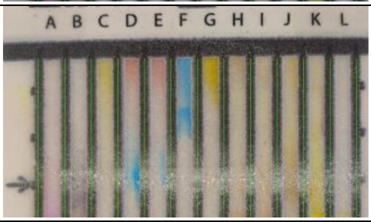	D: Blue at swipe (fades quick)<br>E: Blue at swipe (less intense than D)
Ofloxacin	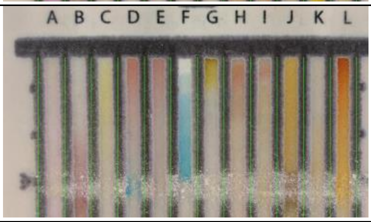	D: Blue (fades quickly)<br>J: Many dosage forms contain starch, giving black at swipe line<br>L: Orange
Sulfamethoxazole - Trimethoprim	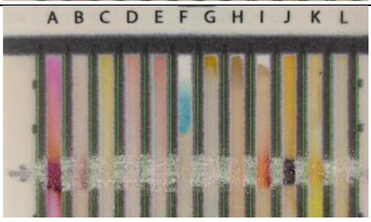	A: Red/purple<br>I: Orange/red at swipe line<br>J: Black at swipe line in dosage forms that contain starch

Photos from the reference images provided by Dr. Marya Lieberman from the University of Notre Dame.

Text S5 H. The PharmaChk microfluidic system operating protocol

The PharmaChk's user manual and step-by-step protocol were provided at the time of testing for by the Zaman Laboratory at Boston University. Step-by-step instructions were also displayed on the computer that controlled and acted as the interface for the PharmaChk microfluidic system. Below is a brief summary of the operational protocol at time of testing.

<u>To prepare PharmaChk solutions, the user needs to:</u>

1. Prepare the probe solution
    a. For artesunate, the solution contains hematin, fluorescein, and luminal in a sodium hydroxide solvent
2. Prepare the reference standard solutions
    a. For artesunate, dissolve the API in a sodium hydroxide solution and dilute to the following concentrations:
        i. 100 %
        ii. 50 %
        iii. 10 %
3. Take each solution created and load them into a 10 mL syringe and remove any air bubbles that accumulate in the syringe.

<u>To set up the PharmaChk instrument, the user must:</u>

1. Plug in the power and USB cables to the PharmaChk.
2. Plug the USB cables into the computer and connect the power to the mains.
3. Power on both the computer and the PharmaChk.
4. Follow the on-screen instructions.
5. Attach all the tubing to the microfluidic cartridge, load it into the PharmaChk, and close the lid.
6. Attach all the syringes from the solution preparations to their respective tubing and secure the syringes in the cradle.
7. Adjust the focus of the cameras if required.
8. Fill up the diluent cup with the API specific diluent, add the diluent tube to the cup, and ensure the waste cup is empty.

<u>To load samples onto PharmaChk, the user has to:</u>

1. Continue to follow the on-screen instructions.
2. Fill the dissolution cup with 50 mL of sodium hydroxide solution.
3. Insert the API tube through sonicator and book a filter at the end of the tube.
4. Add the medicine to the dissolution cup.
5. Submerge the probe and API tube filter in solution.
6. Press "Begin" on the computer.
7. Analyze the results at the end of the experiment.

<u>To clean PharmaChk, the user must:</u>

1. Continue to follow the on-screen instructions
2. Dispose of all chemical waste left in the dissolution cup and dispose of the filter at the end of the API tube.
3. Clean the sonicator.
4. If conducting more experiments, repeat loading the sample.
5. If no more experiments need to be conducted, dispose of all the solutions in the PharmaChk. This includes the syringes and the waste cup.
6. Refill all the syringes with clean water, reattach to the PharmaChk, and flush the system.
7. Refill all the syringes with air, reattach to the PharmaChk, and flush the system.
8. Repeat steps 6 and 7.
9. Dispose of the solution in the waste cup.

Text S5 I. The Progeny spectrometer operating protocol

Overview of the operating procedures:
After turning on the device and logging in, the instrument verification test is recommended prior to scanning any samples. Performance verification is a daily test done to ensure that the instrument is performing within acceptable specification. The user presses on the shield icon on the bottom left of the screen and selects the standard material and runs the sample analysis. The given results were pass or fail. After a successful test, the user goes to “Scan” function which contains three analysis modes: application, batch and analyze modes. The Progeny spectrometer basically operates in two modes: Analyze and Application. Batch mode is form of application mode for measuring multiple batches.

In the Analyze mode, the instrument compares the detected data to its “Master Library” of spectral profiles and then calculates a correlation coefficient (CC) value for each profile. The instrument identifies the substance named in the spectral profile with the top match. Match or No Match will be returned as a result. It is most useful for identifying samples of unknown substances.

For the Application mode, the instrument collects the spectral data using a specified collection of pre-set instrument settings and custom libraries, which means the sample is compared only to the *sub-library* of spectral profiles to calculate the correlation value. Pass or fail will be returned as a result. It is useful for either identifying or verifying samples from known collections of substances.

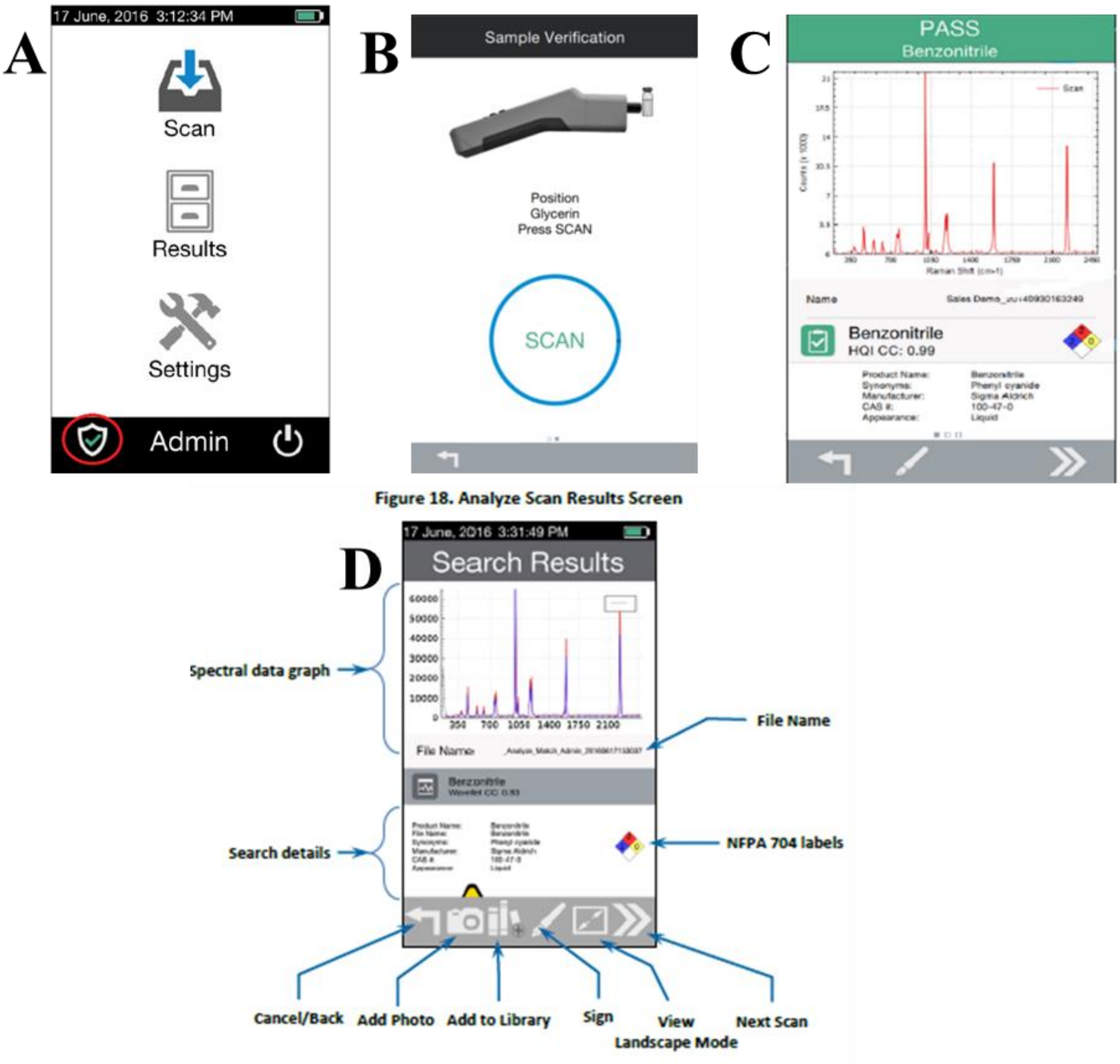

Fig S5.7. Screenshots of the Progeny

Screen captures adapted from the Rigaku Progeny™ and Progeny™ LT Analyzer User Manual (Version Date, 2016) **(A)** Screenshot of the main menu of the Progeny. **(B)** Screenshot before a scan is started. **(C)** Screenshot of the results screen after a preselection of reference library spectra and scan of benzonitrile. **(D)** Screenshot of the results after an “Analyze” experiment that searches the entire library of the instrument.

Progeny reference library features overview:

To create a reference library for the Progeny Raman spectrometer, the user only needs one good spectrum of the sample. The reference sample is scanned in “Analyze Mode”. After the spectrum is collected, the user can add the spectrum to the master library or a sub-library in a specific method for the instrument. After the library entry is created, the user can add a description or chemical information to that entry.

For Progeny sub-libraries:

All library spectra generated are saved to the Master library. For example, spectra from pharmaceutical samples could be mixed with spectra from food samples during normal analysis. Sub-libraries are used so the user can define what sub-set of libraries to focus the device on.

1. From the main menu, press “Settings” **(Fig S5.7A)**.
2. Press “Library”.
3. Press the “Add Library Symbol” button on the bottom right hand of the screen.
4. Enter the desired name of the Sub-Library and press the check mark button.
5. The user has created a new Sub-library.
6. To add previously acquired spectra, select the desired library.
7. Press the “Add Spectra Symbol” button (looks like an EKG) on the bottom right of the screen.
8. The user can write the name of the spectra, or immediately press the check mark to scroll through the entire master library.
9. Select one or multiple spectra by double pressing each selection and when done, press the check mark.
10. To generate new spectra, see protocol below.

For generating new library spectra on the Progeny, the user must:

1. Ensure the device is turned on and that proper sample holder/space is attached to the instrument.
    a. For a tablet, use the tablet holder that attaches to the spacer.
    b. For blister pack samples, just use the spacer.
2. Have the sample in the proper position in front of the sampling interface.
3. Press the “Scan” button in the main menu.
4. Press the “Analyze” button.
5. Press “Arm Laser” to prepare the instrument for analysis.
6. Pres “Scan” on the instrument to complete the experiment **(Fig S5.7B)**.
7. Look at spectra and determine if suitable for library.
    a. See if the spectrum has sharp peaks, the baseline is flat, and the signal is in the thousands (ideal, but lower ones acceptable if and only peaks are well defined, not just on big hump).
8. Press the “Add Library Button” if the spectrum looks acceptable.
9. Type in the name of the sample in the “Enter Material Name”. Once done, press the check mark.
    a. Include at least this information in the following order with a “_” in between each:
        i. YearMonthDate (Ex. 20170927).
        ii. Brand Name.
        iii. Active Ingredient(s).
        iv. Batch Number.
        v. ***Optional*** User Initials to determine who made the library.
10. Add the spectra to the desired library.
    b. “Master Library” contains every library spectrum that was collected by the instrument.
    c. Other results are user generated libraries that narrow down the samples you want to analyze known as a “sub-library”.

        i. Each "sub-library" has defined experimental conditions that the user can set.
11. The library spectrum is ready for use.

<u>Progeny application development protocol:</u>
Applications are used to for the user to pre-select a library spectrum to find out if the experimental sample matches the library spectra. The device will look at other spectra in the master library, but only if the samples fails. The user can define in applications what the pass threshold for pass or failures to ensure a certain percentage of confidence. To make an application, the user should:

1. Press the "Settings" button from the main menu.
2. Press the "Applications" button.
3. Press the "Add Application Symbol" button on the bottom right of the screen.
4. Type in the name of the application and press the check mark.
5. Select the application you just named.
6. Adjust the parameters to desired settings'
    a. For pharmaceutical analysis, most of the default parameters should work fine. The user can select a sub-library they created specific to the types of samples being analyzing. The user can also adjust the "Pass Threshold" to define at which point a sample passes or fails based on the closeness of the match.
7. Press the check mark when done.
8. Application should have saved.

<u>For the Progeny instrument calibration protocol, the user needs to:</u>

- Daily/Weekly Calibration (Performance Verification)
    1. Press the "Shield" symbol on the bottom left hand corner of the main menu.
    2. Select "Performance Verification".
    3. Select the sample you have from the defined list (the one that came with the instrument is the best).
    4. Attach the correct sampling interface (use the vial holder for the benzonitrile sample).
    5. Press "Arm Laser" to prep the sample.
    6. Press "Scan" when ready to conduct the experiment.
    7. The "Result" window should pop up.
        a. If pass, add comments if you would like by pressing the "Pen" symbol at the bottom of the screen. Go to the home menu and conduct experiments.
        b. If failure:
            i. Restart the device and re-test.
                1. Remove the battery after shutdown to ensure the device completely turns off.
            ii. If failure again:
                1. Conduct an instrument calibration.
- Monthly/Every Couple Months Calibration (Instrument Calibration).
    1. Press the "Shield" symbol on the bottom left hand corner of the main menu.
    2. Select "Instrument Calibration".
    3. Select "Benzonitrile".
    4. Attach the vial holder to the instrument and insert the benzonitrile vial.
    5. Press "Arm Laser".
    6. Press "Scan" when ready to conduct the experiment.
    7. The result window should pop up:
        a. If pass, add comments if you would like by pressing the "Pen" symbol at the bottom of the screen. Go to the home menu and conduct experiments.
        b. If failure:
            i. See Troubleshooting protocol.

<u>For the Progeny troubleshooting protocol, the user needs to:</u>

1. Ensure the sample is properly secured or being held in front of the sampling interface or vial holder.
    a. Minimize and potential ambient light from entering the device.
    b. Hold the sample in one position and do not move.
2. Ensure the battery is more than 1/3 charged (just in case it is a power issue).
    a. The user can charge and use the device at the same time using the instrument holder.
3. When in doubt, restart the device.
    a. Remove the battery after shutdown to ensure the device completely turns off.
4. Conduct a performance verification to ensure it is not an instrument problem.
    a. If the performance verification passes, most likely it is a sampling issue.
5. If the performance verification FAILS, perform an Instrument Calibration.
6. If the instrument calibration FAILS, restart the device and remove the battery.
    a. Allow the instrument to sit without the battery for 5 minutes to ensure no charge remains in the instrument.
7. Perform another instrument calibration.
8. If it fails again, contact the experts.

To sync Progeny results to computer, the user must:

1. Connect Progeny to the USB port on the PC with provided min-USB to USB cable.
2. Wait for PC to recognize the new USB connection.
3. Type http://Progeny into the website browser.
4. Select and download the desired file name or all files.
5. The file would be in PDF form in the zipped file.

Text S5 J. Rapid Diagnostic Tests operating protocol

A step-by-step protocol for each API was provided by the manufacturer at the time of testing the RDTs. Below is a brief summary of the protocol.

For RDT sample preparation and testing, the user needs to:

1. Extract the API in 95% alcohol to the defined concentration for the API by the manufacturer.
2. Dilute by placing a drop of extract into a defined volume of water in a suitable container to make dilution #1.
3. Perform a second dilution by taking a drop of dilution #1 and placing into a defined volume of water in a second container to make dilution #2.
4. Dispense 3 droplets of dilution #2 into the sample well of the RDT and wait 5 minutes.
    a. If the red control line does not appear, the test is invalid and a new RDT must be used to test again.
    b. If only the red control line appears, the medicine is determined to be good quality.
    c. If the red control line and the red test line appear, the sample may be substandard or falsified.
        i. Continue to step 5 to confirm the sample to be either substandard or falsified.
5. With a new RDT, dispense 3 droplets of dilution 1 into the sample well of the RDT and wait 5 minutes.
    a. If the red control line does not appear, the test is invalid and a new RDT must be used to test again.
    b. If only the red control line appears, the medicine is determined to be substandard.
    c. If the red control line and the red test line appear, the sample is falsified.

Table S5.2. Sample Preparations for RDTs

Active Ingredient	**Artemether***	**Artesunate**	**Dihydroartemisinin**
Target Extraction Concentration based on Labeled Content	2 mg/mL	2 mg/mL	2 mg/mL
Volume of Dilution #1	5 mL	5 mL	2.5 mL
Approximate Dilution #1 Concentration	10 µg/mL	10 µg/mL	20 µg/mL
Volume of Dilution #2	5 mL	5 mL	2.5 mL
Approximate Dilution #2 Concentration	50 ng/mL	50 ng/mL	200 ng/mL

* Results from these experiments were discarded due to unreliable performance.

Text S5 K. The Truscan RM spectrometer operating protocol

<u>Truscan RM overview of the operating procedures:</u>
After turning on the device, the user logs in with their credentials and goes through a self-test daily to ensure optimal performance. The user selects “Self-Test” and places the reference sample in the holder; after a few seconds the screen produces a pass or a fail. After a successful test, the user goes to the “Run” function, selects the library spectra defined as “Methods” that they would like to compare to, and the user inputs the filename of the sample. To sample tablets out of the packaging, the user places the tablet into the tablet holder and a nose cone for sampling through packaging. The user then starts the scan and “Pass” or “Fail” will display as a result.

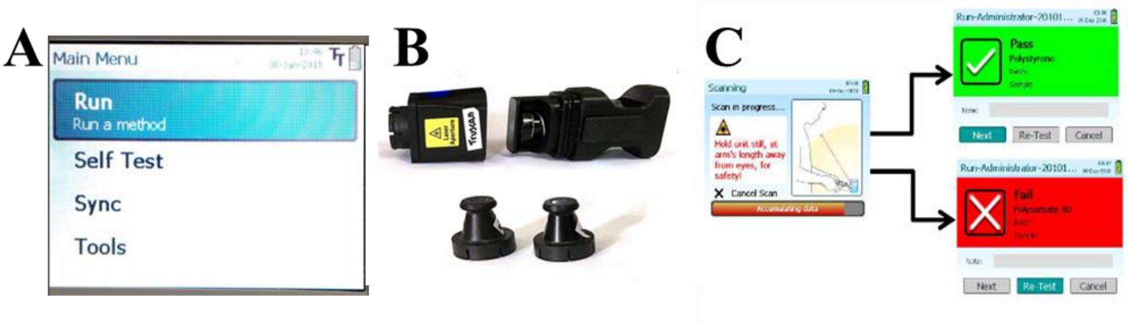

Fig S5.8. Screenshots and images of the accessories for the Truscan RM.
Photos adopted from the Truscan RM Analyzer User Guide (September 2016) **(A)** Screenshot of the main menu of the Truscan RM. **(B)** Image of the accessories or the Truscan RM. Top left is the connector for the tablet holder that attached to the Truscan RM. Top right is the tablet holder that is then inserted into the sleeve of the connector. Bottom two cones attach to the instrument Truscan RM and act as a spacer for the optimal focal distance when sampling a solid sample like a tablet. **(C)** Left, screenshot during a scan. Right, pass and fail results after a scan.

<u>Truscan RM reference library features overview:</u>
When generating reference library spectra on the TruScan RM, the user collects one good quality spectrum of the sample. The spectrum collected by the instrument is defined as a “signature” by the instrument. This signature is saved on the Truscan RM; however, it is in an inactive state and cannot be immediately used as a reference to test new samples with. The Truscan RM must be then connected and communicate to an external master computer. On the master computer, the signatures saved on the Truscan RM can be enabled or disabled for use as a reference library entry. Further details about connecting the Truscan RM to a computer and generating libraries are found below.

<u>TruScan RM methods (generating libraries):</u>

- For generating Signatures on the TruScan RM, the user should:
    1. From the main menu, select “Tools”.
    2. Select “Acquire Signature”.
    3. Position the sample in front of the sampling interface.
        a. Using the vial holder for small vials.
        b. Using the tablet holder for pills and tablets.
        c. Using the nose cone for bulky samples.
    4. Select “Start Scan”.
    5. After the analysis, confirm the spectra looks good.
        a. Sharp peaks, flat baseline, no big humps.
    6. Highlight and select the “Name” row.
    7. Include at least this information in the following order with a “-” in between each:
        a. YearMonthDate (Ex. 20170927).
        b. Brand Name.
        c. Active Ingredient(s).
        d. Batch Number.

    e. ***Optional*** User Initials to determine who made the library.
8. After the name of signature has been written, highlight and select “Save”.
9. Repeat the steps to collect other signatures for other samples.

- <u>For uploading the signatures to the computer, the user needs to.</u>
    1. Ensure the TruScan RM is on and that the user is signed in into the main menu.
    2. Open the bottom cover of the TruScan RM, but DO NOT open the inner compartment with the battery.
    3. Insert the mini-USD like cable from the Ethernet cable adapter to the TruScan RM.
        a. The connection is just above the battery compartment.
    4. Connect an Ethernet cable from the adapter to the computer.
    5. Access Internet Explorer on the computer.
    6. On the “Address” bar, type in the following IP address if it has not been saved: 192.168.99.100
    7. Sign in to the “Web Admin Login” with the same credentials as the device sign in.
    8. Select and press the “Signatures” tab on the browser.
    9. In the dropdown box (top left corner of the browser under “Methods” and “Signatures”), select “Inactive Signatures.
    10. Select the inactive signatures that you would like to use in the eventual library and then press “Activate Selected”.
    11. To generate a new library entry, select “Add Method”.
        a. If the user just wants to add signatures to an old method, just select the old method from the “Methods” menu and add them.
    12. Fill in the “Method Name” line.
    13. Include at least this information in the following order with a “-” in between each:
        a. YearMonthDate (Ex. 20170927).
        b. Brand Name.
        c. Active Ingredient(s)
        d. Batch Number .
        e. ***Optional*** User initials to determine who made the library.
    14. At the bottom of the webpage in the “Unattached Signatures” box, highlight the desired spectra for the method.
    15. Click the “←” button and this should add the signatures to the method.
        a. ***The user can add multiple spectra to one method***
            i. Ex. Different batches of the same sample.
    16. Once selected the appropriate signatures, click “Add Method” button on the bottom.
    17. To ensure the method has been appropriately uploaded, go back to the TruScan RM.
        a. From the main menu, select “Run”.
        b. Highlight and select method.
        c. Ensure the method you created in the computer browser is there.
    18. Unplug the adapters and cables and the user should be ready to go.

<u>Truscan RM calibration:</u>

- <u>For a daily/weekly calibration (self-test), the user needs to:</u>
    1. From the main menu, select “Self-Test”.
    2. Attach the vial holder to the sample interface and insert the polystyrene sample from the case into the holder.
        - Ensure the label on the sample is placed on the opposite side of the instrument sample interface/LCD screen.
    3. Highlight the sample ID, press enter, and type in the following information:
        - Include at least this information in the following order with a “-” in between each:
            - YearMonthDate (Ex. 20170927).
            - User initials to determine who made the calibration.
    4. Highlight “Start Test” and hit enter.

5. The Result window should pop up.
    - If pass, go to the home menu and conduct experiments.
    - If failure:
        - Repeat the test.
        - If fails again, restart the device and re-test.
            - Remove the battery after shutdown to ensure the device completely turns off.
        - If failure again:
            - Conduct an instrument certification.

- <u>For annual calibration (instrument certification), the user should:</u>
    - This certification calibrates the instrument up to factory grade specifications, only needs to be done once a year to achieve "Good Manufacturing Practice" status.

1. From the main menu, select "Tools".
2. Highlight and select "Instrument Certification".
3. Attach the vial holder to the sample interface and insert the polystyrene sample from the case into the holder.
    - Ensure the label on the sample is placed on the opposite side of the instrument sample interface/LCD screen.
4. Highlight the sample ID, press enter, and type in the following information:
    i. Include at least this information in the following order with a "-" in between each:
        1. YearMonthDate (Ex. 20170927).
        2. Sample Initials (PS).
        3. User initials to determine who made the calibration.
5. Highlight "Start Test" and press enter.
6. Replace the polystyrene sample with a vial of cyclohexane.
7. Highlight the sample ID, hit enter, and type in the following information:
    a. Include at least this information in the following order with a "-" in between each:
        i. YearMonthDate (Ex. 20170927).
        ii. Sample initials.
        iii. User initials to determine who made the calibration.
8. Highlight "Start Test" and press enter.
9. Replace the cyclohexane sample with a vial of acetaminophen.
10. Highlight the sample ID, press enter, and type in the following information:
    a. Include at least this information in the following order with a "-" in between each:
        i. YearMonthDate (Ex. 20170927).
        ii. Sample Initials (ACET).
        iii. User Initials to determine who made the calibration
11. Highlight "Start Test" and hit enter
12. The Result window should pop up.
    a. If pass, go to the home menu and conduct experiments.
    b. If failure:
        i. Consult troubleshooting protocol.

<u>For Truscan RM troubleshooting protocol, the user must:</u>

1. Ensure the sample is properly secured or being held in front of the sampling interface or vial holder.
    c. Minimize any potential ambient light from entering the device.
    d. Hold the sample in one position and do not move.
2. Ensure the battery is more than 1/3 charged (just in case it is a power issue).

    a. The user can charge and use the device at the same time using the instrument holder.
3. When in doubt, restart the device.
    a. Remove the battery after shutdown to ensure the device completely turns off.
4. Conduct a Self-Test to ensure it is not an instrument problem.
    a. If the self-test passes, most likely it is a sampling issue.
5. If the self-test FAILS, perform an Instrument Certification.
6. If the instrument certification FAILS, restart the device and remove the battery.
    a. Allow the instrument to sit without the battery for 5 minutes to ensure no charge remains in the instrument.
7. Perform another instrument certification.
8. If it fails again, contact customer service.

<u>Truscan RM downloading software to a computer:</u>

- For setting up the TruScan RM for the network (since the user is using an Ethernet cable), the user has to:
    1. Login to the analyzer.
    2. Select Tools > press the Enter key > System Settings > press the Enter key.
    3. Select Network > press the Enter key > select Use DHCP > use the arrow key to set.
    4. Use DHCP to No.
    5. Select IP Address > press the Enter key. Using the keypad, enter 192.168.99.100
    6. Select Done.
    7. In the Netmask field, enter 255.255.255.0
    8. Delete values in the Subnet, Gateway, and DNS fields.
    9. Press the Esc key and save the settings.
    10. Press the Esc key again to return to the Main menu.

- For setting the computer for the TruScan RM network (Windows 7 or XP ***Pro***), the user needs to:
    1. From Control Panel, select Network and Sharing Center.
    2. Select Change adapter settings.
    3. Select Local Area Network. Right click for Properties.
    4. Select Internet Protocol Version 4.
    5. If this network card will be used exclusively to connect to the TruScan RM analyzer, select the General tab > select Use the following IP address > set the IP address to 192.168.99.99. The subnet mask field will self-populate (255.255.255.0). Leave all other fields blank.
    6. Click OK.

- For opening the firewall to the TruScan RM, the user has to:
    1. On a designated sync server, open Windows Firewall settings by clicking Start > Control Panel.
    2. Double-click Windows Firewall.
    3. If Windows Firewall is currently turned OFF, you may skip the rest of this procedure. If Windows Firewall is turned ON, click the Exceptions tab, and then click Add Port.
    4. In the Name field, enter a descriptive name (we recommend TruScanSyncServer), and enter 8083 in the Port Number box. Click OK.
    5. Repeat step 3 and step 4 until ports 8083, 8084, 8085, 8086, 8087, 8088, 8089, 8090, 8091, and 8092 have each been added. You will add a total of 10 ports.
    6. Click OK to complete the settings.

- For setting up software to export data from the TruScan RM to the computer, the user needs to:
    1. Ensure the TruScan RM is on and that the user is signed on into the main menu

2. Open the bottom cover of the TruScan RM, but DO NOT open the inner compartment with the battery.
3. Insert the mini-USD to Ethernet cable adapter to the TruScan RM.
    - The connection is just above the battery compartment.
4. Connect an Ethernet cable from the adapter to the computer.
5. Access “Internet Explorer” on the computer.
6. On the “Address” bar, type in the following IP address if it has not been saved: (192.168.99.100)
7. Sign in to the “Web Admin Login” with the same credentials as the device sign in.
8. Click the Archives tab.
9. Click Download the SyncServer Installation Kit.
10. In the File Download - Security Warning dialog, click Run.
11. In the TruScan RM SyncServer Setup Wizard, click Next.
12. Select an Installation folder > click Next.
13. In the Config Settings dialog, set path folders > click Continue.
14. Let the installer complete, including the novaPDF 7 printer setup.
15. In the Installation Complete dialog, click Close.
16. Go to Control Panel > Devices and Printers.
17. Go to novaPDF Properties > Advanced tab.
18. Select Print directly to the printer. Click Apply, then click OK.

- To run a “Sync Test” to ensure proper connectivity between the TruScan RM and computer, the user must:
    1. Form the main menu of the TruScan RM, Select Tools > press the Enter key > select System Settings > press the Enter key.
    2. Select Network > press the Enter key > select Diagnostics > press the Enter key.
    3. Select Sync Test > press the Enter key.
    4. If the TruScan RM passes, the user should be all set.
        - If failure, look again through the protocol above.
            - Also reference the TruScan RM manual.
            - Keep in mind, home editions of Microsoft Windows will not work, Windows 7 an XP should work (Pro Only).

<u>To sync Truscan RM results, the user should:</u>

1. Connect the TruScan RM to the computer via Ethernet cable.
2. In main screen of device, select sync then go.
3. When it is completely synced, the items to send would be 0.
4. The folder located in the archive folder where the software was installed.
5. The folder named by the date of synchronization and the files would be in PDF form.

Text S5 L. The QDa mass spectrometer operating protocol

For sample preparation, the user needs to:

6. Weigh the pill.
7. Crush and homogenize the pill in aluminum foil or weighing paper.
8. Weigh out the sample (around 15 mg).
9. Add the extraction solution to the powdered sample to create the desired concentration (**Table S5.4**).
10. Shake well, let settle for 5 minutes.
11. Take an aliquot of the extraction and dilute to the Dilution #1 concentration using the dilution solution (**Table S5.4**).
12. Shake well, let settle for 5 minutes.
13. Take an aliquot of Dilution #1 and dilute to the Dilution #2 concentration using the dilution solution (**Table S5.4**).
14. Shake well, let settle for 5 minutes.
15. The Dilution #2 will be injected into the QDa.

To prepare QDa standards, the user must:

1. Weigh out approximately 15 mg of the standard.
2. Add the extraction solution to the powdered sample to create the desired concentration (**Table S5.4**).
    a. For samples with multiple APIs, the extraction solutions will be combined in the final dilution (**Table S5.4**).
3. Shake well, let settle for 5 minutes.
4. Take an aliquot of the extraction solution and dilute to the Dilution #1 concentration using the dilution solution (**Table S5.4**).
5. Shake well and let settle for 5 minutes.
6. Take and aliquot of Dilution #1 and dilute to the various calibration standard concentrations using the dilution solution (**Table S5.4**).
    a. The concentration of Dilution #2 for the samples is in the middle of the calibration curve. Use 5 total standards, 2 calibration standards with concentrations above and 2 below the middle standard.
7. Shake well and let settle for 5 minutes.

To prepare the QDa, the user has to:

1. Turn on the QDa and allow it to warm up for 5 – 10 minutes.
2. Turn on the computer, open MassLynx 4.0, go to the “MS Console”, and ensure the software is communicating correctly with the instrument **(Fig S5.9A)**
3. Turn on nitrogen gas flow (100 psi input) and wait for 10–15 minutes to the “Console” in minutes.
4. Recalibrate and perform the resolve check both tests utilize the internal calibrant of the QDa.
5. Upon successful recalibration and resolution, proceed to step 6. If calibration fails, follow the steps below.
    i. If the calibration fails, retry again.
    ii. If the calibration fails a second time, reset the QDa under “Control”, “Reset QDA”, and try again.
    iii. If it fails a third time, reset the instrument to factory defaults.
    iv. If it fails a fourth time, then power cycle the instrument and computer.
    v. If it fails a fifth time, contact customer service.
6. Turn on the solvent pump to allow the mobile phase solution to flow into the QDa and the appropriate flow rate (**Table S5.4**) and wait 30 minutes for the signal to settle.
    a. Ensure the capillary voltage is on and in the correct ionization mode (access through the Tune Page) to see the signal.

To set the method, the user has to:

1. Open the "MS Method".
2. Create a new MS method file.
    a. Note: establish a common naming convention to be used for every MS method that details the specifics of the settings in the MS method.
3. Set the parameters to the settings for the specific API (**Table S5.4**).
4. Save the MS method.
5. Ensure the correct MS method is used for the correct API.

To prepare QDa for data collection, the user has to:

1. In MassLynx, add an experimental run **(Fig S5.9B)**
    a. Note: establish a common naming convention to be used for every experimental run ensuring that the specifics of each run are included such as the date, API being tested, initials of technician, goal of experimental run, etc.
2. Select the correct MS method file (**Table S5.4**).
3. Start the experimental run.
4. Open the chromatogram (highlight experimental run in MassLynx, double click "Chromatogram") and ensure the baseline is stable.

To set QDa calibration standards, the user must:

1. After the baseline is stable, run a few injections of the mobile phase through the sample loop into the QDa detector to ensure the sample loop is clean (no signal increase from the mobile phase injection).
2. When injecting any solution into the six-port injector with a 20 μL sample loop (calibration standard, samples, or mobile phase), use the following steps **(Fig S5.9C)**:
    a. Use a syringe to transfer around 120 μL into the "Load" setting of the 6-port injector.
    b. Swiftly move the lever on the six-port injector from "Load" to the "Inject" setting.
    c. Follow the signal on the chromatogram until the signal reaches the baseline (around 2 minutes for the calibration standards or samples).
    d. When the solution is back to the baseline level for a brief period (again around 2 minutes) then inject again.
    e. Each solution should be injected at least 3 times following the steps above.
    f. Once all injections have been completed for the calibration standard or sample, inject around 120 μL of the mobile phase at least 2 times to clean out the six-port injector and sample loop.
    g. Proceed to the next solution (calibration standard or sample).
3. Begin with the calibration standard with the lowest concentration following the steps above.
4. After completing all calibration standard injections, wash the sample loop with the mobile phase solution injecting around 120 μL at least 3 times.

For sample injections, the user must:

1. Starting with the first sample, follow the solution injection steps in the "Calibration Standard" section above
2. Remember to perform at least 3 injections of each sample ensuring that the signal is reliably back to the baseline each time and washing the sample loop between each standard using the mobile phase solution (see steps above).
3. When all samples have been injected, wash the sample loop with the mobile phase solution injecting around 120 μL (using a 20 μL sample loop) at least 3 times.
4. In MassLynx, click the "Stop" button to end the experimental run.

For data analysis, the user has to:

1. Open the chromatogram file for the experimental run to be analyzed.
2. Click “Display”, “Mass”, under “Channels” double click the desired mass(es) (**Table S5.3** “m/z for Analysis”).
3. Click “Process”, “Smooth”
   a. For “Window size (scans) +/-“ use “5”
   b. For “Number of smooths” use “3”
   c. For “Smoothing method” select “mean”
   d. Click “OK”
4. Click “Edit”, “Integrate Peaks” **(Fig S5.9D)**
   a. Highlight the base of the peak from the beginning to the end (where the signal goes up to when it hits the baseline level), click “Add”, and record the abundance.
5. Record all abundances along with their corresponding calibration standard concentration or sample name in Microsoft Excel.
6. To create the calibration curve, highlight the data from the calibration standards, click “Insert”, “Scatter Plot”.
7. Click on the calibration curve and name it according to the chosen naming convention.
8. Click “Chart Design”, “Quick Layout”, “Layout 9” which has the “f(x)” symbol on it to give the equation of the calibration curve in the y = mx +b format.
9. Use the equation of the calibration curve to determine the concentration (x) of each sample injection (y) in Excel.
10. The “Target Concentration” is the concentration that a good quality medicine should have after the extraction and two dilutions. This is the same concentration as middle of the calibration standards (**Table S5.4**).

Notes for DHAP:

1. When making the extraction for the standard solution, to obtain the correct ratio of piperaquine to dihydroartemisinin, approximately 150 mg of piperaquine should be weighed out creating an extraction solution of approximately 50 mL. Extra time and sonication may be needed to fully dissolve the powder in the extraction solvent (5 minutes of sonicating, wait 5 minutes). The extraction solution for the samples should be sonicated and vortexed as well.
2. Ensure that the correct initial molecular weight is used when calculating the concentrations of DHAP. For example, our standard powder used for the calibration standards was piperaquine tetraphosphate tetrahydrate ($C_{29}H_{32}Cl_2N_6 + 4(H_3PO_4) + 4(H_2O)$) with a molecular weight of 999.55 g/mol, while the medicines being tested were made from piperaquine tetraphosphate ($C_{29}H_{32}Cl_2N_6 + 4(H_3PO_4)$) with a molecular weight of 927.48 g/mol. The appropriate molecular weights should be used throughout all the calculations, but for piperaquine it can be particularly confusing.
3. There are two protocols for DHAP (**Table S5.4**), one for the quantitation of dihydroartemisinin and the other for the quantitation of piperaquine. A single protocol was not found to work for both APIs.

Table S5.3. Table of instrument parameters for the QDa.

	QDa Instrument Parameters*				
**Active Pharmaceutical Ingredient(s)**	**MS Mode**	**Probe Temperature (°C)**	**Capillary Voltage (kV)**	**Cone Voltage (V)**	**m/z of the ion for analysis**
Amoxicillin /Clavulanic Acid	Negative	500	0.8	5	A: 364.00 $[M-H]^-$<br>CA: 198.10 $[M-H]^-$
Artesunate	Positive	500	1.5	10	407.30 $[M+Na]^+$
Artemether/ Lumefantrine	Positive	500	1.5	5	AM: 321.16 $[M+H]^+$<br>LM: 528.16 $[M+H]^+$
Azithromycin	Positive	500	1.5	10	375.26 $[M+2H]^{2+}$
Dihydroartemisinin/ Piperaquine	Positive	500	1.5	10	DHA: 307.15 $[M+Na]^+$<br>P: 268.76 $[M+2H]^{2+}$
Ofloxacin	Positive	400	1.4	10	362.10 $[M+H]^+$
Sulfamethoxazole/ Trimethoprim	Positive	400	1.4	10	SM: 254.06 $[M+H]^+$<br>TM: 291.15 $[M+H]^+$

* The following parameters for the QDa were consistent throughout all the experiments: source temperature was 120 °C, solvent flow rate was 0.3 mL/min, detector gain was 10, sampling frequency was 2 Hz, and injection volume for the sample loop was 20 µL.

Table S5.4. Sample preparation methods for the QDa.

	QDa Sample Preparation Methods					
**Active Pharmaceutical Ingredient(s)**	**Extraction Solvent**	**Mobile Phase/Dilution Solvent**	**API Standard Extraction Concentration (mg/mL)**	**API Standard Calibration Concentration Range (ppm)**	**Sample Extraction Concentration (mg/mL)**	**Sample Final Dilution Concentration (ppm)**
Amoxicillin /Clavulanic Acid	Water	2:8 Water:Methanol with 2mM Ammonium Acetate	A: 0.6<br>CA: 0.6	A: 0.875 to 7<br>CA: 0.125 to 1	A: 3<br>CA: 0.375	A: 4<br>LM: 0.56
Artesunate	Methanol	8:2 Water:Methanol with 0.1% Acetic Acid	2.5	0.1 to 1	2.5	0.5
Artemether/ Lumefantrine	9:1 Methanol:Acetic Acid	2:8 Water:Methanol with 0.1% Acetic Acid	AM: 3<br>LM: 3	AM: 0.1 to 1<br>LM: 0.6 to 7.0	AM: 0.5<br>LM: 3.0	AM: 0.7<br>LM: 4.2
Azithromycin	Methanol	8:2 Water:Methanol with 0.1% Acetic Acid	5	0.1 to 1	5	0.5
Dihydroartemisinin in DHAP	Methanol	8:2 Water:Methanol with 0.1% Acetic Acid	1	0.01 to 0.1	1	0.05
Ofloxacin	98:2 Water:Acetic Acid	8:2 Water:Methanol with 0.1% Acetic Acid	5	0.1 to 1	5	0.5
Piperaquine in DHAP	99:1 Methanol:Hyrdrochloric Acid	8:2 Water:Methanol with 0.1% Acetic Acid	2	1 to 3	2	2.5
Sulfamethoxazole/ Trimethoprim	Methanol	8:2 Water:Methanol with 0.1% Acetic Acid	SM: 5<br>TM: 1	SM: 0.5 to 2.5<br>TM: 0.1 to 0.5	SM: 1.5<br>TM: 0.3	SM: 1.5<br>TM: 0.3

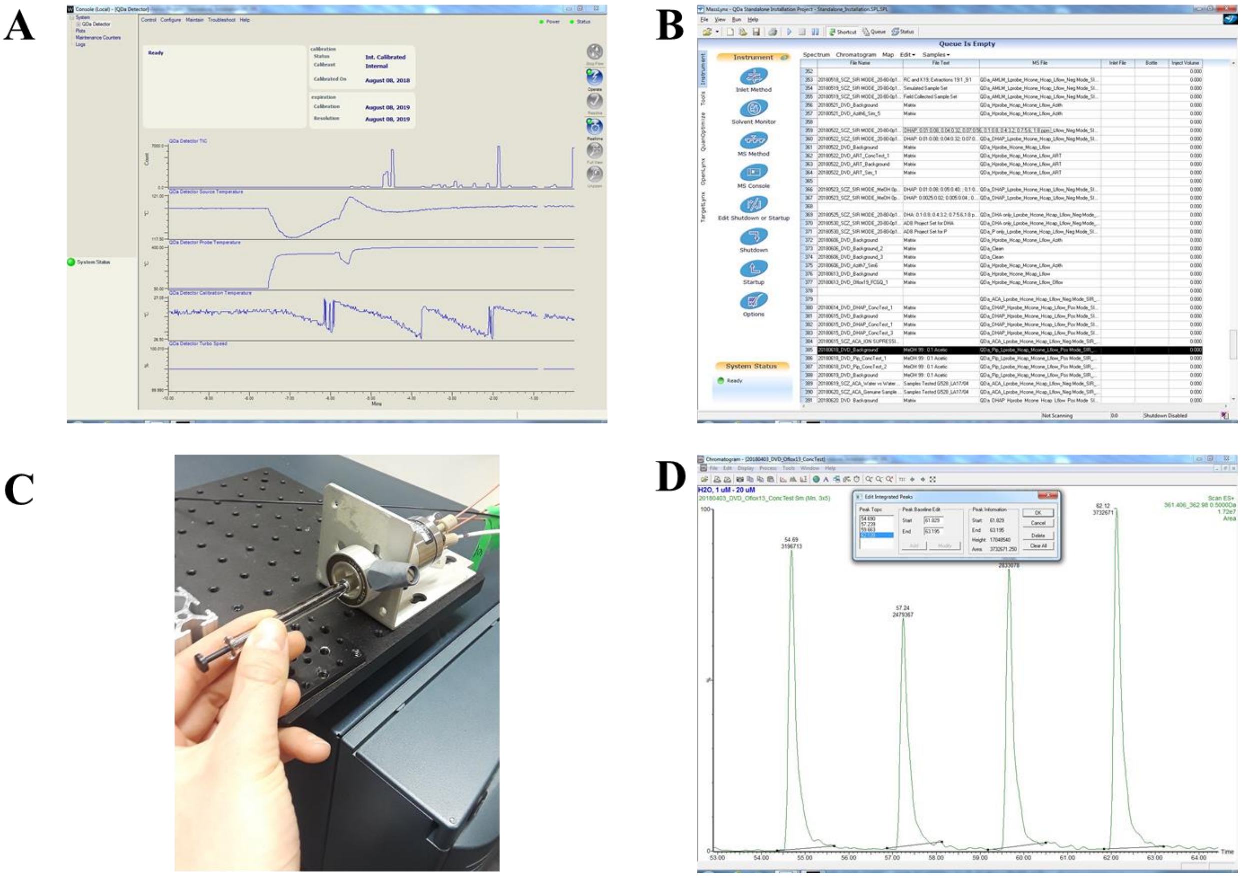

Fig S5.9. Screenshots and images illustrating QDa operation
(A) Screenshot of the QDa control window. **(B)** Screenshot of the sample list window. **(C)** Image of the six-port injector being loaded by syringe. **(D)** Screenshot of processing the total ion chromatogram for the API of interest
