# Supplementary material for: Laboratory evaluation of twelve portable devices for medicine quality screening
Source: PLoS Negl Trop Dis. 2021 Sep 30;15(9):e0009360. doi: 10.1371/journal.pntd.0009360 (PMC8483346; doi:10.1371/journal.pntd.0009360)
Supplement: S6 Appendix — (PDF) [file pntd.0009360.s006.pdf]

S6 Appendix. Field collected medicines tested and UPLC results.

Study Code (each blister of the sample)	Study Phase (RL, LE)	Brand name	API name	API Strength (mg)	Expiry Date (mm/yyyy)	Formulation	Types of packaging (T, O)	Origin - Quality of Sample	Mass Spectrometry Result	UPLC Result (%)
G071	RL	Sulfatrim	SMTM	400-80	04/2018	Tab	O	FC - Genuine	N/A	88†-89†
G072	RL	Sulfatrim	SMTM	400-80	10/2017	Tab	O	FC - Genuine	N/A	93-95
G080	RL	Vactrim	SMTM	400-80	04/2016	Tab	O	FC - Genuine	N/A	93-98
G137	RL	Ofloxin	OFLO	200	03/2016	Tab	O	FC - Genuine	N/A	89.9†
G259	RL	Ofloxacin	OFLO	200	01/2017	Tab	O	FC - Genuine	N/A	94.2
G275	RL	Oflocee	OFLO	200	04/2018	Tab	O	FC - Genuine	N/A	89.1† (1st test)<br>96 (2nd test)
G278	RL	Azithromax	AZITH	250	07/2017	Tab	O	FC - Genuine	N/A	102
G281	RL	Di-flo	OFLO	200	02/2017	Tab	O	FC - Genuine	N/A	92.4
G311	RL	Strim-Side	SMTM	200	06/2017	Tab	O	FC - Genuine	N/A	96-93 (1st test)<br>99-99 (2nd test)
G314	RL	Vactrim	SMTM	250	09/2016	Tab	O	FC - Genuine	N/A	97-98
G317	RL	Oflocee	OFLO	200	12/2020	Tab	O	FC - Genuine	N/A	89.2† (1st test)<br>92.3 (2nd test)
G318	RL	Augmentin	ACA	200	03/2018	Tab	T	FC - Genuine	N/A	92-96
G324	RL	Azithromax	AZITH	250	08/2018	Tab	O	FC - Genuine	N/A	95 (1st test)<br>98 (2nd test)
G337	RL	Azithromax	AZITH	250	06/2018	Tab	O	FC - Genuine	N/A	97
G344	RL	Di-flo	OFLO	200	03/2018	Tab	O	FC - Genuine	N/A	93.4

Study Code (each blister of the sample)	Study Phase (RL, LE)	Brand name	API name	API Strength (mg)	Expiry Date (mm/yyyy)	Formulation	Types of packaging (T, O)	Origin - Quality of Sample	Mass Spectrometry Result	UPLC Result (%)
G388	RL	Artemether-Lumefantrine	AL	200	09/2016	Tab	T	FC - Genuine	N/A	115[†]-102
G419	RL	Strim-Side	SMTM	200	03/2019	Tab	O	FC - Genuine	N/A	96-98
G426	RL	Ofloxin	OFLO	200	01/2019	Tab	O	FC - Genuine	N/A	94.4
G429	RL	Biseptrim	SMTM	60	05/2018	Tab	O	FC - Genuine	N/A	96-100
G432	RL	Vactrim	SMTM	60	07/2018	Tab	O	FC - Genuine	N/A	94-135[†] (1st test)<br>95-135[†] (2nd test)<br>94-114[†] (3rd test)<br>93-132[†] (4th test)
G435	RL	Ofloxacin	OFLO	200	08/2018	Tab	O	FC - Genuine	N/A	93.9 (1st test)<br>93.4 (2nd test)<br>97.4 (3rd test)
G437	RL	Sulfatrim	SMTM	200	09/2020	Tab	O	FC - Genuine	N/A	80[†]-81[†] (1st test)<br>84[†]-86[†] (2nd test)<br>87[†]-89[†] (3rd test)
G429	RL	D-Artepp	DHAP	250	09/2017	Tab	O	FC - Genuine	N/A	90.2-99.1
G485	RL	Azithromax	AZITH	250	03/2019	Tab	O	FC - Genuine	N/A	99
G526	RL	D-Artepp	DHAP	60	03/2017	Tab	O	FC - Genuine	N/A	86.9[†]-101.3
G528	LE	Cavumox 1G	ACA	200	03/2018	Tab	O	FC - Genuine	N/A	101-104
G529	RL	Cavumox 1G	ACA	250	02/2018	Tab	O	FC - Genuine	N/A	103-102
G530	RL	Cavumox 1G	ACA	200	09/2017	Tab	O	FC - Genuine	N/A	100-100
G533	RL	AMK 1000 mg	ACA	500	07/2018	Tab	T	FC - Genuine	N/A	100-77[†] (1st test)<br>97-54[†] (2nd test)
G534	RL	AMK 1000 mg	ACA	250	04/2018	Tab	T	FC - Genuine	N/A	99-78[†] (1st test)<br>99-52[†] (2nd test)
EP112/ EP113/ G563	LE	Biseptrim	SMTM	200	08/2019	Tab	O	FC - Genuine	N/A	96-101

Study Code (each blister of the sample)	Study Phase (RL, LE)	Brand name	API name	API Strength (mg)	Expiry Date (mm/yyyy)	Formulation	Types of packaging (T, O)	Origin - Quality of Sample	Mass Spectrometry Result	UPLC Result (%)
G546	LE	Di-flo	OFLO	200	03/2018	Tab	O	FC - Genuine	N/A	96.2
G547	RL	Artesun	ART	60	06/2019	Vial	T	FC - Genuine	N/A	96.7
G548	RL	Artesun	ART	60	05/2019	Vial	T	FC - Genuine	N/A	97.2
G549	LE	Artesun	ART	60	05/2019	Vial	T	FC - Genuine	N/A	98.7
G550	RL	D-Artepp	DHAP	500	02/2018	Tab	O	FC - Genuine	N/A	91.4-98.4
G551	RL	D-Artepp	DHAP	40-320	12/2017	Tab	O	FC - Genuine	N/A	87.3[†]-103
EP024/ G552	LE	D-Artepp	DHAP	40-320	01/2018	Tab	O	FC - Genuine	N/A	91.9-99.1
EP091 to EP100/ G556	LE	Vactrim	SMTM	400-80	08/2019	Tab	O	FC - Genuine	N/A	96-101
EP009/ EP010/ G563	LE	Augmentin	ACA	500-125	02/2016	Tab	T	FC - Genuine	N/A	101-97
G566	LE	Di-flo	OFLO	200	07/2018	Tab	O	FC - Genuine	N/A	96.9
EP044/ SPS14/ G569	LE	Oflocee	OFLO	200	03/2021	Tab	O	FC - Genuine	N/A	91.2 (1st test)<br>95.5 (2nd test)<br>96.2 (3rd test)
EP055/ EP056/ EP060/ G570	LE	Ofloxacin	OFLO	200	03/2020	Tab	O	FC - Genuine	N/A	92.4
EP129/ EP130/ G571	LE	Sulfatrim	SMTM	400-80	01/2022	Tab	O	FC - Genuine	N/A	98-99
GT-K19-AD	LE	Coartem	AL	20-120	01/2017	Tab	T	FC - Genuine	N/A	103-94
GT-K20-AD-3	RL	Coartem	AL	20-120	05/2017	Tab	T	FC - Genuine	N/A	103-94

Study Code (each blister of the sample)	Study Phase (RL, LE)	Brand name	API name	API Strength (mg)	Expiry Date (mm/yyyy)	Formulation	Types of packaging (T, O)	Origin - Quality of Sample	Mass Spectrometry Result	UPLC Result (%)
GT-K23-AD-3	RL	Coartem	AL	20-120	06/2017	Tab	T	FC - Genuine	N/A	106-93
LA 17-04	LE	AMK 1000 mg	ACA	875-125	06/2018	Tab	T	FC - Genuine	N/A	98-80† (1st test) 96-64† (2nd test)
LA13-02	LE	Griseofulvin	Griseofulvin	500	08/2015	Tab	T	FC - wrong API	N/A	No SMTM detected
LA16-113	RL	Azithromax	AZITH	250	06/2018	Tab	O	FC - Genuine	N/A	97
LA16-122	LE	Ofloxin	OFLO	200	12/2018	Tab	O	FC - Genuine	N/A	102.8 (1st test) 102.0 (2nd test)
EP144/ LA16-150	LE	Azithromax	AZITH	250	02/2018	Tab	O	FC - Genuine	N/A	102
LA16-17	RL	Strim-Side	SMTM	400-80	06/2016	Tab	O	FC - Genuine	N/A	99-98
LA16-180	RL	Ofloxin	OFLO	200	07/2018	Tab	O	FC - Genuine	N/A	92.8
LA16-202	RL	Augmentin	ACA	500-125	04/2018	Tab	T	FC - Genuine	N/A	99-102 (1st test) 99-102 (2nd test)
LA16-38	RL	Strim-Side	SMTM	400-80	05/2018	Tab	O	FC - Genuine	N/A	100-102
LA16-41	RL	Ofloxin	OFLO	200	10/2017	Tab	O	FC - Genuine	N/A	92.1
LA16-66	RL	Azithromax	AZITH	250	10/2018	Tab	O	FC - Genuine	N/A	100
LA16-70	LE	Strim-Side	SMTM	400-80	01/2018	Tab	O	FC - Genuine	N/A	91-92
LA17-03	RL	Augmentin	ACA	500-125	02/2019	Tab	T	FC - Genuine	N/A	99-103
*LC15	LE	Coartem	AL	20-120	01/2016	Tab	T	FC – 0% API	Major Components: Maltitol, Sucrose/Lactose, Glucose/Fructose, & Mannitol Minor Components: Levamisole	N/A
*LC18	LE	Coartem	AL	20-120	N/A	Tab	T	FC – 0% API	Major Components: Sucrose/Lactose & Glucose/Fructose Minor Components: Levamisole	N/A
*LC5	LE	Coartem	AL	20-120	11/2015	Tab	T	FC –	Major Components: Chloramphenicol Minor	N/A

Study Code (each blister of the sample)	Study Phase (RL, LE)	Brand name	API name	API Strength (mg)	Expiry Date (mm/yyyy)	Formulation	Types of packaging (T, O)	Origin - Quality of Sample	Mass Spectrometry Result	UPLC Result (%)
								0% API	Components: Levamisole & Sildenafil	
*LC9	LE	Coartem	AL	20-120	11/2015	Tab	T	FC – 0% API	Major Components: Ciprofloxacin Minor Components: Levamisole & Sildenafil	N/A
MM16-21	LE/RL	Artemether-Lumefantrine	AL	20-120	07/2017	Tab	T	FC - Genuine	N/A	101-93
*N1	LE	Coartem	AL	20-120	01/2016	Tab	T	FC – 0% API	Major Components: Mannitol, Sucrose/Lactulose, & Glucose/Fructose Minor Components: Maltitol	N/A
*N15	LE	Coartem	AL	20-120	01/2016	Tab	T	FC – 0% API	Major Components: Mannitol, Sucrose/Lactulose, & Glucose/Fructose Minor Components: Maltitol	N/A
*N19	LE	Coartem	AL	20-120	01/2016	Tab	T	FC – 0% API	Major Components: Sucrose/Lactulose, Glucose/Fructose, & Mannitol	N/A
*N3	LE	Coartem	AL	20-120	01/2016	Tab	T	FC - wrong API	Major Components: Sucrose/Lactulose & Glucose/Fructose Minor Components: Levamisole	N/A
*N34	LE	Coartem	AL	20-120	11/2015	Tab	T	FC - wrong API	Major Components: Chloramphenicol Minor Components: Sildenafil	N/A
*N36	LE	Coartem	AL	20-120	11/2015	Tab	T	FC - wrong API	Major Components: Ciprofloxacin Minor Components: Sildenafil	N/A
*N5	LE	Coartem	AL	20-120	11/2015	Tab	T	FC - wrong API	Major Components: Ciprofloxacin Minor Components: Sildenafil	N/A
*S0043	LE	Artemether-Lumefantrine	AL	20-120	06/2016	Tab	T	FC – 0% API	Major Components: Sucrose/Lactulose, Glucose/Fructose, Mannitol, and m/z 338 Minor Components: Maltitol	N/A

* Sample not tested by UPLC but underwent mass spectrometry as part of another study - none of the correct APIs as stated on the packaging were present
ACA: Amoxicillin-clavulanic acid; AL: Artemether-lumefantrine; API: Active Pharmaceutical Ingredient; ART: Artesunate; AZITH: Azithromycin; DHAP: Dihydroartemisinin-piperaquine; FC: Field-collected; LE: Laboratory evaluation; OFLO: Ofloxacin; O: Opaque packaging; RL: Reference library; SM: Simulated medicines; SMTM: Sulfamethoxazole-trimethoprim; Tab: Tablets; T: transparent packaging ; Vial: Vials (powder bottle for injection).
† Out of specification according to the 90-110% range considered in the present study.
N/A: Not applicable or lack of information.
