# Supplementary material for: Laboratory evaluation of twelve portable devices for medicine quality screening
Source: PLoS Negl Trop Dis. 2021 Sep 30;15(9):e0009360. doi: 10.1371/journal.pntd.0009360 (PMC8483346; doi:10.1371/journal.pntd.0009360)
Supplement: S7 Appendix — (PDF) [file pntd.0009360.s007.pdf]

S7 Appendix. Simulated Medicine Preparations

Text S7 A. Details about simulated medicine preparation 1

Table S7 A. Formulations of simulated medicine preparations.. 2

Text S7 A. Details about simulated medicine preparation

Two types of simulated medicines were developed: one set utilized pure API stocks purchased from TCI Chemical, and the other set was derived from genuine medicines that were crushed and re-pressed, as described in the table below (Table S7.1). The crushed and re-pressed samples were needed because of the large volume of simulated medicines necessary for significant statistics and to test a wide variety of APIs. The high cost of pure APIs also made re-crushing genuine samples the only feasible choice.

For the set of medicines that were derived from pure APIs, the ratios of API to excipient were derived from the following genuine medicines: Ofloxin 200 (OFLO), Vactrim (SMTM), Artesun (ART), and Azithromax (AZITH). Every simulated sample was prepared with one of each of the following (Table S7.1): cellulose, lactose, and starch. For example, there were three types of samples of simulated good quality OFLO where each sample contained one of the mentioned excipients. The simulated good quality samples with the re-crushed genuine medicines and ART did not contain any excipient and there was only one sample per API. To decrease the API concentration for the substandards of the re-crushed genuine medicines and ART, there were three samples at each API concentration, each sample diluted with one of the mentioned excipients. All pressed samples, except for powdered ART and the good quality re-crushed samples, also contained 2% by mass of magnesium stearate to help lubricate the sample when being pressed for easier removal.

All simulated medicines followed the same protocol for preparation, except for ART which is distributed in powder form. All the simulated sample ingredients that included the API, excipient, magnesium stearate, and crushed medicine powder, where applicable, were weighed on a scale to make approximately 15 tablets and placed into small, individual polyethylene sample bags and sealed. The ingredients were thoroughly mixed by massaging the sealed bag by hand until a homogeneous mixture appeared in the bag and screened through a mesh strainer to remove any clumps. Next, samples were weighed out in 100-mg aliquots. The aliquots were immediately pressed into 6-mm-diameter tablets, approximately 3- to 4-mm tall. For ART, samples were weighed out into 60-mg aliquots and placed into 6-mL, clear glass scintillation vials and sealed with a screw top. Tablet samples were stored in 6-mL amber glass scintillation vials and sealed with a screw cap until analysis. All samples were stored in a 4°C refrigerator.

Table S7 A. Formulations of simulated medicine preparations

Sample Category	Active Ingredient	API Source	Pure Active Ingredient (%)	Crushed Genuine Medicine (%)	Excipient (%)	Lubricant* (%)	Number of Unique Samples†
Good Quality	Ofloxacin	TCI Chemical	65		33	2	3
	Sulfamethoxazole/ Trimethoprim	TCI Chemical	80/16		2	2	3
	Azithromycin	TCI Chemical	73		25	2	3
	Artesunate	TCI Chemical	100		0	0	1
	Amoxicillin/ Clavulanic Acid	AMK 1000mg		100	0	0	1
	Dihydroartemisinin/ Piperaquine	D-Artepp		100	0	0	1
	Artemether/ Lumefantrine	Coartem		100	0	0	1
Substandard at 80%API	Ofloxacin	TCI Chemical	52		46	2	3
	Sulfamethoxazole/ Trimethoprim	TCI Chemical	64/13		21	2	3
	Azithromycin	TCI Chemical	58		40	2	3
	Artesunate	TCI Chemical	80		20	0	3
	Amoxicillin/ Clavulanic Acid	AMK® 1000mg		80	18	2	3
	Dihydroartemisinin/ Piperaquine	D-Artepp		80	18	2	3
	Artemether/ Lumefantrine	Coartem		80	18	2	3
Substandard at 50% API	Ofloxacin	TCI Chemical	33		65	2	3
	Sulfamethoxazole/ Trimethoprim	TCI Chemical	40/8		50	2	3
	Azithromycin	TCI Chemical	36		62	2	3
	Artesunate	TCI Chemical	50		50	0	3
	Amoxicillin/ Clavulanic Acid	AMK 1000mg	50		48	2	3
	Dihydroartemisinin/ Piperaquine	D-Artepp	50		48	2	3
	Artemether/ Lumefantrine	Coartem	50		48	2	3
Falsified	Acetaminophen	TCI Chemical	50		48	2	3
	Excipient Only	TCI Chemical	0		98	2	3

* Magnesium stearate

† 1 = only one sample that contains the active ingredient and has no additional excipient; 3 = three samples, the active ingredient plus one of the three excipients: cellulose, lactose, & starch
