# Supplementary material for: Laboratory evaluation of twelve portable devices for medicine quality screening
Source: PLoS Negl Trop Dis. 2021 Sep 30;15(9):e0009360. doi: 10.1371/journal.pntd.0009360 (PMC8483346; doi:10.1371/journal.pntd.0009360)
Supplement: S8 Appendix — (PDF) [file pntd.0009360.s008.pdf]

S8 Appendix. Brief sample handling protocols.

Text S8 A. The 4500a spectrometer 2
Text S8 B. The C-Vue liquid chromatograph 2
Text S8 C. The MicroPHAZIR RX spectrometer 2
Text S8 D. The Mini-lab TLC kit 3
Text S8 E. The Neospectra 2.5 spectrometer 3
Text S8 F. The NIR-S-G1 spectrometer 3
Text S8 G. PADs 4
Text S8 H. The PharmaChk microfluidic system 4
Text S8 I. The Progeny spectrometer 4
Table S8 A. Progeny sampling results interpretation 5
Text S8 J. Rapid Diagnostic Tests (lateral flow immunoassay) 5
Text S8 K. The Truscan RM spectrometer 5
Text S8 L. The QDa mass spectrometer 6

Text S8 A. The 4500a spectrometer

1. Each medicine was crushed into a homogenous mixture if not in powder form.
2. For each stock powdered sample, three independent spectra were recorded.
3. 10-15-mg samples were taken for each trial from the same stock of powder.
4. The sample window and press were cleaned in between each trial with delicate-task wipes and isopropanol.
5. The result was considered a "pass" if the expected medicine appeared in the six matches displayed at the end of the experiment with a coefficient higher than 0.9.

Text S8 B. The C-Vue liquid chromatograph

1. Each medicine was crushed, if not already in powder form, extracted, and diluted at least once.
2. 10-25-mg samples were taken for each medicine for extraction.
3. Calibration samples were prepared from a pure API stock.
4. Calibration standards for experiments performed on different days were prepared fresh for each day or stored in a 4°C refrigerator, and tested on the following day. No sample stored longer than a day in the refrigerator.
5. Three trials for every calibration sample were recorded and used to construct calibration curves.
6. Each questioned sample solution was tested three times, consecutively.
7. The signal for each questioned sample was interpolated into the calibration curve to determine the percentage of each API in that sample.
8. Calibration standards and unknown samples that contained two APIs were analyzed and quantitated from the same chromatogram.
9. Medicines containing less than 90% and more than 110% of the manufacturer's stated amount of API(s) were considered as a "fail".
10. For medicines with two APIs, both API had to be within specifications for the experiment to be considered a "pass".

Text S8 C. The MicroPHAZIR RX spectrometer

1. Prior to scanning, the genuine medicine reference spectral library must be selected.
2. Tablets and tablets in blister packaging had their own reference library to compare to.
3. Tablets from the same batch were scanned three times in the following way:
    a. Tablet #1, first face
    b. Tablet #1, opposite face
    c. Tablet #2, any face
4. Tablets in transparent blister packaging were from the same batch, and were scanned three times in the following way, with each scan being saved independently:
    a. Tablet #1
    b. Tablet #2
    c. Tablet #3
5. Tablets that were smaller than the sampling window of the device required the use of a sample cover to block ambient light. Blistered tablets did not require the use of such cover.
6. Artesunate samples were scanned through replacement glass vials except for the field-collected samples that were scanned through the manufacturer's glass vials.
7. The device output pass/fail results were recorded in the evaluation sheet.

Text S8 D. The Mini-lab TLC kit

1. Each medicine was crushed, if not in powder form, extracted, and diluted as per the protocol in the manual.
2. 10 mg–25-mg samples were taken for each medicine for extraction.
3. The reference standards were prepared from UPLC-confirmed genuine medicines using the whole tablet as per Mini-lab protocol.
4. Samples prepared were either tested on the same day or stored overnight in a 4 °C refrigerator to be tested the next day. No samples stored longer than a day in the refrigerator were tested.
5. The final sample dilution was tested three times on the same TLC plate.
6. If the results were inconsistent on a plate, where at least 1 of the 3 sample tested spots were inconsistent from two others., the entire TLC experiment was repeated on a different day.
7. TLC plates were interpreted and photographed immediately after TLC development and drying, where applicable.

Text S8 E. The Neospectra 2.5 spectrometer

1. Tablets and tablets in blister packaging had their own reference library to compare to.
2. Tablets from the same batch were scanned three times in the following way, with each scan being saved independently:
    a. Tablet #1, first face
    b. Tablet #1, opposite face
    c. Tablet #2, any face
3. Tablets in transparent blister packaging were from the same batch and were scanned three times in the following way, with each scan being saved independently:
    a. Tablet #1
    b. Tablet #2
    c. Tablet #3
4. Artesunate samples were scanned through replacement glass vials except the field-collected samples that were scanned through the manufacturer glass vials.
5. Three genuine medicine reference spectra were overlaid with the sample's spectra because of the lack of library functionality. The data were analyzed by a blinded investigator who did not conduct the physical experiments. The investigator noted which (if any) of the spectra were dissimilar. Dissimilar spectra were designated as poor-quality medicines.

Text S8 F. The NIR-S-G1 spectrometer

1. Prior to scanning, the genuine medicine reference spectral library must be selected.
2. Tablets and tablets in blister packaging had their own reference library to compare to.
3. Tablets from the same batch were scanned three times in the following way, with each scan being saved independently:
    a. Tablet #1, first face
    b. Tablet #1, opposite face
    c. Tablet #2, any face
4. Tablets in transparent blister packaging were from the same batch and were scanned three times in the following way, with each scan being saved independently:
    a. Tablet #1
    b. Tablet #2
    c. Tablet #3

5. Artesunate samples were scanned through replacement glass vials except the field-collected samples that were scanned through the manufacturer glass vials.
6. The device outputted pass/fail results that were recorded in the evaluation sheet.

Text S8 G. PADs

1. Each medicine was crushed, if not in powder form, immediately prior to conducting experiments.
2. About 20 to 40 mg of sample powder were applied to each PAD.
3. PADs were examined and photographed at least 3 minutes after development.
4. The water used for PAD development was replaced with fresh water between each experiment to prevent cross-contamination.
5. The same medicine would be tested once. If the experiment resulted in a "fail", the experiment would be repeated with a new PAD to confirm the result.

Text S8 H. The PharmaChk microfluidic system

1. Since the PharmaChk is only able to analyze ART, all the samples were in powder form and just needed to be extracted.
2. Whole medicine units were used for analysis as per protocol.
3. Medicines extraction occurred the same day as testing.
4. Calibration solutions were prepared as per PharmaChk protocol.
5. The extraction solution of each sample was tested three times.
6. Quantitative results were immediately displayed on the device's control computer.
7. Medicines containing less than 90% and more than 110% of the manufacturer's stated amount of API(s) were considered as a "fail".

Text S8 I. The Progeny spectrometer

1. The Analyze function was utilized for the Progeny spectrometer, followed by the Application function. Each trial was composed of three scans (One with the Analysis function followed by two with Application function).
2. Tablets and tablets in blister packaging had their own reference library for comparison.
3. Tablets from the same batch were tested three times in the following way, with each scan being saved independently:
   a. Tablet #1, first face
   b. Tablet #1, opposite face
   c. Tablet #2, any face
4. Tablets in transparent blister packaging were from the same batch and were scanned three times in the following way, with each scan being saved independently:
   a. Tablet #1
   b. Tablet #2
   c. Tablet #3
5. Field-collected tablets were held using the tablet holder.
6. Tablets in blisters were held by the operator's hand, flush against the nose cone of the instrument.
7. Artesunate powder samples were scanned through polyethylene bags.
8. The device outputted pass/fail results that were recorded in the evaluation sheet.

Table S8 A. Progeny sampling results interpretation

Scan 1	***Scan 2***	***Scan 3***	
Analyze	***Application 1***	***Application 2***	***OVERALL***
**Match**	Pass	Not necessary	PASS
**Match**	Fail	Pass	PASS
**Match**	Fail	Fail	Trial to be retested once and considered as a "fail" if inconsistency occurs again.
**No match**	Pass	Pass	PASS
**No match**	Pass	Fail	FAIL
**No match**	Fail	Pass	Trial to be retested once and considered as a "fail" if inconsistency occurs again.

Text S8 J. Rapid Diagnostic Tests (lateral flow immunoassay)

1. Each medicine was crushed, if not in powder form, extracted, and diluted once.
2. 10–25-mg samples were taken for each medicine for extraction.
3. Samples prepared were either tested that day or stored overnight in a 4°C refrigerator to be tested the next day. No samples stored longer than a day in the refrigerator were tested.
4. For falsified simulated samples (containing acetaminophen or excipients only), only the 2nd-to-last dilution was tested. In the RDT protocol, the 2nd-to-last dilution is tested to confirm the presence of the API, but it may substandard. For the falsified samples, if the more concentrated sample did not trigger an API detection, it was assumed the lower concentration solution would not trigger detection as well. If the API was detected in the 2nd-to-last dilution, the lowest concentration dilution was then tested. This was done to save as many RDTs as possible since they were single-use and only a limited supply was available.
5. When the control line did not appear, RDT experiments were discarded from analysis as per manufacturer's protocol.
6. RDTs were examined and photographed after at least 5 minutes of development.
7. The RDT protocol states it takes up to two RDT test per experiment to determine if a sample is substandard or falsified.
    a. If the red test line did not appear for the final sample dilution (lowest concentration), the sample is registered as "qualified" meaning the sample was deemed to be good quality, only one RDT was used and no further experiments were required as per protocol.
    b. If the red test line did appear for the final sample dilution (lowest concentration), the sample is registered as "falsified/substandard" meaning the sample was deemed to be poor quality. A second RDT experiment would be necessary using the second to last dilution to distinguish the sample from registering as "falsified" vs. "substandard".
        i. For the second experiment with the more concentrated sample, the presence of the red line registered the sample as being falsified. The absence of the red line registered the sample as being substandard.

Text S8 K. The Truscan RM spectrometer

1. Prior to scanning, the genuine medicine reference spectral library must be selected.
2. Tablets and tablets in blister packaging had their own reference library for comparison.

3. Tablets from the same batch were scanned three times in the following way, with each scan being saved independently:
    a. Tablet #1, first face
    b. Tablet #1, opposite face
    c. Tablet #2, any face
4. Tablets in transparent blister packaging were from the same batch and were scanned three times in the following way, with each scan being saved independently:
    a. Tablet #1
    b. Tablet #2
    c. Tablet #3
5. Tablets were analyzed with the tablet holder if they could fit and were strong enough.
6. Tablets that could not fit in the tablet holder and tablets that were analyzed through blister packs utilized the nose cone attachment.
7. Artesunate samples were scanned through clear polyethylene bags.
8. The device outputted pass/fail results and they were recorded in the evaluation sheet.

Text S8 L. The QDa mass spectrometer

1. Each medicine was crushed, if not already in powder form, extracted, and diluted at least once.
2. 10–25-mg samples were taken for each medicine for extractions.
3. Calibration samples were prepared from a pure stock of API.
4. Samples prepared were either tested the same day or stored overnight in a 4°C refrigerator to be tested the next day. No samples stored longer than a day in the refrigerator were tested.
5. Three trials for every calibration standard was recorded and used to construct the calibration curves.
6. Each questioned sample solution was tested three times back to back.
7. Each questioned sample was interpolated into the calibration curve to determine the percentage of each API in the prepared sample.
8. Calibration standards and questioned samples with two APIs were analyzed and quantitated from the same chronogram.
9. Medicines containing less than 90% or more than 110% of the manufacturer’s stated amount of API(s) were considered as “fail”.
10. For medicines with two APIs, both API had to be within specifications to be considered as a “pass”.
