# Supplementary material for: Laboratory evaluation of twelve portable devices for medicine quality screening
Source: PLoS Negl Trop Dis. 2021 Sep 30;15(9):e0009360. doi: 10.1371/journal.pntd.0009360 (PMC8483346; doi:10.1371/journal.pntd.0009360)
Supplement: S10 Appendix — (PDF) [file pntd.0009360.s010.pdf]

S10 Appendix. US, international, Chinese, and British pharmacopeial standards for the studied APIs.

API	***US Pharmacopeia 2017***	***International Pharmacopeia 2018***	***Chinese Pharmacopeia 2010***	***British Pharmacopeia 2018***
**Artesunate (IV/IM powder)**	N/A	90-110%	93-110%	N/A
**Amoxicillin/Clavulanic acid (tablet)**	90-110%	90-120%†	90-120%	90-105%
**Azithromycin (tablet)**	90-110%	N/A	90-110%	95-105%
**Sulfamethoxazole/Trimethoprim (tablet)**	93-107%	90-110%	N/A	92.5-107.5%
**Ofloxacin (tablet)**	90-110%	N/A	90-110%	N/A
**Dihydroartemisinin/Piperaquine (tablet)**	95-105%*	N/A	N/A	N/A
**Artemether/Lumefantrine (tablet)**	N/A	90-110%	N/A	N/A

* USP monograph, 2013 - Dihydroartemisinin/Piperaquine tablets monograph was not available in USP 2017
† Draft in preparation
