# Supplementary material for: Laboratory evaluation of twelve portable devices for medicine quality screening
Source: PLoS Negl Trop Dis. 2021 Sep 30;15(9):e0009360. doi: 10.1371/journal.pntd.0009360 (PMC8483346; doi:10.1371/journal.pntd.0009360)
Supplement: S11 Appendix — (PDF) [file pntd.0009360.s011.pdf]

S11 Appendix. API vs. formulation screening.

Specific API-detection	***Chemical formulation screening***
PharmaChk	Neospectra 2.5
C-Vue	NIR-S-G1
PADs	4500a FTIR
RDTs	MicroPHAZIR RX
Minilab	Progeny
QDa	Truscan RM
