# Supplementary material for: Laboratory evaluation of twelve portable devices for medicine quality screening
Source: PLoS Negl Trop Dis. 2021 Sep 30;15(9):e0009360. doi: 10.1371/journal.pntd.0009360 (PMC8483346; doi:10.1371/journal.pntd.0009360)
Supplement: S12 Appendix — (PDF) [file pntd.0009360.s012.pdf]

S12 Appendix. Mini-lab thin layer chromatography kit results

Table S12 A. Mini-lab thin layer chromatography kit detailed performance breakdown 1

Table S12 B. Mini-lab thin layer chromatography kit evaluation summary. 2

Table S12 A. Mini-lab thin layer chromatography kit detailed performance breakdown

	**Good-quality samples available for specificity calculation: *n*=24**			
	**0% API and wrong API samples (n=53)**		**50% and 80% API samples (n=42)**	**All poor quality samples (n=95)**
**Samples**	**Sensitivity (95% CI)**	**Specificity (95% CI)**	**Sensitivity (95% CI)**	**Sensitivity (95% CI)**
Total, not through packaging (n=119)	100 (93.3-100)	100 (85.8-100)	59.5 (43.3-74.4)	82.1 (72.9-89.2)
Antimalarials (n=51)	100 (87.7-100)	100 (47.8-100)	66.7 (41-86.7)	87 (73.7-95.1)
AL (n=24)	100 (79.4-100)	100 (15.8-100)	66.7 (22.3-95.7)	90.9 (70.8-98.9)
ART (n=14)	100 (54.1-100)	100 (15.8-100)	83.3 (35.9-99.6)	91.7 (61.5-99.8)
DHAP (n=13)	100 (54.1-100)	100 (2.5-100)	50 (11.8-88.2)	75 (42.8-94.5)
Antibiotics (n=68)	100 (86.3-100)	100 (82.4-100)	54.2 (32.8-74.4)	77.6 (63.4-88.2)
ACA (n=15)	100 (54.1-100)	100 (29.2-100)	83.3 (35.9-99.6)	91.7 (61.5-99.8)
AZITH (n=16)	100 (54.1-100)	100 (39.8-100)	33.3 (4.3-77.7)	66.7 (34.9-90.1)
OFLO (n=19)	100 (54.1-100)	100 (59-100)	50 (11.8-88.2)	75 (42.8-94.5)
SMTM (n=18)	100 (59-100)	100 (47.8-100)	50 (11.8-88.2)	76.9 (46.2-95)

Table S12 B. Mini-lab thin layer chromatography kit evaluation summary.

<table>
<tr><th rowspan="4">Sensitivity and Specificity Results</th><th><u>Samples</u></th><th><u>Sensitivity (95% CI)*</u></th><th><u>Specificity (95% CI)*</u></th><th><u>Comments</u></th></tr>
<tr><td>0% and wrong API</td><td>100 (93.3-100)</td><td rowspan="3">100 (85.8-100)</td><td rowspan="3">N/A</td></tr>
<tr><td>50% and 80% API[†]</td><td>59.5 (43.3-74.4)</td></tr>
<tr><td>All poor quality samples</td><td>82.1 (72.9-89.2)</td></tr>
<tr><th>Strengths and Limitations</th><td colspan="4">Strengths:
-High accuracy to identify samples with no or wrong API.
-Good sensitivity in identifying 50% API samples.

Limitations:
-Most 80% API samples incorrectly identified as genuine.
-Only three 80% API samples correctly identified as failing.[†]</td></tr>
<tr><th>User Satisfaction</th><td colspan="4">Plus:
All equipment necessary provided; well described, detailed and illustrated protocols; mains electricity not required.

Minus:
Safety hazards and waste needed because of chemical reagents; destroys sample; large and heavy; sample testing requires a relatively long time.</td></tr>
<tr><th>Comparative Evaluation</th><td colspan="4">-No significant differences in sensitivity compared to other devices to identify 0% and wrong API samples. Higher specificity than the C-Vue liquid chromatograph.
-Longest total time per sample compared to other devices.
-Several samples of the same API can be run simultaneously.</td></tr>
</table>

* Sensitivity and specificity for quality assessment of the dosage unit not through the packaging.
† Because TLC experiments of the samples tested are run together with 80% and 100% API reference standard solutions, the Mini-lab TLC methods allow a range of 80 to 100 % API as the lower and higher acceptable limits. These results should be thus interpreted with caution.
