# Supplementary material for: Laboratory evaluation of twelve portable devices for medicine quality screening
Source: PLoS Negl Trop Dis. 2021 Sep 30;15(9):e0009360. doi: 10.1371/journal.pntd.0009360 (PMC8483346; doi:10.1371/journal.pntd.0009360)
Supplement: S13 Appendix — (PDF) [file pntd.0009360.s013.pdf]

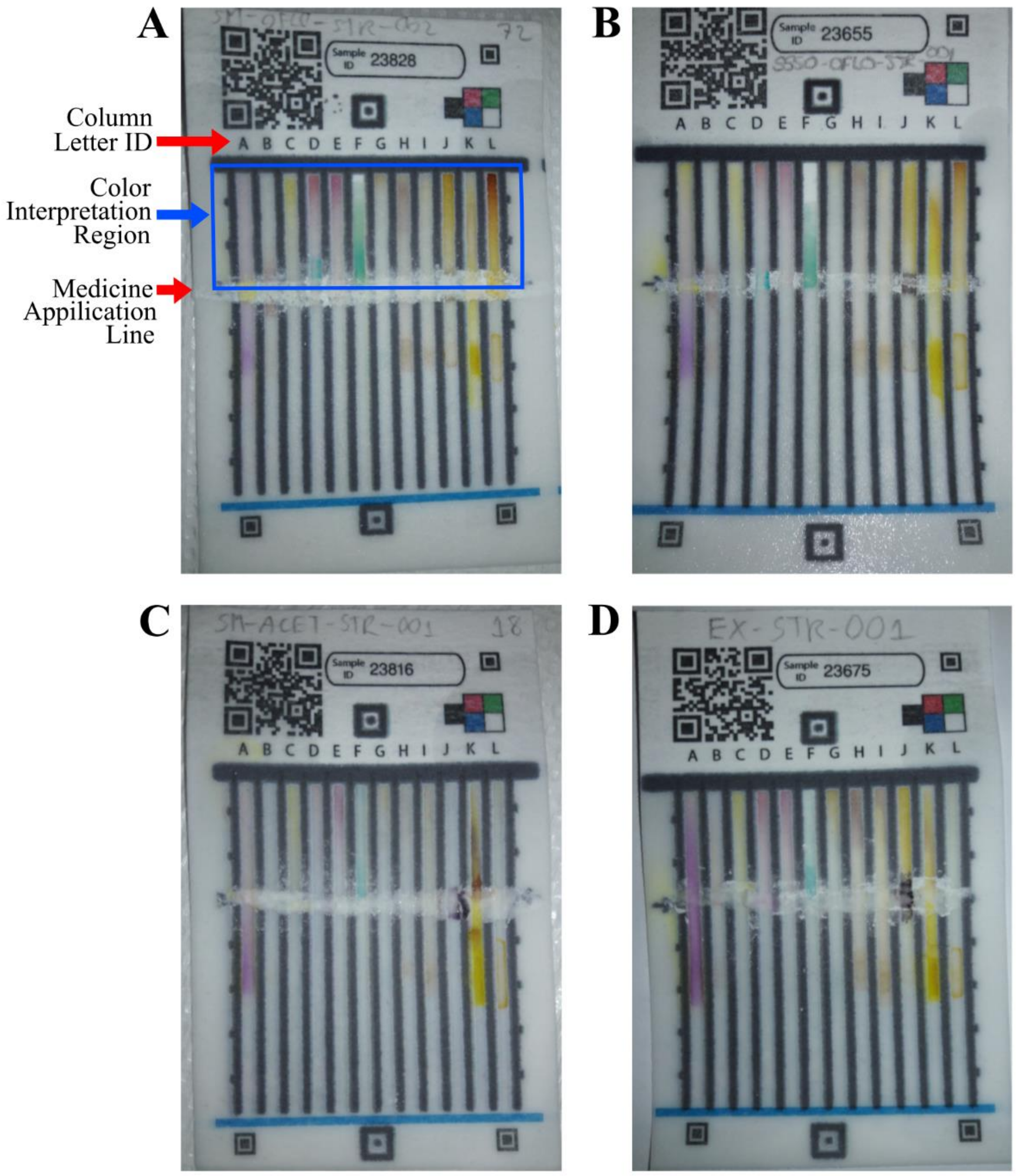

S13 Appendix. PADs color analysis.
Processed paper analytical devices (PAD) used for ofloxacin samples analysis. **(A)** A simulated 100% API ofloxacin tablet with starch as the excipient was classified as a “pass” by the investigator because of the presence of a blue color at the line of medicine application in column “D” and an orange color at the top of column “L”. Black color at line “J” confirmed the presence of starch in the sample. **(B)** A simulated sample containing 50% ofloxacin with starch as the excipient was classified as a “pass” because of the presence of a blue color at the line of medicine application in column “D” and an orange color at the top of column “L”. The presence of a black color at line “J” confirmed the presence of starch in the sample. **(C)** A simulated medicine containing acetaminophen and starch only, but no ofloxacin, was classified as a “fail” against the ofloxacin reference photo because of the lack of a blue color at the line of medicine application in column “D” and no orange color at the top of column “L”. The presence of a black color at line “J” confirmed the presence of starch in the sample. **(D)** A simulated medicine containing only starch but none of the expected API was classified as a “fail” against the ofloxacin reference photo due to the lack of a blue color at the line of medicine application in column “D” and an orange color at the top of column “L”. The presence of a black color at line “J” confirms the presence of starch in the sample.
