# Supplementary material for: Laboratory evaluation of twelve portable devices for medicine quality screening
Source: PLoS Negl Trop Dis. 2021 Sep 30;15(9):e0009360. doi: 10.1371/journal.pntd.0009360 (PMC8483346; doi:10.1371/journal.pntd.0009360)
Supplement: S14 Appendix — (PDF) [file pntd.0009360.s014.pdf]

S14 Appendix. Paper Analytical Devices (PADs) results

Table S14 A. Paper Analytical Devices (PADs) performance breakdown....................................... 1
Table S14 B. Paper Analytical Devices (PADs) single use device evaluation summary. 2

Table S14 A. Paper Analytical Devices (PADs) performance breakdown.

	**Good-quality samples available for specificity calculation: *n*=20**			
	**0% API and wrong API samples (n=31)**		**50% and 80% API samples (n=30)**	**All poor quality samples (n=61)**
**Samples**	**Sensitivity (95% CI)**	**Specificity (95% CI)**	**Sensitivity (95% CI)**	**Sensitivity (95% CI)**
Total not through packaging (n=81)	100 (88.8-100)	100 (83.2-100)	0 (0-11.6)	50.8 (37.7-63.9)
Antimalarials (n=13)	100 (54.1-100)	100 (2.5-100)	0 (0-45.9)	50 (21.1-78.9)
AL (n=0)*	N/A	N/A	N/A	N/A
ART (n=0)*	N/A	N/A	N/A	N/A
Piperaquine (n=13)*	100 (54.1-100)	100 (2.5-100)	0 (0-45.9)	50 (21.1-78.9)
Antibiotics (n=68)	100 (86.3-100)	100 (82.4-100)	0 (0-14.2)	51 (36.3-65.6)
Amoxicillin (n=15)*	100 (54.1-100)	100 (29.2-100)	0 (0-45.9)	50 (21.1-78.9)
AZITH (n=16)	100 (54.1-100)	100 (39.8-100)	0 (0-45.9)	50 (21.1-78.9)
OFLO (n=19)	100 (54.1-100)	100 (59-100)	0 (0-45.9)	50 (21.1-78.9)
Sulfamethoxazole (n=18)*	100 (59-100)	100 (47.8-100)	0 (0-45.9)	53.8 (25.1-80.8)

* AL, ART, Dihydroartemisinin, trimethoprim, clavulanic acid cannot be tested with the device.

Table S14 B. Paper Analytical Devices (PADs) single use device evaluation summary.

<table>
<tr><td rowspan="4">Sensitivity and Specificity Results</td><td><u>Samples</u></td><td><u>Sensitivity (95% CI)*</u></td><td><u>Specificity (95% CI)*</u></td><td><u>Comments</u></td></tr>
<tr><td>0% and wrong API</td><td>100 (88.8-100)</td><td rowspan="3">100 (83.2-100)</td><td rowspan="3">PADs are not designed to test samples with lower API amounts than stated.</td></tr>
<tr><td>50% and 80% API†</td><td>0 (0-11.6)</td></tr>
<tr><td>All poor-quality samples</td><td>50.8 (37.7-63.9)</td></tr>
<tr><td>Strengths and Limitations</td><td colspan="4">Strengths:
-High accuracy to identify samples with no or wrong API.

Limitations:
-Limited performance to identify medicines with reduced amount of API.†</td></tr>
<tr><td>User Satisfaction</td><td colspan="4">Plus:
Step-by-step protocols; few consumables needed; fast and easy experiments; electricity not required; computer not needed.

Minus:
Color changes can be difficult to interpret sometimes; Application of the API to the PAD can be cumbersome if not crushed finely enough into a powder; product has a shelf life</td></tr>
<tr><td>Comparative Evaluation</td><td colspan="4">-No significant differences in sensitivity compared to other devices to identify 0% and wrong API samples and higher specificity than the C-Vue liquid chromatograph.
-Longer total time per sample compared to other devices, except Minilab TLC kit (significantly shorter total time per sample compared to Minilab).
-Several samples can be run at the same time on multiple PADs.</td></tr>
</table>

* Sensitivity and specificity for quality assessment of the dosage unit not through the packaging

† The PADs used in this study were designed to detect the presence of the API (and of some potential wrong API), but not to quantitate the amount of API, i.e. substandard medicines (both containing low and high API) cannot reliably be tested.
