# Supplementary material for: Laboratory evaluation of twelve portable devices for medicine quality screening
Source: PLoS Negl Trop Dis. 2021 Sep 30;15(9):e0009360. doi: 10.1371/journal.pntd.0009360 (PMC8483346; doi:10.1371/journal.pntd.0009360)
Supplement: S15 Appendix — (PDF) [file pntd.0009360.s015.pdf]

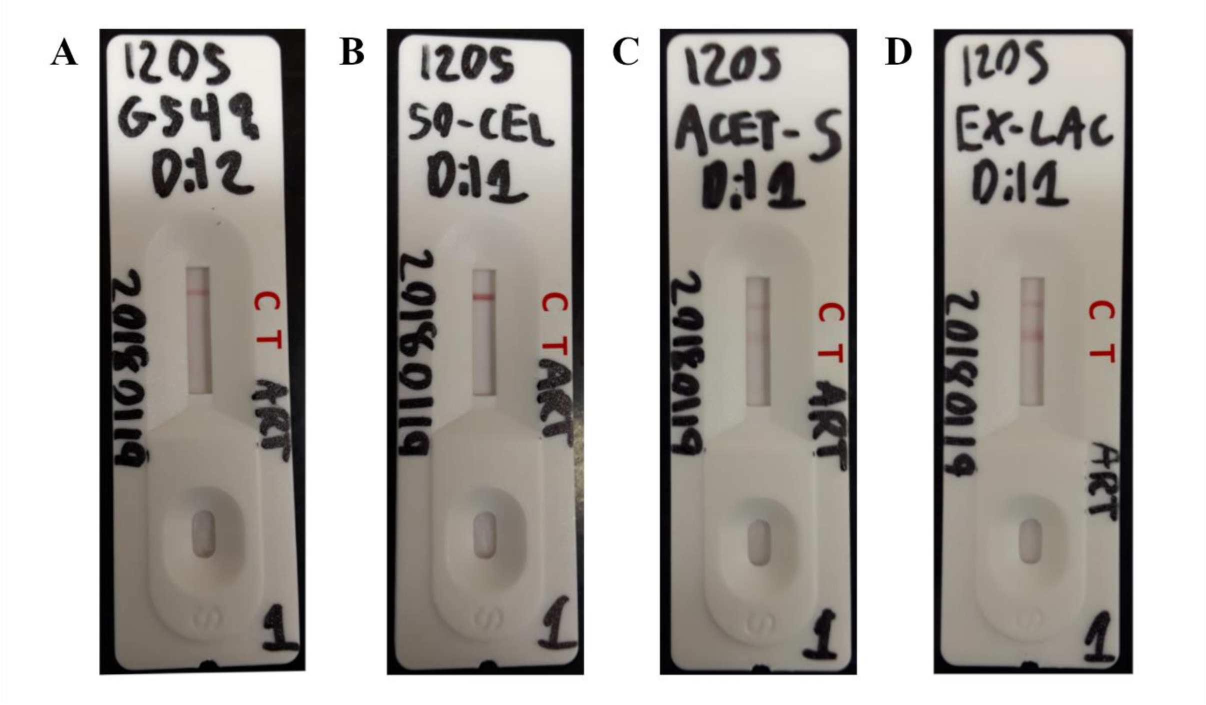

S15 Appendix. RDT analysis.
Images for processed rapid diagnostic tests (RDTs). **(A)** Testing of a field-collected genuine intravenous artesunate sample. It was classified as “pass” by the investigator because the control line (“C”) was present while the test line (“T”) was absent. **(B)** Testing of a simulated sample containing 50% of the recommended dosage of artesunate with cellulose as the excipient. It was classified as “pass” by the investigator because the control line (“C”) was present while the test line (“T”) was absent. **(C)** Testing of a simulated sample containing acetaminophen and starch but none of the expected correct API (artesunate). This sample was classified as “fail” because of the presence of the control line and test line despite some fading and blurriness in both lines. **(D)** Testing of a simulated sample containing only lactose but not the expected API (artesunate). It was classified as “fail” because of the presence of the control line and test line despite some fading and blurriness in both lines. Note that in all the images, the “C” and “T” labels were enhanced in red by photo editing because they were difficult to see without edits. This difficulty is because both the “C” and “T” labels are raised from the plastic case and the same color as the cartridge.
