# Supplementary material for: Laboratory evaluation of twelve portable devices for medicine quality screening
Source: PLoS Negl Trop Dis. 2021 Sep 30;15(9):e0009360. doi: 10.1371/journal.pntd.0009360 (PMC8483346; doi:10.1371/journal.pntd.0009360)
Supplement: S16 Appendix — (PDF) [file pntd.0009360.s016.pdf]

S16 Appendix. Rapid Diagnostic Test (RDT) results.

Table S16 A. Rapid Diagnostic Test (RDT) detailed performance breakdown. 1
Table S16 B. Rapid Diagnostic Tests (RDTs) single use device evaluation summary..................... 2

Table S16 A. Rapid Diagnostic Test (RDT) detailed performance breakdown.

	**Good quality samples available for specificity calculation: *n*=3**			
	**0% and wrong API samples (n=12)**		**50% and 80% API samples (n=12)**	**All poor quality samples (n=24)**
**Samples**	**Sensitivity (95% CI)**	**Specificity (95% CI)**	**Sensitivity (95% CI)**	**Sensitivity (95% CI)**
Total, not through packaging (n=27)	100 (73.5-100)	100 (29.2-100)	16.7 (2.1-48.4)	58.3 (36.6-77.9)
Antimalarials (n=27)	100 (73.5-100)	100 (29.2-100)	16.7 (2.1-48.4)	58.3 (36.6-77.9)
ART (n=14)	100 (54.1-100)	100 (15.8-100)	33.3 (4.3-77.7)	66.7 (34.9-90.1)
Dihydroartemisinin (n=13)	100 (54.1-100)	100 (2.5-100)	0 (0-45.9)	50 (21.1-78.9)

Table S16 B. Rapid Diagnostic Tests (RDTs) single use device evaluation summary.

<table>
<tr><td rowspan="4">Sensitivity and Specificity Results</td><td>Samples</td><td>Sensitivity (95% CI)*</td><td>Specificity (95% CI)*</td><td>Comments</td></tr>
<tr><td>0% and wrong API</td><td>100 (73.5-100)</td><td rowspan="3">100 (29.2-100)</td><td rowspan="3">N/A</td></tr>
<tr><td>50% and 80% API</td><td>16.7 (2.1-48.4)</td></tr>
<tr><td>All poor quality samples</td><td>58.3 (36.6-77.9)</td></tr>
<tr><td>Strengths and Limitations</td><td colspan="4">Strengths:
-High accuracy to identify samples with no or wrong API.

Limitations:
-None of 80% API samples correctly identified as “fail”.†
-One out of three 50% ART and all 50% dihydroartemisinin samples incorrectly identified as “pass”.</td></tr>
<tr><td>User Satisfaction</td><td colspan="4">Plus:
Easy-to-use (same as malaria rapid diagnostic); integrated quality control (control line); electricity not required; computer not needed.

Minus:
Interpretation can be counterintuitive (lane appearing at test line means sample fails); destroys sample; sample preparation needed; two tests (one at low and one at high concentration) to determine the sample as “no API” or as “API present but lower amount than stated”; does not quantitate API; colors of test results can be inconsistent (light pink to red), which can be confusing to users; co-formulated ACT cannot be fully characterized (only artemisinin derivatives can be tested); has a shelf-life; chemicals required.</td></tr>
<tr><td>Comparative Evaluation</td><td colspan="4">No significant differences in sensitivity compared to other devices to identify 0% and wrong API samples.*</td></tr>
</table>

* Among the seven APIs included in this work the RDTs only had the ability to test artesunate and DHAP samples (i.e. only artemisinin derivatives are tested). No comparisons of performance with the C-Vue could thus be conducted (ART and DHAP were not tested with the C-Vue chromatograph
