# Supplementary material for: Laboratory evaluation of twelve portable devices for medicine quality screening
Source: PLoS Negl Trop Dis. 2021 Sep 30;15(9):e0009360. doi: 10.1371/journal.pntd.0009360 (PMC8483346; doi:10.1371/journal.pntd.0009360)
Supplement: S17 Appendix — (PDF) [file pntd.0009360.s017.pdf]

S17 Appendix. 4500a single reflection FTIR spectrometer results

Table S17 A. 4500a single reflection FTIR spectrometer detailed performance breakdown. 1
Table S17 B. 4500a single reflection FTIR spectrometer laboratory evaluation summary. 2

Table S17 A. 4500a single reflection FTIR spectrometer detailed performance breakdown.

	**Good-quality samples available for specificity calculation: *n*=22**			
	**0% and wrong API samples (n=53)**		**50% and 80% API samples (n=42)**	**All poor quality samples (n=95)**
**Samples**	**Sensitivity (95% CI)**	**Specificity (95% CI)**	**Sensitivity (95% CI)**	**Sensitivity (95% CI)**
Total, not through packaging (n=119)	100 (93.3-100)	100 (85.8-100)	28.6 (15.7-44.6)	68.4 (58.1-77.6)
Antimalarials (n=51)	100 (87.7-100)	100 (47.8-100)	33.3 (13.3-59)	73.9 (58.9-85.7)
AL (n=24)	100 (79.4-100)	100 (15.8-100)	33.3 (4.3-77.7)	81.8 (59.7-94.8)
ART (n=14)	100 (54.1-100)	100 (15.8-100)	33.3 (4.3-77.7)	66.7 (34.9-90.1)
DHAP (n=13)	100 (54.1-100)	100 (2.5-100)	33.3 (4.3-77.7)	66.7 (34.9-90.1)
Antibiotics (n=68)	100 (86.3-100)	100 (82.4-100)	25 (9.8-46.7)	63.3 (48.3-76.6)
ACA (n=15)	100 (54.1-100)	100 (29.2-100)	33.3 (4.3-77.7)	66.7 (34.9-90.1)
AZITH (n=16)	100 (54.1-100)	100 (39.8-100)	0 (0-45.9)	50 (21.1-78.9)
OFLO (n=19)	100 (54.1-100)	100 (59.0-100)	33.3 (4.3-77.7)	66.7 (34.9-90.1)
SMTM (n=18)	100 (59.0-100)	100 (47.8-100)	33.3 (4.3-77.7)	69.2 (38.6-90.9)

Table S17 B. 4500a single reflection FTIR spectrometer laboratory evaluation summary.

<table>
<tr><td rowspan="4">Sensitivity and Specificity Results</td><td><u>Samples</u></td><td><u>Sensitivity (95% CI)</u></td><td><u>Specificity (95% CI)</u></td><td><u>Comments</u></td></tr>
<tr><td>0% and wrong API</td><td>100% (93.3-100)</td><td rowspan="3">100% (85.8-100)</td><td rowspan="3">Developing API-specific algorithms could improve device performance to identify poor quality medicines with low API.</td></tr>
<tr><td>50% and 80% API†</td><td>28.6% (15.7-44.6)</td></tr>
<tr><td>All poor quality samples</td><td>68.4% (58.1-77.6)</td></tr>
<tr><td>Strengths and Limitations</td><td colspan="4">Strengths:
-High accuracy to identify samples with no or wrong API.

Limitations:
-None of 80% API medicines samples correctly identified as “fail”.†
-Almost half of 50% API samples not correctly identified.†
-All AZITH 50% samples and all substandard containing cellulose were incorrectly identified.†</td></tr>
<tr><td>User Satisfaction</td><td colspan="4">Plus:
Step by step protocols available; results easy to interpret and extract; results trusted by medicine inspectors; table of matches with correlation values appreciated; no need to select reference library; useful for identifying the contents of medicines of unknown identity.

Minus:
Reference library creation needed; computer required for sample testing; occasional freezing of the software; cleaning sampling window time consuming; device felt to be too big and heavy; large number of steps required to perform analysis; destroys sample; errors in naming of samples could affect traceability.</td></tr>
<tr><td>Comparative Evaluation</td><td colspan="4">No significant differences in sensitivity compared to other devices to identify 0% and wrong API samples. Higher specificity than the C-Vue liquid chromatograph.
Longer total time per sample compared to other spectrometers.
Shorter time per sample compared to PADs and the Minilab TLC kit.</td></tr>
</table>

† Algorithms should be developed on an API basis to enhance detection of lower API samples (this was not performed in the present study, therefore these results should be interpreted with caution).
