# Supplementary material for: Laboratory evaluation of twelve portable devices for medicine quality screening
Source: PLoS Negl Trop Dis. 2021 Sep 30;15(9):e0009360. doi: 10.1371/journal.pntd.0009360 (PMC8483346; doi:10.1371/journal.pntd.0009360)
Supplement: S18 Appendix — (PDF) [file pntd.0009360.s018.pdf]

S18 Appendix. Neospectra 2.5 NIR spectrometer results

Table S18 A. Neospectra 2.5 NIR spectrometer detailed performance breakdown. 1

Table S18 B. Neospectra 2.5 NIR spectrometer with sampling probe and light source evaluation summary. 2

Table S18 A. Neospectra 2.5 NIR spectrometer detailed performance breakdown.

	Good-quality samples available for specificity calculation: *n*=22			
	0% and wrong API samples (n=47)		50% and 80% API samples (n=36)	All poor quality samples (n=83)
Samples	Sensitivity (95% CI)	Specificity (95% CI)	Sensitivity (95% CI)	Sensitivity (95% CI)
Total, not through packaging (n=105)	100 (92.5-100)	100 (84.6-100)	5.6 (0.7-18.7)	59 (47.7-69.7)
Antimalarials (n=37)	100 (84.6-100)	100 (29.2-100)	16.7 (2.1-48.4)	70.6 (52.5-84.9)
AL (n=24)	100 (79.4-100)	100 (15.8-100)	0 (0-45.9)	72.7 (49.8-89.3)
ART (n=0)*	N/A	N/A	N/A	N/A
DHAP (n=13)	100 (54.1-100)	100 (2.5-100)	33.3 (4.3-77.7)	66.7 (34.9-90.1)
Antibiotics (n=68)	100 (86.3-100)	100 (82.4-100)	0 (0-14.2)	51 (36.3-65.6)
ACA (n=15)	100 (54.1-100)	100 (29.2-100)	0 (0-45.9)	50 (21.1-78.9)
AZITH (n=16)	100 (54.1-100)	100 (39.8-100)	0 (0-45.9)	50 (21.1-78.9)
OFLO (n=19)	100 (54.1-100)	100 (59-100)	0 (0-45.9)	50 (21.1-78.9)
SMTM (n=18)	100 (59-100)	100 (47.8-100)	0 (0-45.9)	53.8 (25.1-80.8)

	Good-quality samples available for specificity calculation: *n*=3			
	0% and wrong API samples (n=10)		50% and 80% API samples (n=0)	All poor quality samples (n=10)
Samples	Sensitivity (95% CI)	Specificity (95% CI)	Sensitivity (95% CI)	Sensitivity (95% CI)
Total, through packaging † ***(n=13)***	100 (69.2-100)	100 (29.2-100)	N/A	100 (69.2-100)

	Good quality samples available for specificity calculation: n=1			
	0% and wrong API samples (n=6)		50% and 80% API samples (n=6)	All poor quality samples (n=12)
Samples	Sensitivity (95% CI)	Specificity (95% CI)	Sensitivity (95% CI)	Sensitivity (95% CI)
Total through replacement packaging ‡ ***(n=13)***	100 (54.1-100)	100 (2.5-100)	50.0 (11.8-88.2)	75.0 (42.8-94.5)

* Not applicable – insufficient amount of powder (60 mg) to be tested directly with the device - ART samples were thus scanned through replacement packaging;

† Packaging available with medicine (blister or glass vial for one field collected ART sample).

‡ Insufficient genuine parenteral artesunate vials were available for testing and therefore borosilicate replacement vials were used.

Table S18 B. Neospectra 2.5 NIR spectrometer with sampling probe and light source evaluation summary.

<table>
<tr><td rowspan="4">Sensitivity and Specificity Results</td><td><u>Samples</u></td><td><u>Sensitivity (95% CI)*</u></td><td><u>Specificity (95% CI)*</u></td><td><u>Comments</u></td></tr>
<tr><td>0% and wrong API</td><td>100 (92.5-100)</td><td rowspan="3">100 (84.6-100)</td><td rowspan="3">Developing library functionality could improve analysis times and sensitivity to identify poor quality medicines with low API.</td></tr>
<tr><td>50% and 80% API†</td><td>5.6 (0.7-18.7)</td></tr>
<tr><td>All poor quality samples</td><td>59 (47.7-69.7)</td></tr>
<tr><td>Strengths and Limitations</td><td colspan="4">Strengths:
-High accuracy in identifying samples with no or wrong API (both directly and through packaging).
-Good performance through packaging for 0% and wrong API identification.

Limitations:
-Limited performance to identify 50% and 80% API samples (except all three ART and two out of three DHAP samples). †
Potentially improved identification with development of algorithms (vs. visual inspection of spectra).</td></tr>
<tr><td>User Satisfaction</td><td colspan="4">Plus:
Easy to set-up; small size; highly configurable.

Minus:
No ability to computationally compare spectra; reference library creation needed; computer required.</td></tr>
<tr><td>Comparative Evaluation</td><td colspan="4">No significant differences in sensitivity compared to other devices to identify 0% and wrong API samples and higher specificity than the C-Vue liquid chromatograph.</td></tr>
</table>

* Sensitivity and specificity for quality assessment of the dosage unit not through the packaging.

† Algorithms should be developed on an API basis to enhance detection of lower API samples (this was not performed in the present study, therefore these results should be interpreted with caution).
