# Supplementary material for: Laboratory evaluation of twelve portable devices for medicine quality screening
Source: PLoS Negl Trop Dis. 2021 Sep 30;15(9):e0009360. doi: 10.1371/journal.pntd.0009360 (PMC8483346; doi:10.1371/journal.pntd.0009360)
Supplement: S19 Appendix — (PDF) [file pntd.0009360.s019.pdf]

S19 Appendix. NIR-S-G1 spectrometer results

Table S19 A. NIR-S-G1 spectrometer detailed performance breakdown. 1

Table S19 B. NIR-S-G1 spectrometer evaluation summary. 2

Table S19 A. NIR-S-G1 spectrometer detailed performance breakdown.

	**Good-quality samples available for specificity calculation: n=22**			
	**0% and wrong API samples (n=47)**		**50% and 80% API samples (n=36)**	**All poor quality samples (n=83)**
**Samples**	**Sensitivity (95% CI)**	**Specificity (95% CI)**	**Sensitivity (95% CI)**	**Sensitivity (95% CI)**
Total, not through packaging (n=105)	91.5 (79.6-97.6)	100 (84.6-100)	30.6 (16.3-48.1)	65.1 (53.8-75.2)
Antimalarials (n=37)	95.5 (77.2-99.9)	100 (29.2-100)	33.3 (9.9-65.1)	73.5 (55.6-87.1)
AL (n=24)	100 (79.4-100)	100 (15.8-100)	33.3 (4.3-77.7)	81.8 (59.7-94.8)
ART (n=0)*	N/A	N/A	N/A	N/A
DHAP (n=13)	83.3 (35.9-99.6)	100 (2.5-100)	33.3 (4.3-77.7)	58.3 (27.7-84.8)
Antibiotics (n=68)	88 (68.8-97.5)	100 (82.4-100)	29.2 (12.6-51.1)	59.2 (44.2-73)
ACA (n=15)	100 (54.1-100)	100 (29.2-100)	33.3 (4.3-77.7)	66.7 (34.9-90.1)
AZITH (n=16)	100 (54.1-100)	100 (39.8-100)	0 (0-45.9)	50 (21.1-78.9)
OFLO (n=19)	50 (11.8-88.2)	100 (59-100)	0 (0-45.9)	25 (5.5-57.2)
SMTM (n=18)	100 (59-100)	100 (47.8-100)	83.3 (35.9-99.6)	92.3 (64-99.8)

	**Good-quality samples available for specificity calculation: *n*=3**			
	**0% and wrong API samples (n=10)**		**50% and 80% API samples (n=0)**	**All poor quality samples (n=10)**
**Samples**	**Sensitivity (95% CI)**	**Specificity (95% CI)**	**Sensitivity (95% CI)**	**Sensitivity (95% CI)**
Total, through packaging (n=13)†	100 (69.2-100)	100 (29.2-100)	N/A	100 (69.2-100)

	**Good quality samples available for specificity calculation: *n*=1**			
	**0% and wrong API samples (n=6)**		**50% and 80% API samples (n=6)**	**All poor quality samples (n=12)**
**Samples**	**Sensitivity (95% CI)**	**Specificity (95% CI)**	**Sensitivity (95% CI)**	**Sensitivity (95% CI)**
Total, through replacement packaging (n=13)‡	100 (54.1-100)	100 (2.5-100)	50 (11.8-88.2)	75.0 (42.8-94.5)

* Not applicable - powder cannot be tested with the device - ART samples were thus scanned through packaging.
† Packaging available with medicine (blister or glass vial for one field collected ART sample).
‡ Insufficient genuine parenteral artesunate vials were available for testing and therefore borosilicate replacement vials were used.

Table S19 B. NIR-S-G1 spectrometer evaluation summary.

<table>
<tr><td rowspan="4">Sensitivity and Specificity Results</td><td><u>Samples</u></td><td><u>Sensitivity (95% CI)*</u></td><td><u>Specificity (95% CI)*</u></td><td><u>Comments</u></td></tr>
<tr><td>0% and wrong API</td><td>93.1 (86.6-99.6)</td><td rowspan="3">100 (100-100)</td><td rowspan="3">Developing API-specific algorithms could improve device performance to identify poor quality medicines with low API.</td></tr>
<tr><td>50% and 80% API†</td><td>28.6 (14.9-42.3)</td></tr>
<tr><td>All poor quality samples</td><td>66 (56.7-75.3)</td></tr>
<tr><td>Strengths and Limitations</td><td colspan="4">Strengths:
-High sensitivity to identify samples with no or wrong API.
-100% and 80% accuracies to identify 50% API and 80% API simulated medicines of SMTM, respectively.
-Good performance through packaging for 0% and wrong API identification.

Limitations:
-No API falsified OFLO mis-identified.
-Limited performance to identify medicines with reduced amount of API.†
-Issue with either the generated OFLO library, or inherent issue of the device.</td></tr>
<tr><td>User Satisfaction</td><td colspan="4">Plus:
Easy to use (smartphone application greatly appreciated); fast, small and light; computer not needed; averaging spectra for reference library creation possible to consider variability between batches or within batches.

Minus:
Reference library creation needed; reference libraries cannot be made by users at the time of testing; lack of local capability to update reference libraries; lack of ability to input identification information to the spectra files (sample details), limiting data traceability;</td></tr>
<tr><td>Comparative Evaluation</td><td colspan="4">-No significant differences of sensitivity compared to other devices to identify 0% and wrong API samples and higher specificity than the C-Vue liquid chromatograph.
-Fastest total time per sample.</td></tr>
</table>

* Sensitivity and specificity for quality assessment of the dosage unit not through the packaging.

† Algorithms should be developed on an API basis to enhance detection of lower API samples (this was not performed in the present study, therefore these results should be interpreted with caution).
