# Supplementary material for: Laboratory evaluation of twelve portable devices for medicine quality screening
Source: PLoS Negl Trop Dis. 2021 Sep 30;15(9):e0009360. doi: 10.1371/journal.pntd.0009360 (PMC8483346; doi:10.1371/journal.pntd.0009360)
Supplement: S20 Appendix — (PDF) [file pntd.0009360.s020.pdf]

S20 Appendix. MicroPHAZIR RX spectrometer results

Table S20 A. MicroPHAZIR RX spectrometer detailed performance breakdown........................... 1
Table S20 B. MicroPHAZIR RX spectrometer evaluation summary... 2

Table S20 A. MicroPHAZIR RX spectrometer detailed performance breakdown.

	**Good-quality samples available for specificity calculation: n=22**			
	**0% and wrong API samples (n=47)**		**50% and 80% API samples (n=36)**	**All poor quality samples (n=83)**
**Samples**	**Sensitivity (95% CI)**	**Specificity (95% CI)**	**Sensitivity (95% CI)**	**Sensitivity (95% CI)**
Total, not through packaging (n=105)	100 (92.5-100)	100 (84.6-100)	50 (32.9-67.1)	78.3 (67.9-86.6)
Antimalarials (n=37)	100 (84.6-100)	100 (29.2-100)	50 (21.1-78.9)	82.4 (65.5-93.2)
AL (n=24)	100 (79.4-100)	100 (15.8-100)	50 (11.8-88.2)	86.4 (65.1-97.1)
ART (n=0)	N/A	N/A	N/A	N/A
DHAP (n=13)	100 (54.1-100)	100 (2.5-100)	50 (11.8-88.2)	75 (42.8-94.5)
Antibiotics (n=68)	100 (86.3-100)	100 (82.4-100)	50 (29.1-70.9)	75.5 (61.1-86.7)
ACA (n=15)	100 (54.1-100)	100 (29.2-100)	50 (11.8-88.2)	75 (42.8-94.5)
AZITH (n=16)	100 (54.1-100)	100 (39.8-100)	50 (11.8-88.2)	75 (42.8-94.5)
OFLO (n=19)	100 (54.1-100)	100 (59-100)	50 (11.8-88.2)	75 (42.8-94.5)
SMTM (n=18)	100 (59-100)	100 (47.8-100)	50 (11.8-88.2)	76.9 (46.2-95)

	**Good-quality samples available for specificity calculation: n=3**			
	**0% API and wrong API samples (n=10)**		**50% and 80% API samples (n=0)**	**All poor quality samples (n=10)**
**Samples**	**Sensitivity (95% CI)**	**Specificity (95% CI)**	**Sensitivity (95% CI)**	**Sensitivity (95% CI)**
Total, through medicine packaging (n=13)†	100 (69.2-100)	100 (29.2-100)	N/A	**N/A**

	**Good-quality samples available for specificity calculation: *n*=1**			
	**0% and wrong API samples (n=6)**		**50% and 80% API samples (n=6)**	**All poor quality samples (n=12)**
**Samples**	**Sensitivity (95% CI)**	**Specificity (95% CI)**	**Sensitivity (95% CI)**	**Sensitivity (95% CI)**
Total through replacement packaging (n=13)‡	100 (54.1-100)	100 (2.5-100)	66.7 (22.3-95.7)	83.3 (51.6-97.9)

† Packaging available with medicine (blister or glass vial for one field collected ART sample);
‡ Insufficient genuine parenteral artesunate vials were available for testing and therefore borosilicate replacement vials were used.

Table S20 B. MicroPHAZIR RX spectrometer evaluation summary.

<table>
<tr><td rowspan="4">Sensitivity and Specificity Results</td><td><u>Samples</u></td><td><u>Sensitivity (95% CI)*</u></td><td><u>Specificity (95% CI)*</u></td><td><u>Comments</u></td></tr>
<tr><td>0% and wrong API</td><td>100% (92.5-100%)</td><td rowspan="3">100 (84.6-100)</td><td rowspan="3">Developing API-specific algorithms could improve device performance to identify poor quality medicines with low API.</td></tr>
<tr><td>50% and 80% API[†]</td><td>50.0% (32.9-67.1%)</td></tr>
<tr><td>All poor-quality samples</td><td>78.3% (67.9-86.6%)</td></tr>
<tr><td>Strengths and Limitations</td><td colspan="4">Strengths:
-High accuracy in identifying samples with no or wrong API.
-Good performance through packaging for 0% and wrong API identification.
-Good sensitivity to identify 50% API samples.[†]

Limitations:
- Low sensitivity to identify 80% API samples.</td></tr>
<tr><td>User Satisfaction</td><td colspan="4">Plus:
Easy to use; results trusted by medicine inspectors; averaging spectra for reference library creation possible to consider variability between batches or within batches; barcode reader to 1) enhance traceability, 2) reduce analysis time spent entering sample details; initial instrument set-up straightforward; sample window indicator helpful and provides additional confidence in results; does not destroy sample; computer not needed when testing.

Minus:
Reference library creation needed; heavy device; buttons hard to press; calibration and set-up of the device relatively prolonged; selecting the wrong initial reference library to compare subject to user errors; small tablets hard to scan; processing of reference libraries creation and updating not straightforward.</td></tr>
<tr><td>Comparative Evaluation</td><td colspan="4">-No significant differences in sensitivity compared to other devices to identify 0% and wrong API samples and higher specificity than the C-Vue.
- Faster total time per sample compared to other devices, except the NIRscan spectrometer (the device takes a longer time per sample than the NIRscan).</td></tr>
</table>

* Sensitivity and specificity for quality assessment of the dosage unit not through the packaging.

[†] Algorithms should be developed on an API basis to enhance detection of lower API samples (this was not performed in the present study, therefore these results should be interpreted with caution).
