# Supplementary material for: Laboratory evaluation of twelve portable devices for medicine quality screening
Source: PLoS Negl Trop Dis. 2021 Sep 30;15(9):e0009360. doi: 10.1371/journal.pntd.0009360 (PMC8483346; doi:10.1371/journal.pntd.0009360)
Supplement: S21 Appendix — (PDF) [file pntd.0009360.s021.pdf]

S21 Appendix. Progeny Raman spectrometer results

Table S21 A. Progeny Raman spectrometer detailed performance breakdown................................ 1

Table S21 B. Progeny Raman spectrometer evaluation summary.. 2

Table S21 A. Progeny Raman spectrometer detailed performance breakdown.

	**Good-quality samples available for specificity calculation: n=22**			
	**0% and wrong API samples (n=47)**		**50% and 80% API samples (n=36)**	**All poor quality samples (n=83)**
**Samples**	**Sensitivity (95% CI)**	**Specificity (95% CI)**	**Sensitivity (95% CI)**	**Sensitivity (95% CI)**
Total, not through packaging (n=105)	100 (92.5-100)	95.5 (77.2-99.9)	16.7 (6.4-32.8)	63.9 (52.6-74.1)
Antimalarials (n=37)	100 (84.6-100)	100 (29.2-100)	8.3 (0.2-38.5)	67.6 (49.5-82.6)
AL (n=24)	100 (79.4-100)	100 (15.8-100)	0 (0-45.9)	72.7 (49.8-89.3)
ART (n=0)*	N/A	N/A	N/A	N/A
DHAP (n=13)	100 (54.1-100)	100 (2.5-100)	16.7 (0.4-64.1)	58.3 (27.7-84.8)
Antibiotics (n=68)	100 (86.3-100)	94.7 (74-99.9)	20.8 (7.1-42.2)	61.2 (46.2-74.8)
ACA (n=15)	100 (54.1-100)	66.7 (9.4-99.2)	50 (11.8-88.2)	75 (42.8-94.5)
AZITH (n=16)	100 (54.1-100)	100 (39.8-100)	16.7 (0.4-64.1)	58.3 (27.7-84.8)
OFLO (n=19)	100 (54.1-100)	100 (59-100)	0 (0-45.9)	50 (21.1-78.9)
SMTM (n=18)	100 (59-100)	100 (47.8-100)	16.7 (0.4-64.1)	61.5 (31.6-86.1)

	**Good-quality samples available for specificity calculation: n=2**			
	**0%and wrong API samples (n=10)**		**50% and 80% API samples (n=0)**	**All poor quality samples (n=10)**
**Samples**	**Sensitivity (95% CI)**	**Specificity (95% CI)**	**Sensitivity (95% CI)**	**Sensitivity (95% CI)**
Total, through medicine packaging (n=12)†	100 (69.2-100)	100 (15.8-100)	N/A	100 (69.2-100)

	**Good quality samples available for specificity calculation: n=1**			
	**0% and wrong API samples (n=6)**		**50% and 80% API samples (n=6)**	**All poor quality samples (n=12)**
**Samples**	**Sensitivity (95% CI)**	**Specificity (95% CI)**	**Sensitivity (95% CI)**	**Sensitivity (95% CI)**
Total, through replacement packaging (n=13)‡	100 (54.1-100)	100 (2.5-100)	16.7 (0.4-64.1)	83.3 (51.6-97.9)

* Not applicable – insufficient amount of powder (60 mg) to be tested directly with the device - ART samples were thus scanned through replacement packaging;

† Packaging available with medicine (blister or glass vial for one field collected ART sample);

‡ Insufficient genuine parenteral artesunate vials were available for testing and therefore borosilicate replacement vials were used

.

Table S21 B. Progeny Raman spectrometer evaluation summary.

	Samples	*Sensitivity (95% CI)**	*Specificity (95% CI)**	*Comments*
**Sensitivity and Specificity Results**	0% and wrong API	100 (92.5-100)	95.5 (77.2-99.9)	Developing API-specific algorithms could improve device performance to identify poor quality medicines with low API.
	50% and 80% API†	16.7 (6.4-32.8)		
	All poor quality samples	63.9 (52.6-74.1)		
**Strengths and Limitations**	*Strengths:*<br>-High accuracy in identifying samples with no or wrong API.<br>-Good performance through packaging (except through glass vial for ART samples) for 0% and wrong API identification.<br><br>*Limitations:*<br>No 80% API samples identified as “fail”.<br>Poor sensitivity to identify 50% API samples (except ACA samples).<br>Issue to identify one brand of FC ACA (potential issue with coating).<br>False positives using the “Analyze” function were observed because of spectral similarities between brands of the same API.			
**User Satisfaction**	*Plus:*<br>Simple procedure for reference library creation; easy-to-use; large number of in-built reference libraries; easy interpretation (return of the closest match appreciated); computer not needed.<br><br>*Minus:*<br>averaging spectra for reference library creation to take into account variability inter-batch or of dosage units from same batches not possible (spectra individually added in the library); heavy weight; large width; touchscreen not very responsive increasing the time to record; different functions may be confusing for end users; tablet holder difficult to use for small tablets; daily calibration with chemicals (provided at purchase).			
**Comparative Evaluation**	Longest testing time per sample of all non-destructive spectrometers except the Truscan RM (users mentioned slowness); faster than 4500a FTIR spectrometer, PADs and Minilab TLC kit.			

* Sensitivity and specificity for quality assessment of the dosage unit not through the packaging.

† Algorithms should be developed on an API basis to enhance detection of lower API samples (this was not performed in the present study, therefore these results should be interpreted with caution).
