# Supplementary material for: Laboratory evaluation of twelve portable devices for medicine quality screening
Source: PLoS Negl Trop Dis. 2021 Sep 30;15(9):e0009360. doi: 10.1371/journal.pntd.0009360 (PMC8483346; doi:10.1371/journal.pntd.0009360)
Supplement: S22 Appendix — (PDF) [file pntd.0009360.s022.pdf]

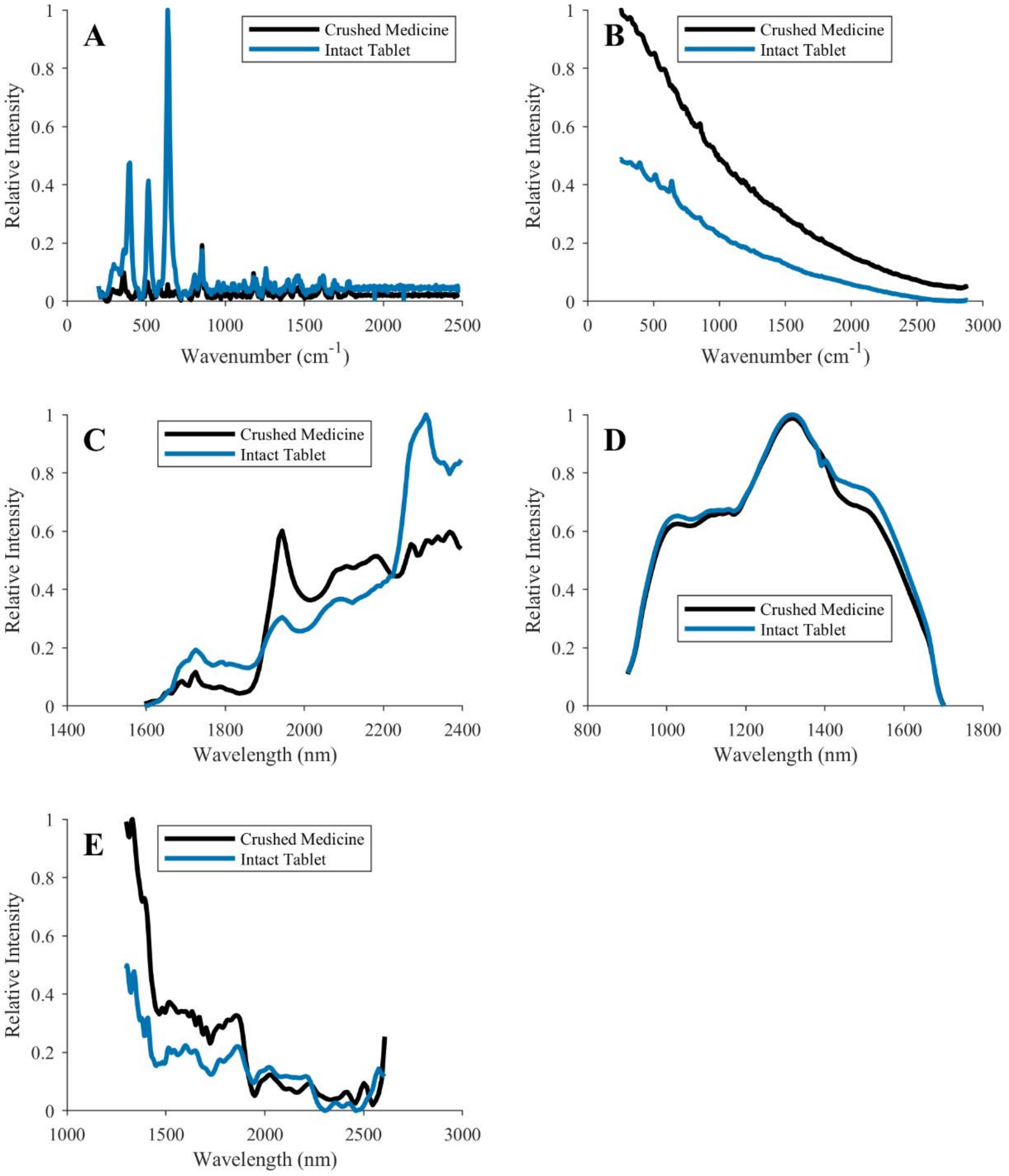

S22 Appendix. Intact vs. crushed ACA tablet analysis by Raman and NIR spectrometers. Spectral comparisons of a field collected sample of AMK 1000 mg (a co-formulation of amoxicillin and clavulanic acid) are shown with the **(A)** Progeny, **(B)** Truscan RM, **(C)** MicroPHAZIR RX, **(D)** NIR-S-G1, and **(E)** Neospectra 2.5 spectrometers. The spectra in blue are scans of an intact tablet of AMK 1000 mg. The spectra in black are scans of the powder from a crushed AMK 1000 mg tablet.
