# Supplementary material for: Laboratory evaluation of twelve portable devices for medicine quality screening
Source: PLoS Negl Trop Dis. 2021 Sep 30;15(9):e0009360. doi: 10.1371/journal.pntd.0009360 (PMC8483346; doi:10.1371/journal.pntd.0009360)
Supplement: S23 Appendix — (PDF) [file pntd.0009360.s023.pdf]

S23 Appendix. Truscan RM Raman spectrometer results

Table S23 A. Truscan RM Raman spectrometer detailed performance breakdown. 1

Table S23 B. Truscan RM Raman spectrometer evaluation summary. 2

Table S23 A. Truscan RM Raman spectrometer detailed performance breakdown.

	**Good quality samples available for specificity calculation: n=22**			
	**0% and wrong API samples (n=47)**		**50% and 80% API samples (n=36)**	**All poor quality samples (n=83)**
**Samples**	**Sensitivity (95% CI)**	**Specificity (95% CI)**	**Sensitivity (95% CI)**	**Sensitivity (95% CI)**
Total, not through packaging (n=105)	100 (92.5-100)	100 (84.6-100)	22.2 (10.1-39.2)	66.3 (55.1-76.3)
Antimalarials (n=37)	100 (84.6-100)	100 (29.2-100)	41.7 (15.2-72.3)	79.4 (62.1-91.3)
AL (n=24)	100 (79.4-100)	100 (15.8-100)	0 (0-45.9)	72.7 (49.8-89.3)
ART (n=0)*	N/A	N/A	N/A	N/A
DHAP (n=13)	100 (54.1-100)	100 (2.5-100)	83.3 (35.9-99.6)	91.7 (61.5-99.8)
Antibiotics (n=68)	100 (86.3-100)	100 (82.4-100)	12.5 (2.7-32.4)	57.1 (42.2-71.2)
ACA (n=15)	100 (54.1-100)	100 (29.2-100)	0 (0-45.9)	50 (21.1-78.9)
AZITH (n=16)	100 (54.1-100)	100 (39.8-100)	50 (11.8-88.2)	75 (42.8-94.5)
OFLO (n=19)	100 (54.1-100)	100 (59-100)	0 (0-45.9)	50 (21.1-78.9)
SMTM (n=18)	100 (59-100)	100 (47.8-100)	0 (0-45.9)	53.8 (25.1-80.8)

	**Good quality samples available for specificity calculation: n=2**			
	**0% and wrong API samples (n=10)**		**50% and 80% API samples (n=0)**	**All poor quality samples (n=10)**
**Samples**	**Sensitivity (95% CI)**	**Specificity (95% CI)**	**Sensitivity (95% CI)**	**Sensitivity (95% CI)**
Total, through packaging (n=12)†	100 (69.2-100)	100 (15.8-100)	N/A	100 (69.2-100)

	**Good quality samples available for specificity calculation: n=1**			
	**0% and wrong API samples (n=6)**		**50% and 80% API samples (n=6)**	**All poor quality samples (n=12)**
**Samples**	**Sensitivity (95% CI)**	**Specificity (95% CI)**	**Sensitivity (95% CI)**	**Sensitivity (95% CI)**
Total, through replacement packaging (n=13)‡	100 (54.1-100)	100 (2.5-100)	33.3 (4.3-77.7)	66.7 (34.9-90.1)

* Not applicable – insufficient amount of powder (60 mg) to be tested directly with the device - ART samples were thus scanned through replacement packaging;

† Packaging available with medicine (blister or glass vial for one field collected ART sample) ;

‡ Insufficient genuine parenteral artesunate vials were available for testing and therefore borosilicate replacement vials were used.

Table S23 B. Truscan RM Raman spectrometer evaluation summary.

<table>
<tr><td rowspan="4">Sensitivity and Specificity Results</td><td><u>Samples</u></td><td><u>Sensitivity (95% CI)*</u></td><td><u>Specificity (95% CI)*</u></td><td><u>Comments</u></td></tr>
<tr><td>0% and wrong API</td><td>100 (92.5-100)</td><td rowspan="3">100 (84.6-100)</td><td rowspan="3">Developing API-specific algorithms could improve device performance to identify poor quality medicines with low API.</td></tr>
<tr><td>50% and 80% API†</td><td>22.2 (10.1-39.2)</td></tr>
<tr><td>All poor quality samples</td><td>66.3 (55.1-76.3)</td></tr>
<tr><td>Strengths and Limitations</td><td colspan="4">Strengths:
-High accuracy in identifying samples with no or wrong API.
-Good performance through packaging (except through glass vial for ART samples) for 0% and wrong API identification.
-Good performance to identify 80% API DHAP samples.†

Limitations:
-Poor sensitivity to identify 50% API samples (except AZITH samples, 2 of the 3 DHAP and 2 of the 3 ART samples).†
-Poor sensitivity to identify 80% API (except DHAP samples).†</td></tr>
<tr><td>User Satisfaction</td><td colspan="4">Plus:
Several batches of the same reference sample can be added to the reference library to consider variability; easy to use for end user, step-by-step screen instructions; when sample fails to match the selected reference library spectrum, the whole library of spectra is searched by the device looking for the closest match; computer not needed for field-testing.

Minus:
Reference library creation cannot be accomplished to the device itself; initial set-up of master computer and software packages difficult, requiring IT skills; difficulties to scroll down with buttons when looking for the reference library; tablet holder not adapted to larger or smaller sized tablets;</td></tr>
<tr><td>Comparative Evaluation</td><td colspan="4">-No significant differences in sensitivity compared to other devices to identify 0% and wrong API samples; higher specificity than the C-Vue liquid chromatograph.
-Same total time per sample as Progeny spectrometer but slower than the NIRscan spectrometer (faster than 4500a FTIR spectrometer).</td></tr>
</table>

* Sensitivity and specificity for quality assessment of the dosage unit not through the packaging.

† Algorithms should be developed on an API basis to enhance detection of lower API samples (this was not performed in the present study; therefore, these results should be interpreted with caution)
