# Supplementary material for: Laboratory evaluation of twelve portable devices for medicine quality screening
Source: PLoS Negl Trop Dis. 2021 Sep 30;15(9):e0009360. doi: 10.1371/journal.pntd.0009360 (PMC8483346; doi:10.1371/journal.pntd.0009360)
Supplement: S24 Appendix — (PDF) [file pntd.0009360.s024.pdf]

S24 Appendix. C-Vue liquid chromatograph results

Table S24 A. C-Vue liquid chromatograph detailed performance breakdown................................. 1
Table S24 B. C-Vue liquid chromatograph evaluation summary... 2

Table S24 A. C-Vue liquid chromatograph detailed performance breakdown.

	**Good quality samples available for specificity calculation: n=22**			
	**0% and wrong API samples (n=19)**		**50% and 80% API samples (n=18)**	**All poor quality samples (n=37)**
**Samples**	**Sensitivity (95% CI)**	**Specificity (95% CI)**	**Sensitivity (95% CI)**	**Sensitivity (95% CI)**
Total, not through packaging (n=52)	100 (82.4-100)	60 (32.3-83.7)	100 (81.5-100)	100 (90.5-100)
Antimalarials (n=0)	N/A	N/A	N/A	N/A
AL (n=0)	N/A	N/A	N/A	N/A
ART (n=0)	N/A	N/A	N/A	N/A
DHAP (n=0)	N/A	N/A	N/A	N/A
Antibiotics (n=52)	100 (82.4-100)	60 (32.3-83.7)	100 (81.5-100)	100 (90.5-100)
ACA (n=15)	100 (54.1-100)	0 (0-70.8)	100 (54.1-100)	100 (73.5-100)
AZITH (n=0)	N/A	N/A	N/A	N/A
OFLO (n=19)	100 (54.1-100)	100 (59-100)	100 (54.1-100)	100 (73.5-100)
SMTM (n=18)	100 (59-100)	40 (5.3-85.3)	100 (54.1-100)	100 (75.3-100)

Table S24 B. C-Vue liquid chromatograph evaluation summary.

	Samples	*Sensitivity (95% CI)**	*Specificity (95% CI)**	*Comments*
**Sensitivity and Specificity Results**	*0% and wrong API*	100 (82.4-100)	60.0 (32.3-83.7)	Performance may be affected by sample extraction.
	50% and 80% API	100 (81.5-100)		
	All poor quality samples	100 (90.5-100)		
**Strengths and Limitations**	*Strengths:*<br>-High accuracy in identifying samples with no or wrong API.<br>-Correct identification of all 50% and 80% API medicines, with quantitation of API.<br><br>*Limitations:*<br>-Limited performance to identify genuine samples of co-formulated medicines.			
**User Satisfaction**	*Plus:*<br>Intuitive system for experienced analysts; no reference library creation required.<br><br>*Minus:*<br>Intensive operation and set-up; two computers required to run dual detector set-up; destroys sample; chemicals required; requires experienced end-users.			
**Comparative Evaluation**	No significant differences in sensitivity compared to other devices to identify 0% and wrong API samples; lower specificity than all other devices*			

* Pair-wise comparisons with PharmaChk and RDT could not be performed, no overlapping API.
