# Supplementary material for: Laboratory evaluation of twelve portable devices for medicine quality screening
Source: PLoS Negl Trop Dis. 2021 Sep 30;15(9):e0009360. doi: 10.1371/journal.pntd.0009360 (PMC8483346; doi:10.1371/journal.pntd.0009360)
Supplement: S25 Appendix — (PDF) [file pntd.0009360.s025.pdf]

S25 Appendix. PharmaChk microfluidic device results

Table S25 A. PharmaChk microfluidic device detailed performance breakdown. 1

Table S25 B. PharmaChk microfluidic device evaluation summary. 2

Table S25 A. PharmaChk microfluidic device detailed performance breakdown.

	**Good quality samples available for specificity calculation: n=2**			
	**0% and wrong API samples (n=6)**		**50% and 80% API samples (n=6)**	**All poor quality samples (n=12)**
**Samples**	**Sensitivity (95% CI)**	**Specificity (95% CI)**	**Sensitivity (95% CI)**	**Sensitivity (95% CI)**
Total (n=14)	100.0 (54.1-100)	50.0 (1.3-98.7)	83.3 (35.9-99.6)	91.7 (61.5-99.8)

Table S25 B. PharmaChk microfluidic device evaluation summary.

	Samples	*Sensitivity (95% CI)**	*Specificity (95% CI)**	*Comments*
**Laboratory evaluation**	*0% and wrong API*	100.0 (54.1-100)	50.0 (1.3-98.7)	N/A
	50% and 80% API	83.3 (35.9-99.6)		
	All poor quality samples	91.7 (61.5-99.8)		
**Strengths and Limitations**	*Strengths:*<br>-High accuracy in identifying samples with no or wrong API.<br>-Correct identification of all 50% API medicines, and one of the three 80% API, with quantitation of API.<br><br>*Limitations:*<br>-One of the two simulated 100% API samples tested could not be identified as “pass”. The field collected genuine was correctly identified.			
**User satisfaction**	*Plus:*<br>Calibration reference samples run simultaneously with sample being tested; quantitation of APIs; instructions illustrated with photos.<br><br>*Minus:*<br>Destroys sample; sample preparation needed; computer needed; chemicals required; degradation of reagents over relatively short time;			
**Comparative Evaluation**	No significant differences in sensitivity and specificity compared to Minilab, RDTs and 4500a FTIR to identify 0% and wrong API samples.*			

* Among the seven APIs included in this work the PharmaChk only had the ability to test artesunate samples. The only comparison that could be conducted for the PharmaChk performance with testing artesunate powder outside of packaging was with the 4500a FTIR, the Minilab and the RDTs, limiting pair wise comparisons.
