# Supplementary material for: Laboratory evaluation of twelve portable devices for medicine quality screening
Source: PLoS Negl Trop Dis. 2021 Sep 30;15(9):e0009360. doi: 10.1371/journal.pntd.0009360 (PMC8483346; doi:10.1371/journal.pntd.0009360)
Supplement: S26 Appendix — (PDF) [file pntd.0009360.s026.pdf]

S26 Appendix. QDa mass spectrometer results

Table S26 A. QDa mass spectrometer detailed performance breakdown.. 1
Table S26 B. QDa mass spectrometer evaluation summary. 2

Table S26 A. QDa mass spectrometer detailed performance breakdown.

	**Good quality samples available for specificity calculation: n=24**			
	**0% and wrong API samples (n=19)**		**50% and 80% API samples (n=18)**	**All poor quality samples (n=37)**
**Samples**	**Sensitivity (95% CI)**	**Specificity (95% CI)**	**Sensitivity (95% CI)**	**Sensitivity (95% CI)**
Total, not through packaging (n=105)	100 (93.3-100)	91.7 (73.0-99.0)	100 (91.6-100)	100 (96.2-100)
Antimalarials (n=51)	100 (87.7-100)	80.0 (28.4-99.5)	100 (81.5-100)	100 (92.3-100)
AMLM (n=24)	100 (79.4-100)	100 (15.8-100)	100 (54.1-100)	100 (84.6-100)
ART (n=14)	100 (54.1-100)	50 (1.3-98.7)	100 (54.1-100)	100 (73.5-100)
DHAP (n=13)	100 (54.1-100)	100 (2.5-100)	100 (54.1-100)	100 (73.5-100)
Antibiotics (n=68)	100 (86.3-100)	94.7 (74-99.9)	100 (85.8-100)	100 (92.7-100)
ACA (n=15)	100 (54.1-100)	100 (29.2-100)	100 (54.1-100)	100 (73.5-100)
AZITH (n=16)	100 (54.1-100)	100 (39.8-100)	100 (54.1-100)	100 (73.5-100)
OFLO (n=19)	100 (54.1-100)	85.7 (42.1-99.6)	100 (54.1-100)	100 (73.5-100)
SMTM (n=18)	100 (59.0-100)	100 (47.8-100)	100 (54.1-100)	100 (75.3-100)

Table S26 B. QDa mass spectrometer evaluation summary.

<table>
<tr><td rowspan="4">Sensitivity and Specificity Results</td><td><u>Samples</u></td><td><u>Sensitivity (95% CI)*</u></td><td><u>Specificity (95% CI)*</u></td><td><u>Comments</u></td></tr>
<tr><td>0% and wrong API</td><td>100 (93.3-100)</td><td rowspan="3">91.7 (73.0-99.0)</td><td rowspan="3">N/A</td></tr>
<tr><td>50% and 80% API</td><td>100 (91.6-100)</td></tr>
<tr><td>All poor-quality samples</td><td>100 (96.2-100)</td></tr>
<tr><td>Strengths and Limitations</td><td colspan="4">Strengths:
-High accuracy in identifying samples with no or wrong API.
- Correct identification of all 50% and 80% API medicines, with possibility of quantitation of API.

Limitations:
-Many consumable requirements include the need for a nitrogen-gas source.
-Sample preparation required to obtain concentrations dilute enough to be within the quantitative range of the instrument.
-Higher complexity and higher cost instrument.</td></tr>
<tr><td>User Satisfaction</td><td colspan="4">Plus:
Highest sensitivity and specificity quantitative instrument tested with many other types of experiments possible.

Minus:
Intensive operation and set-up; chemicals and gases required; requires more experienced users.</td></tr>
<tr><td>Comparative Evaluation</td><td colspan="4">No significant differences in sensitivity compared to other devices to identify 0% and wrong API samples and lower specificity than all other devices except the C-Vue.</td></tr>
</table>

* Sensitivity and specificity for quality assessment of the dosage unit not through the packaging.
