# Supplementary material for: Laboratory evaluation of twelve portable devices for medicine quality screening
Source: PLoS Negl Trop Dis. 2021 Sep 30;15(9):e0009360. doi: 10.1371/journal.pntd.0009360 (PMC8483346; doi:10.1371/journal.pntd.0009360)
Supplement: S27 Appendix — (PDF) [file pntd.0009360.s027.pdf]

S27 Appendix. Pair-wise analysis

Table S27 A. Pair-wise sensitivity comparisons of the devices used to test 0% and wrong API samples outside of their packaging. 2

Table S27 B. Pair-wise specificity comparisons of the devices used to test genuine samples outside their packaging. 3

Table S27 C. Pair-wise sensitivity comparisons of the devices used to test 50 % and 80 % API samples outside their packaging. 4

Table S27 A. Pair-wise sensitivity comparisons of the devices used to test 0% and wrong API samples outside of their packaging. Sensitivities expressed as 95% CI, in grey. The p-value of the McNemar test (n=number of 0%/wrong API medicines assessed by both devices in the pair) is presented. The pairs for which a significant difference was observed are shown in orange, if any.

	4500a FTIR	C-Vue	MicroPHAZIR RX	Minilab	Neospectra 2.5	NIR-S-G1	PADs	PharmaChk	Progeny	QDa	TruScan RM	RDT
**4500a FTIR**	100 (93.3-100)											
**C-Vue**	1 (n=19)	100 (82.4-100)										
**MicroPHAZIR RX**	1 (n=47)	1 (n=19)	100 (92.5-100)									
**Minilab**	1 (n=53)	1 (n=19)	1 (n=47)	100 (93.3-100)								
**Neospectra 2.5**	1 (n=47)	1 (n=19)	1 (n=47)	1 (n=47)	100 (92.5-100)							
**NIR-S-G1**	0.1250 (n=47)	0.2500 (n=19)	0.1250 (n=47)	1 (n=47)	0.1250 (n=47)	91.5 (79.6-97.6)						
**PADs**	1 (n=31)	1 (n=19)	1 (n=31)	1 (n=31)	1 (n=31)	0.1250 (n=31)	100 (88.8-100)					
**PharmaChk**	1 (n=6)	N/A	N/A	1 (n=6)	N/A	N/A	N/A	100 (54.1-100)				
**Progeny**	1 (n=47)	1 (n=19)	1 (n=47)	1 (n=47)	1 (n=47)	0.1250 (n=47)	1 (n=31)	N/A	100 (92.5-100)			
**QDa**	1 (n=47)	1 (n=19)	1 (n=47)	1 (n=53)	1 (n=47)	0.1250 (n=47)	1 (n=31)	1 (n=6)	1 (n=47)	100 (93.3-100)		
**TruScan RM**	1 (n=47)	1 (n=19)	1 (n=47)	1 (n=47)	1 (n=47)	0.1250 (n=47)	1 (n=31)	N/A	1 (n=47)	1 (n=47)	100 (92.5-100)	
**RDT**	1 (n=12)	N/A	1 (n=6)	1 (n=6)	1 (n=6)	1 (n=6)	1 (n=6)	1 (n=6)	1 (n=6)	1 (n=6)	1 (n=6)	100 (73.5-100)

Table S27 B. Pair-wise specificity comparisons of the devices used to test genuine samples outside their packaging.

Specificities expressed as 95% CI, in grey. The p-value of the McNemar test (n=number of 0%/wrong API medicines assessed by both devices in the pair) is presented. The pairs for which a significant difference was observed are shown in orange, if any.

	**4500a FTIR**	**C-Vue**	**MicroPHAZIR RX**	**Minilab**	**Neospectra 2.5**	**NIR-S-G1**	**PADs**	**PharmaChk**	**Progeny**	**QDa**	**TruScan RM**	**RDT**
**4500a FTIR**	100 (85.8-100)											
**C-Vue**	**0.0313 (n=15)**	60 (32.3-83.7)										
**MicroPHAZIR RX**	1 (n=22)	**0.0313 (n=15)**	100 (84.6-100)									
**Minilab**	1 (n=24)	**0.0313 (n=15)**	1 (n=22)	100 (85.8-100)								
**Neospectra 2.5**	1 (n=22)	**0.0313 (n=15)**	1 (n=22)	1 (n=22)	100 (84.6-100)							
**NIR-S-G1**	1 (n=22)	**0.0313 (n=15)**	1 (n=22)	1 (n=22)	1 (n=22)	100 (84.6-100)						
**PADs**	1 (n=20)	**0.0313 (n=15)**	1 (n=20)	1 (n=20)	1 (n=20)	1 (n=20)	100 (83.2-100)					
**PharmaChk**	1 (n=2)	N/A	N/A	1 (n=2)	N/A	N/A	N/A	50 (1.3-98.7)				
**Progeny**	1 (n=22)	0.0625 (n=15)	1 (n=22)	1 (n=22)	1 (n=22)	1 (n=22)	1 (n=20)	N/A	95.5 (77.2-99.9)			
**QDa**	1 (n=22)	0.1250 (n=15)	1 (n=22)	0.50 (n=24)	1 (n=22)	1 (n=22)	1 (n=20)	1 (n=2)	1 (n=22)	91.7 (73.0-99.0)		
**TruScan RM**	1 (n=22)	**0.0313 (n=15)**	1 (n=22)	1 (n=22)	1 (n=22)	1 (n=22)	1 (n=20)	N/A	1 (n=22)	1 (n=22)	100 (84.6-100)	
**RDT**	1 (n=3)	N/A	1 (n=1)	1 (n=3)	1 (n=1)	1 (n=1)	1 (n=1)	1 (n=2)	1 (n=1)	1 (n=3)	1 (n=1)	100 (29.2-100)

Table S27 C. Pair-wise sensitivity comparisons of the devices used to test 50 % and 80 % API samples outside their packaging.
Sensitivities expressed as 95% CI, in grey. The p-value of the McNemar test (n=number of 0%/wrong API medicines assessed by both devices in the pair) is presented. The pairs for which a significant difference was observed are shown in orange, if any.

	4500a FTIR	C-Vue	MicroPHAZIR RX	Minilab	Neospectra 2.5	NIR-S-G1	PADs	PharmaChk	Progeny	QDa	TruScan RM	RDT
**4500a FTIR**	28.6 (15.7-44.6)											
**C-Vue**	**0.0005 (n=18)**	100 (81.5-100)										
**MicroPHAZIR RX**	**0.0078 (n=36)**	**0.0039 (n=18)**	50.0 (32.9-67.1)									
**Minilab**	**0.0002 (n=42)**	**0.0156 (n=18)**	0.6250 (n=36)	59.5 (43.3-74.4)								
**Neospectra 2.5**	**0.0215 (n=36)**	**<0.0001 (n=18)**	**<0.0001 (n=36)**	**<0.0001 (n=36)**	5.6 (0.7-18.7)							
**NIR-S-G1**	1 (n=36)	**0.0010 (n=18)**	0.0923 (n=36)	**0.0352 (n=36)**	**0.0117 (n=36)**	30.6 (16.3-48.1)						
**PADs**	**0.0078 (n=30)**	**<0.0001 (n=18)**	**0.0001 (n=30)**	**<0.0001 (n=30)**	0.5000 (n=30)	**0.0039 (n=30)**	0 (0-11.6)					
**PharmaChk**	0.2500 (n=6)	N/A	N/A	1 (n=6)	N/A	N/A	N/A	83.3 (35.9-99.6)				
**Progeny**	0.3438 (n=36)	**0.0001 (n=18)**	**0.0005 (n=36)**	**0.0001 (n=36)**	0.2188 (n=36)	0.1797 (n=36)	**0.0313 (n=30)**	N/A	16.7 (6.4-32.8)			
**QDa**	**<0.0001 (n=36)**	1 (n=18)	**<0.0001 (n=36)**	**<0.0001 (n=42)**	**<0.0001 (n=36)**	**<0.0001 (n=36)**	**<0.0001 (n=30)**	1 (n=6)	**<0.0001 (n=36)**	100 (91.6-100)		
**TruScan RM**	0.8036 (n=36)	**<0.0001 (n=18)**	**0.0213 (n=36)**	**0.0118 (n=36)**	**0.0313 (n=36)**	0.6072 (n=36)	**0.0078 (n=30)**	N/A	0.7539 (n=36)	**<0.0001 (n=36)**	22.2 (10.1-39.2)	
**RDT**	0.6250 (n=12)	N/A	0.2500 (n=6)	**0.0313 (n=12)**	0.5000 (n=6)	0.5000 (n=6)	1 (n=6)	0.25 (n=6)	1 (n=6)	**0.0020 (n=12)**	0.0625 (n=6)	16.7 (2.1-48.4)
