# Supplementary material for: Laboratory evaluation of twelve portable devices for medicine quality screening
Source: PLoS Negl Trop Dis. 2021 Sep 30;15(9):e0009360. doi: 10.1371/journal.pntd.0009360 (PMC8483346; doi:10.1371/journal.pntd.0009360)
Supplement: S28 Appendix — (PDF) [file pntd.0009360.s028.pdf]

S28 Appendix. Table describing the degree of difficulty to analyse other, less common, medicine formulations relative to the analysis of a tablet.

	Medicine formulation			
**Instrument**	**Capsule**	**Liquid (water based)**	**Powder**	**Creams/Gels**
4500a FTIR	Same	Higher	Easier	Higher
C-Vue	Same	Easier	Easier	Medium
MicroPHAZIR RX	Medium	Higher	Same	Higher
Minilab	Same	Easier	Easier	Medium
Neospectra 2.5	Medium	Higher	Same	Higher
NIRscan	Medium	Higher	Same	Higher
PADs	Same	Higher	Easier	Higher
PharmaChk	Same	Easier	Easier	Medium
Progeny	Medium	Higher	Same	Higher
RDTs	Same	Easier	Easier	Medium
Truscan RM	Medium	Higher	Same	Higher
QDa	Same	Easier	Easier	Medium
