# Supplementary material for: Laboratory evaluation of twelve portable devices for medicine quality screening
Source: PLoS Negl Trop Dis. 2021 Sep 30;15(9):e0009360. doi: 10.1371/journal.pntd.0009360 (PMC8483346; doi:10.1371/journal.pntd.0009360)
Supplement: S29 Appendix — (PDF) [file pntd.0009360.s029.pdf]

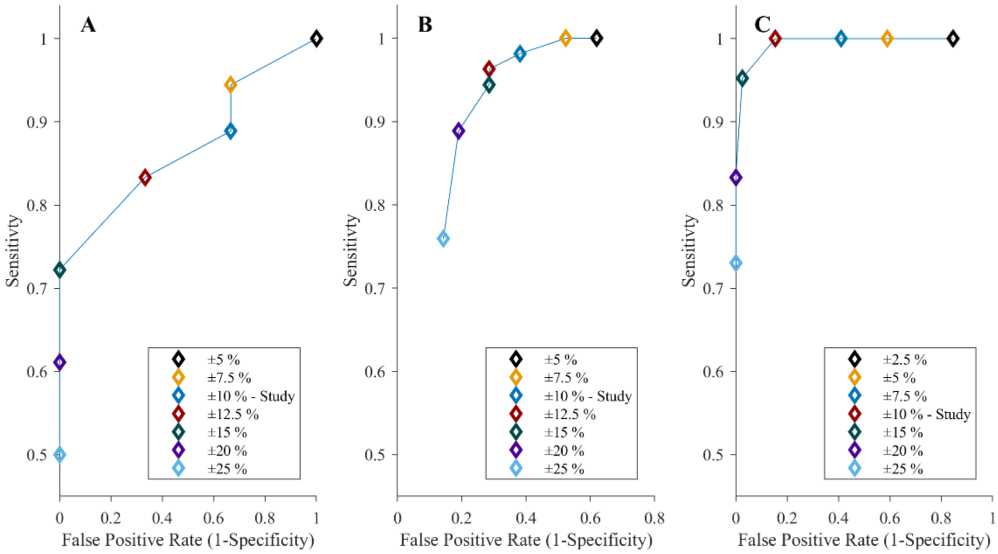

S29 Appendix. Receiver operating characteristic (ROC) curves for quantitative devices.

ROC curves for the **(A)** PharmaChk microfluidic system, **(B)** C-Vue liquid chromatograph, and **(C)** QDa mass spectrometer for all simulated samples containing 50%, 80% and 100% of the correct API. Each point in the plot corresponds to the threshold concentration that would determine a pass or fail result.
