# Supplementary material for: Laboratory evaluation of twelve portable devices for medicine quality screening
Source: PLoS Negl Trop Dis. 2021 Sep 30;15(9):e0009360. doi: 10.1371/journal.pntd.0009360 (PMC8483346; doi:10.1371/journal.pntd.0009360)
Supplement: S30 Appendix — (PDF) [file pntd.0009360.s030.pdf]

S30 Appendix. Limitations for the evaluation

Reference Library Limits:

Reference libraries for the devices were made by recording the spectra of medicine samples which were assumed to be genuine medicines (obtained from large wholesalers or directly from manufacturers). All samples were sent for UPLC analysis, but results were not received until after completion of much of the testing. Some of the samples whose spectra were recorded as reference library entries were found to be poor quality. As a result, we did not have access to good reference library comparators for the affected brands, and it was decided to discard results from testing of all seven affected brands. Reference library creation differed between all instruments due to the wide variety of data capture and software capabilities for each device. There was very limited medicine batch to batch variation in generation of reference library spectra.

Ideally, five different batches or lots are required for a library based on the MicroPHAZIR RX instruction book. How this differs between medicines and devices, and how the number of batches would affect the results of the performances of the devices is unknown. There are also differences in device specific library creation methods when attempting to introduce variability with batch to batch variation. For the NIR-S-G1 and MicroPHAZIR RX some variability was introduced into a single library entry. For the Progeny and Truscan RM, variability was introduced by creating different library entries for different samples.

Device Limits:

The disintegration test available in the Minilab kit was not used in this study which may have resulted in biased performance results. The tests conducted in the laboratory evaluation phase were not conducted blinded to the identity of the quality of the medicines which may have resulted in distortion of the device performance findings. The data analysis for the Neospectra 2.5 was the only one blinded to be comparable to the data processing of the other spectrometers and quantitative devices. At the time of testing the Neospectra 2.5 did not come with pre-built spectra library comparison software. The other spectrometers and quantitative devices had software that output pass/fail results or numeric data with a strict threshold values, limiting bias. Because of time constraints of the project for devices in which operational protocols needed to be developed in the laboratory only basic experiments were conducted. For example, very basic extraction procedures, solvent optimizations, and experimental optimizations were utilized for the C-Vue. Further optimization of these devices would certainly enhance these analyses.

Sample Limits:

Limitations of our study include the limited number of APIs tested (seven), which were all sourced from one region. This represents a small minority of the global medicine

supply and limits the generalizability of these findings. For the simulated medicines, only one batch of samples was available due to the time constraints of the project. For field collected samples, 2-4 batches per medicine were utilized. Different ingredients and batch variations may manifest in difference reference spectra. Assuming a tablet coating is a barrier to interrogate the internal contents of the tablet, analysis of the coated tablet is unlikely to accurately reflect API concentration in the tablet core. This issue is likely to lead to problems with detection of substandard medicines if the degradation/poor manufacturing of the internal contents of the tablet differ from the degradation/poor manufacturing of the coating. For example, if the internal content of the medicine degrades faster than the coating, there may not be a significant signal change in coating analysis to indicate that the sample is suspicious. Coating analysis could potentially scrutinize deviations from the coating of a good quality to a poor quality medicine as poor coatings could degrade faster. Field-collected medicines containing ACA, OFLO, and DHAP had coatings. For the field-collected coated tablet analysis using the non-destructive devices, the medicines were not destroyed to test the internal contents of the medicine. The simulated medicines did not have tablet coatings.

Result Limits:

The non-significant results of the paired comparisons of sensitivity and specificity should be interpreted with caution. For example, the sensitivity of the NIR-S-G1 (91.5%) and that of the 4500a FTIR (100%) were found not significantly different. This is potentially because of the limited sample size to perform this test. Based on these results, the number of samples needed to conclude to a statistical difference of sensitivities, with an alpha error of 5% and a statistical power of 80%, would be at least 90. The results of our study could be used to calculate the appropriate sample size to compare the sensitivity or specificity between different devices.

Using spectrometers, we tested SIM samples containing 0% API against SM samples containing 100% of the API of interest and the same excipients. The NIR-S-G1 wrongly identified SIM 0% API samples as ‘good quality’ when compared to SIM 100% ofloxacin samples (because the ofloxacin peak was slightly out of the spectrum). Falsified medicines are likely to contain different excipients than the authentic medicines, although scientific evidence to support this assumption is lacking. Therefore, it is very likely that the ‘real-life’ sensitivity of the NIR-S-G1 to identify falsified medicines would be higher than that observed in our study. It is important to note that other IR and Raman devices have successfully detected the 0 % API containing samples versus their 100 % API counterparts.
