# Supplementary material for: Laboratory evaluation of twelve portable devices for medicine quality screening
Source: PLoS Negl Trop Dis. 2021 Sep 30;15(9):e0009360. doi: 10.1371/journal.pntd.0009360 (PMC8483346; doi:10.1371/journal.pntd.0009360)
Supplement: Attachment — Submitted filename: Response Reviewers_Medicine Quality Screening_Lab_031621.docx [file pntd.0009360.s032.docx]

***Journal:*** PLOS Neglected Topical Diseases

***Manuscript ID:*** PNTD-D-20-02187

***Title:*** "Laboratory evaluation of twelve portable devices for medicine quality screening."

***Authors:*** Stephen C. Zambrzycki Celine Caillet, Serena Vickers, Marcos Bouza, David V. Donndelinger, Laura C. Geben, Matthew C. Bernier, Paul N Newton, Facundo M. Fernández.

***Collection:*** Article #2 in “A multi-phase evaluation of portable screening devices to assess medicines quality for national Medicines Regulatory Authorities”

Firstly, we thank the reviewers for their thoughtful attention to our manuscript. We agree with many of the reviewer’s comments and suggestions, and we have worked to revise the document to address the remarks. Please consider our best efforts to address the reviewers’ comments below:

**Main Document Comments (added as Comments to Word version):**

***Page 2 – Line 25:*** *Depending on the context, i.e. from the regulatory perspective or from producer/manufacturer or distributor self-market quality surveillance. Pls also elaborate "a key WHAT? A key regulatory function or activity to…? "*

- We have further clarified the context of the opening statement in the background of the abstract. The scope of this paper and collection of articles is to evaluate the tools that could be used by regulators for post-market surveillance.

***Page 2 – Line 35:*** *Would be helpful to readers if a footnote or brief explanation somewhere in the article described how the SIM samples were prepared or based on what principles or standards and guidelines vis-à-vis the formulations that were/are in compliance with GMP standard environment. The formulations prepared in these different environment may not necessarily behave the same way (when using these devices being reviewed to test them) in terms of sensitivity, specificity, and accuracy (of the content),*

- Details about the specific formulations and protocols to prepare the simulated medicines (SIM) are in the S7 Appendix and referenced in page 9 of the main text. The reviewer makes a great point that these SIM may not reflect actual medicines on the market; however, we wanted to minimize the number of variables that could cause a device to misclassify samples with reduced API concentrations. Market medicines may include other ingredients or coatings that could complicate the reasons why a device misclassified a medicine. With our SIM, we could easily determine if a misclassification was the result of the API concentration changing or a change in the bulk excipient. We recognize that these tablets were not made according to GMP and have added this limitation to the limitations paragraph in the Discussion. GMP manufacturing would have been beyond our capabilities'.

***Page 3 – Line 53:*** *The term ‘poor’ in this context may be sensitive to many readers. Could it be replaced by other terms e.g. low-and middle-income countries?*

- We have corrected this by replacing ‘poor’ with ‘low- and middle-income’ countries.

***Page 3 – Line 58:*** *Were the product samples tested were single API only or they include fixed-dose combination product as well? if so perhaps this term should be ingredients, if both cases ingredient(s).*

- We have corrected this by stating ‘ingredient(s)’ as we have tested medicines with a single API and co-formulations.

***Page 8 – Line 134-135:*** *Were these samples still in their shelve-life i.e. not yet expired?*

- The context of this comment is for the falsified field collect medicines (FCM). Since the FCM were falsified, an accurate shelf-life determination cannot be made as the falsified packaging lists the expiration date of original good quality product containing the correct API or a fictional expiry date. The falsified FCM we tested did not contain the API and were sugar tablets. After the study referenced [24], the falsified FCM were stored in a cool, dry, and dark place until this study.

***Page 9 – Line 151-153:*** *These two sentences seem a bit confusing. They seem not being linked with each other. The 2nd sentence ‘These FCM tablets….’ Should read ‘These SIM tablets….?*

- We addressed the confusion by stating the FCM tablets were crushed and repressed into SIM tablets.

***Page 9 – Line 155-156:*** *Pls explain why magnesium stearate was excluded?*

- The context of this comment is for the simulated artesunate sample. We have further clarified this statement by adding that this was to simulate an intravenous medicine. This artesunate medicine typically comes as a loose powder and does not require magnesium stearate as a lubricant for the tablet press.

***Page 13 – Line 204:*** *..for all APIs included sounds that the combined content of the APIs is counted. Should this be for each of the APIs? And that if any of them does not meet the 90%-110% range the sample fails quality testing****.***

- We have added another sentence to the commented statement to clarify. For co-formulations, if at least one of the API was outside the 90-110% range, the entire medicine would be deemed outside specification.

***Page 13 – Line 218:*** *Would be helpful to define what ‘best’ mean in this context?*

- We have further clarified by also stating it is a 2/3 majority result.

***Page 26 – Line 423:*** *Would be helpful to discussed about the price and cost of each of the devices as well for the interested countries in consideration to introduce any of them in the PMS program.*

- A detailed cost-effectiveness analysis for a selection of the devices deemed field suitable from this paper is evaluated in the “Implementation of Field Detection Devices in Medicine Quality Screening in Lao PDR – A Cost-Effectiveness Analysis”, the 4^th^ article in our collection. Initial purchase prices for these devices are listed in the S4 Appendix of the current article and we will more explicitly state that in the main text accompanying table 1 of the revised manuscript. Further details about the cost of devices in the main text may be outside the scope of this paper as prices vary based on number of units purchased, development stage of the technologies, and import/export costs.

**Reviewer’s Responses to Questions:**

***Review #2 states*** *“To include cost of device, approximate cost and time duration in performing one test in TABLE-1.” & “In Discussion, to include cost effectiveness of each testing method.”*

- A detailed cost-effectiveness analysis for a selection of the devices deemed field suitable from this paper is evaluated in the “Implementation of Field Detection Devices in Medicine Quality Screening in Lao PDR – A Cost-Effectiveness Analysis”, the 4^th^ article in our collection. Initial purchase prices for these devices are listed in the S4 Appendix and we will more explicitly state that in the main text of our revised manuscript. Further details about the cost of devices in the main text may be outside the scope of this paper as prices vary based on number of units purchased, development stage of the technologies, and import/export costs. We believe that the time duration testing is more accurately reflected when measured while the devices are in the hands of final users. This was evaluated in more details in the 3^rd^ article of the PLOS collection, “A comparative field evaluation of six medicine quality screening devices in Laos”. In this paper, a time and motion study was conducted with the devices in the hands of medicines inspectors from the Laotian government.

***Review #2 states*** *“In Discussion, to discuss on performance of device in point-of-care or field settings. If some devices needs laboratory facility or temperature needs to be maintained, do make a mention about in limitations section.”*

- Field testing with pharmacy inspectors for a selection of the devices deemed field suitable is detailed in “A comparative field evaluation of six medicine quality screening devices in Laos”, the 3^rd^ article in our collection. More details are added to the last paragraph of the discussion to describe which devices are more appropriate for laboratory environments. The quantitative devices would be more laboratory suitable because the consumable requirements would make transportation difficult.
